# Identification of Putative Target Genes of the Transcription Factor *RUNX2*

**DOI:** 10.1371/journal.pone.0083218

**Published:** 2013-12-12

**Authors:** Martin Kuhlwilm, Armaity Davierwala, Svante Pääbo

**Affiliations:** Max Planck Institute for Evolutionary Anthropology, Leipzig, Germany; Univeristy of California Riverside, United States of America

## Abstract

Comparisons of the genomes of Neandertals and Denisovans with present-day human genomes have suggested that the gene *RUNX2*, which encodes a transcription factor, may have been positively selected during early human evolution. Here, we overexpress *RUNX2* in ten human cell lines and identify genes that are directly or indirectly affected by *RUNX2* expression. We find a number of genes not previously known to be affected by RUNX2 expression, in particular *BIRC3*, genes encoded on the mitochondrial genome, and several genes involved in bone and tooth formation. These genes are likely to provide inroads into pathways affected by *RUNX2* and potentially by the evolutionary changes that affected *RUNX2* in modern humans.

## Introduction

Genome sequences of the closest extinct evolutionary relatives of present-day humans, Neandertals [1] and Denisovans [2,3], allow genomic changes that occurred recently during human evolution to be identified. In addition, they allow putative selective sweeps that occurred early during modern human evolution to be detected. This is because Neandertals and Denisovans are so closely related to present-day humans that a majority of their genomes falls into the variation of present-day humans. As a result long genomic regions where all present-day human genomes are more closely related to each other than to the archaic genomes will tend to have been affected by positive selection in modern humans after their separation from an ancestor shared with Neandertals and Denisovans. In an initial screen for positive selection based on this approach, 20 top regions were identified [1], one of which is located on chromosome 6, and contains the gene *RUNX2*. In another screen for positive selection that occurred early during the evolution of modern humans, which is based on the observation of unusually long stretches of high-frequency derived variants shared among distantly related extant human populations, the largest region identified (~ 900kb) contained *RUNX2* [4].

The fact that *RUNX2* appears to have been affected by positive selection early after the divergence of modern humans from their archaic relatives may be of relevance with respect to the evolution of the cranium and skeleton of modern humans. *RUNX2* encodes a transcription factor that is crucial for osteoblast differentiation [5], mesenchymal bone development [6], and fontanel closure [7,8]. Heterozygous loss-of-function mutations in *RUNX2* cause cleidocranial dysplasia [9], a syndrome characterized by delayed closure of cranial sutures, protruding frontal bone (so-called frontal bossing), abnormalities of the clavicle, and a bell-shaped rib cage [10]. Interestingly, some of the skeletal features affected in cleidocranial dysplasia differ between modern humans and earlier, archaic forms of humans, including Neandertals. For example, closure of the fontanels may have been delayed in modern humans [11]; more protruding frontal bones are seen in modern humans relative to archaic hominins [12]; the clavicle differs in morphology between modern and archaic humans [13]; and a bell-shaped rib cage is typical of Neandertals, and other archaic hominins. A reasonable hypothesis is that one or more evolutionary changes that affected *RUNX2* during the evolution of early modern humans contributed to one or more of these morphological differences. Since no amino acid differences exist between the RUNX2 protein in present-day humans and the Neandertal and Denisova genomes, any such change is likely to have affected the expression of *RUNX2* during development.

*RUNX2* is transcribed from two promoters, P1 and P2. These cause expression of protein isoforms that differ in their N-terminal amino acids, and the corresponding transcripts have different 5’ untranslated regions [14]. If the P1 promoter is deleted, endochondral bone formation is affected, i.e. bone formation which occurs from cartilage. If the whole *RUNX2* gene, including the P2 promoter, is knocked out [15], mesenchymal bone formation is also affected, i.e. a large part of the clavicle and most of the bones of the skull. Interestingly, it has also been demonstrated that the RUNX2 isoform expressed from the P2 promoter is expressed in cranial sutures [16].

If *RUNX2* changed its role during recent human evolution, the effects are likely to have been mediated through its downstream target genes. This consideration, as well as the importance of *RUNX2* for bone development in general, prompted us to investigate how *RUNX2* gene expression influences gene expression in human cells. To date, one genome-wide study has investigated binding of RUNX2 to the promoter region of genes in one cell line [17]. To achieve a more comprehensive understanding of which genes might be directly and indirectly influenced by RUNX2, we have overexpressed the RUNX2 isoform expressed from the P2 promoter in ten human cell lines and analyzed the resulting change of expression by deep sequencing of their transcriptomes. 

## Materials and Methods

### Cloning and plasmid preparation

A plasmid containing the *RUNX2* cDNA was obtained from the Mammalian Gene Collection (MGC:193137, IMAGE:100063958) through *Imagenes* (now *SourceBio*). The cDNA insert was amplified using *HotStar* polymerase (*Qiagen*).

Cloning into an expression vector (pcDNA3.2/V5-DEST) was performed using the Invitrogen *Gateway* cloning system (BP: 11789, LR: 11791). We verified insert sequences of five clones using the *ABI 3730 DNA Analyzer* with *BigDye Terminator* v3.1 (25 cycles, annealing temperature 55°C) and the following primers: 5’-CGTGTACGGTGGGAGGTCTA-3’, 5’-aaggcacagacagaagcttga-3’, 5’-ttacttacaccccgccagtc-3’, 5’-tcgttgaaccttgctacttgg-3’, 5’-ggtggcagtgtcatcatctg-3’, 5’-gtcgccaaacagattcatcc-3’, 5’-AGACCGAGGAGAGGGTTAGG-3’. The *RUNX2* containing plasmids were purified using the Qiagen *EndoFree Plasmid* Maxi Kit (12362) and diluted to 1µg/µL. Three clones carrying the correct inserts were isolated, and used for the experiments performed in triplicates.

### Cell culture

The human cell lines, hFOB1.19 (Osteoblastoma, CRL-11372™), Saos-2 (Osteosarcoma, HTB-85™), U-2 Os (Osteosarcoma, HTB-96™), SK-N-SH (Neuroblastoma, HTB-11™), IMR-32 (Neuroblastoma, CCL-127™), U-87 MG (Astrocytoma, HTB-14™), ACHN (Adenocarcinoma, CRL-1611™), HepG2 (Hepatoma, HB-8065™), HeLa-S3 (Cervical cancer, CCL-2.2™), were obtained from the American Type Culture Collection (ATCC, http://www.lgcstandards-atcc.org), and the cell line SH-SY5Y (Neuroblastoma, 94030304) from the European Collection of Cell Cultures (ECACC, http://www.hpacultures.org.uk). All cell lines were cultured as recommended by the supplier. Media, fetal bovine serum and penicillin/streptomycin were obtained from *Invitrogen*. Culture media were changed as recommended, at least every third day.

### Transfection

Transfections were performed using the *Amaxa Nucleofector^TM^* system, according to the recommendations of the supplier, using the *RUNX2* expression vector (two replicates of each insert-verified vector, in total six replicates per cell line), and the control expression vector pcDNA3.2/GW/CAT supplied with the Invitrogen *Gateway* system (three replicates per cell line). For each cell line, a control transfection with the pmax-GFP (provided with the *Amaxa Nucleofector^TM^* kits) plasmid alone, and a co-transfection with the pmax-GFP plasmid along with the *RUNX2* expression vector, were performed. For each transfection, cells were resuspended in 100µL *Nucleofector* solution, 2µg of the plasmid was added, the electroporation settings recommended for the cell line by *Amaxa* were applied and 500µL medium was added (detailed conditions in Table S7). Transfected cells were grown in their respective media for 11-48h until a majority of cells showed visible GFP expression.

### RNA purification

Cells were lysed using the RLT buffer from the *RNeasy* kit (*Qiagen* 74106), homogenized using *QiaShredder* columns (*Qiagen* 79656), and total RNA was isolated using the *RNeasy* kit. RNA concentration and quality was assayed using the *RNA 6000 Nano kit* (*Agilent* 5067-1511) on an *Agilent 2100 BioAnalyzer*.

### Reverse transcription and qPCR

We assayed expression of *RUNX2* and *GAPDH*, which is expected to be constitutively expressed, and can thus be used to normalize expression differences between samples [18] by quantitative PCR. Reverse transcription was performed using the *DyNAmo cDNA Synthesis* kit (*NEB Finnzymes F-470*) with random hexamer primers, using the *DyNAmo HS SYBR Green* qPCR kit (*NEB Finnzymes F-410XL*) on a *Mx3005Pro* system (*Agilent*). We detected an increase in the ratio of *RUNX2* to *GAPDH* expression between *RUNX2* transfected and mock transfected cells. In all cell lines, *RUNX2* overexpression of the same magnitude was also detected for the samples with co-transfection of a GFP-containing plasmid. Primers used for qPCR for *RUNX2* were as follows: 5’-GACAGCCCCAACTTCCTGT-3’ (forward) and 5’-TCGTTGAACCTTGCTACTTGG-3’ (reverse), and for *GAPDH*: 5’-AGAAGGCTGGGGCTCATTTG-3’ (forward) and 5’-AGGGGCCATCCACAGTCTTC-3’ (reverse).

### Library preparation and sequencing

Sequencing libraries were prepared using 2µg or 3µg of RNA (depending on the amount available) using a modified version of the *GA*_*IIx*_ protocol from *Illumina*. The mRNA fraction was purified from total RNA using *Sera-Mag oligo*(*dT*)*Beads* (*ThermoScientific*, 6515-2105-050250) followed by an additional cleaning step using *RNAClean XP* beads (*Agencourt*, A63987), chemically fragmented using a zinc-containing reagent (*ABI*, AM8740) for 3 minutes. First strand cDNA synthesis was performed using *SuperScript II* (*Invitrogen*, 18064014) and second strand synthesis using *DNA Polymerase I* (*Fermentas EP0042*). Blunt-end repair of the double-stranded cDNA fragments, ligation to adapter molecules and fill-in of nicks were performed as previously described [19], followed by purification using *SeraMag SpeedBeads* (*ThermoScientific*, 6515-2105-050250). Finally, index sequences were added to the fragments using *Phusion HotStart High-Fidelity Polymerase* (*NEB*, F-549). Libraries were sequenced on four flow cells of the Illumina *GA*_*IIx*_ system (v4 chemistry, v2 cluster generation kit) for 76 cycles. Two lanes were sequenced for each of the cell lines, U-87 MG, IMR-32, ACHN, SK-N-SH, three lanes for each of SH-SY5Y, HeLa-S3, HepG2, Saos-2 and U-2 Os and six lanes for hFOB1.19.

### Data processing

Base calling was performed using *Ibis* [20]. The raw reads were filtered for the presence of the correct library indexes and a minimum base quality score of 10. The adapters were removed after filtering. Reads were aligned to the human genome (version hg19, 1000 Genomes Project) using *Tophat* 1.3.0. A fraction of 47%-75% of fragments was mapped, with an average of 57%. The number of mapped fragments per sample ranged from 2,836,317 to 21,718,800 with a median of 9,034,297.

Fragments per gene (defined as in the ENSEMBL GRCh37.62 release) were counted using *HTseq-count* 0.5.3p3 (http://www-huber.embl.de/users/anders/HTSeq/doc/overview.html), and genes were considered to be expressed in a cell line if at least one read was aligned to the gene sequence in at least three replicates. Differential expression was tested using the deviation from a negative binomial distribution [21] in *DESeq* 1.0.6 in R/Bioconductor [22]. We used an adjusted p-value cut-off of 0.01 after Benjamini-Hochberg correction for multiple testing, as provided by *DESeq*. Gene information was obtained and conversions between annotations were performed using the *biomaRt* package for R [23,24].

**Figure 1:**
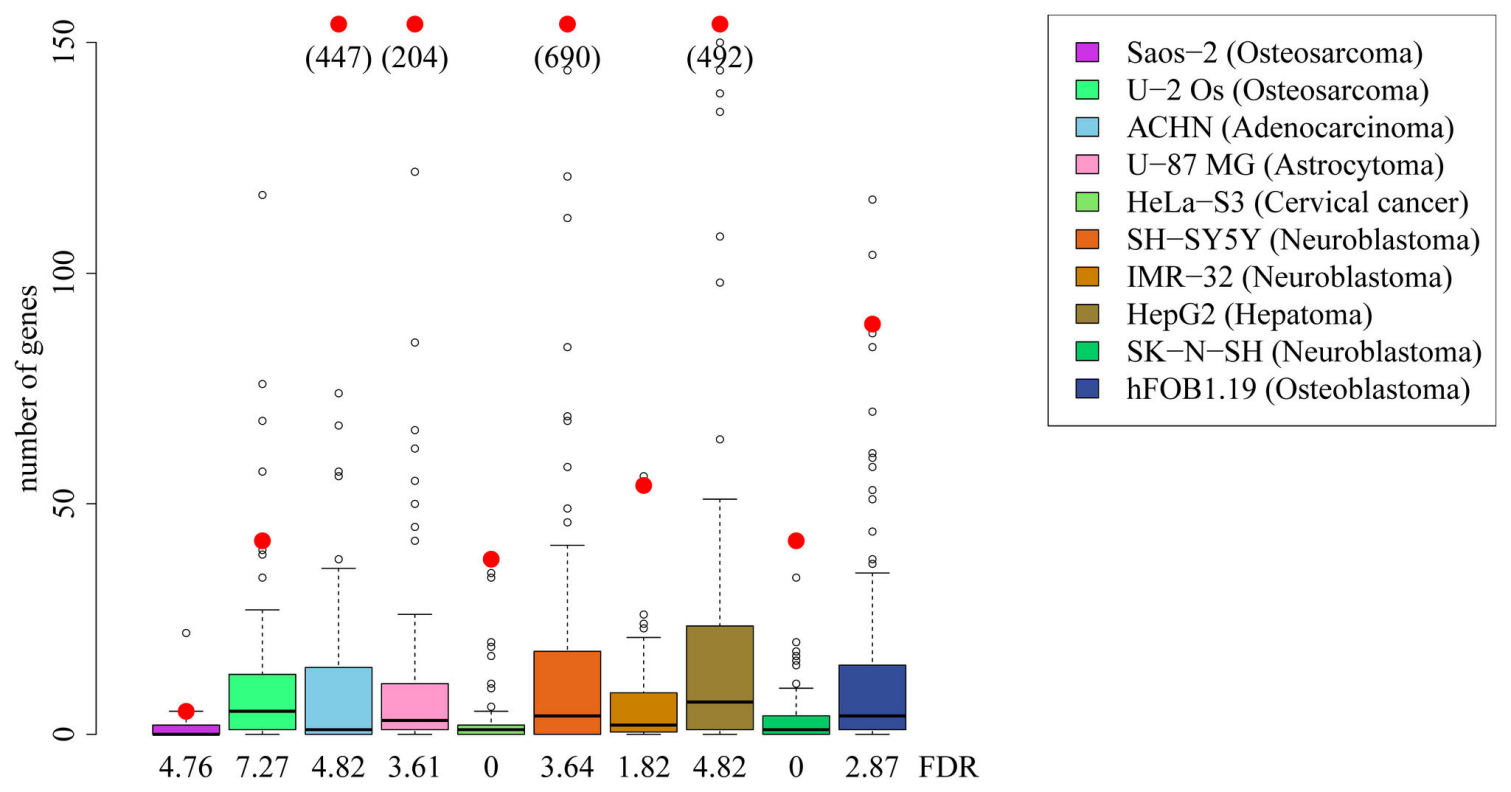
Differentially expressed genes. Number of differentially expressed genes after transfection with *RUNX2* (red dots), compared to number of genes after permutation of sample labels. Note that values for differentially expressed genes above 150 are shown in brackets.

**Figure 2:**
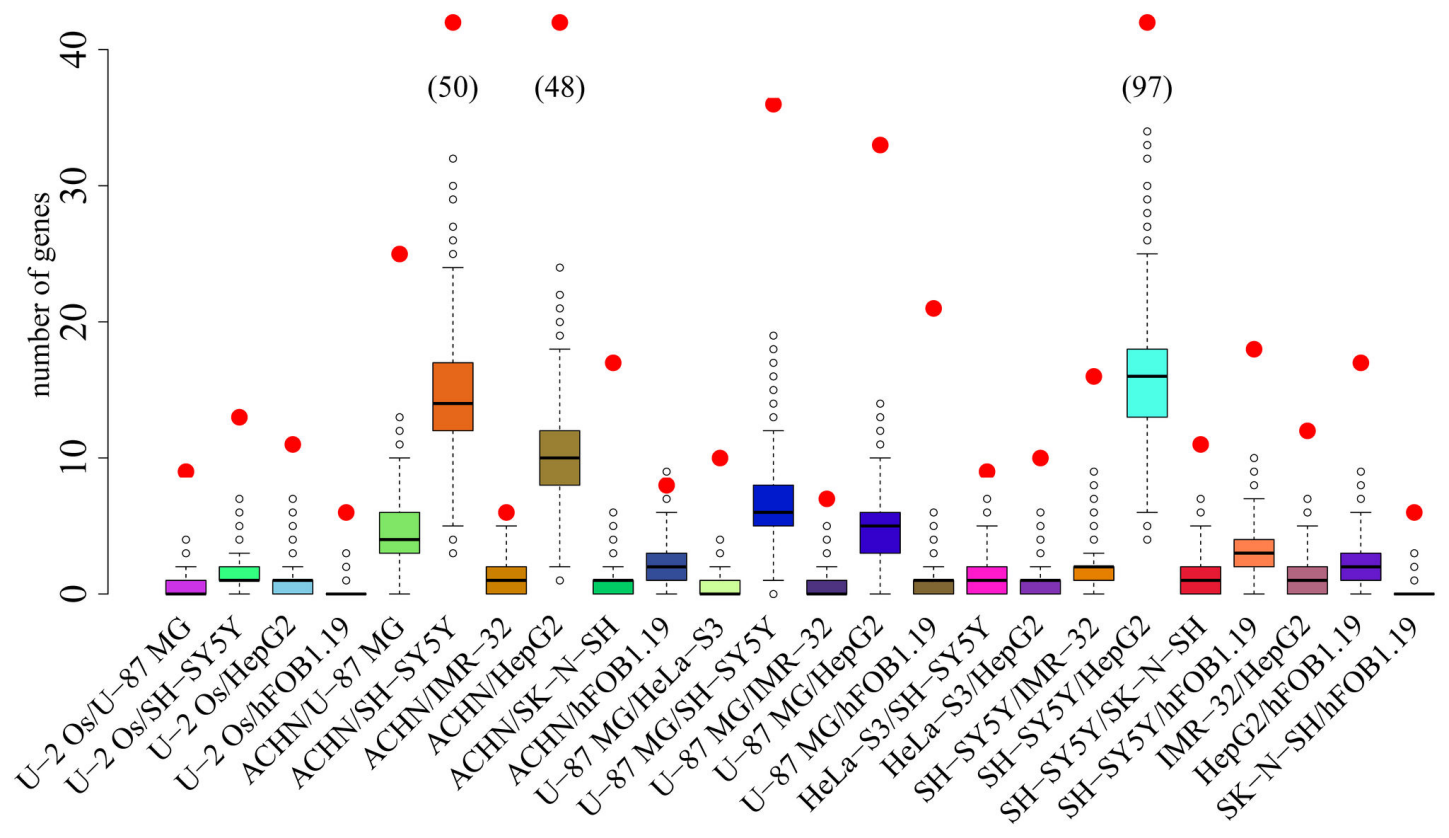
Overlaps of differentially expressed genes between cell lines. Numbers of genes that differ in expression after RUNX2 overexpression in two cell lines (red dots), and distribution of such overlaps between sets of the same numbers of random genes expressed in the cell lines. If the number of genes overlapping between a pair of cell lines was smaller than five, it is not shown.

The mean Spearman correlations of overall expression between mock-transfected cell cultures and cell cultures transfected with RUNX2 within a cell line are >0.99, while the Spearman correlation of gene expression between the different cell lines without RUNX2 transfection ranges from 0.67 (ACHN vs. IMR-32) to 0.90 (SH-SY5Y vs. SK-N-SH, which are subclones of the same original neuroblastoma) (Figures 1, 2, 3).

### GO analysis

GO analysis was performed using the hypergeometric test in the software *FUNC* [25] with a p-value cut-off for significance of 0.1 and a family-wise error rate of 0.1. We excluded all categories that contained less than 5 genes as well as categories in which more than 90% of genes were also contained in significant categories with lower p-values.

### Genomic analysis

We used the catalog of differences between humans and the Denisova individual [3], containing alignments for other primates including chimpanzee, and information about frequencies of alleles in human populations from the 1000 Genomes Project 20110521 release [26]. 

## Results

### *RUNX2* overexpression

A cDNA clone, encoding the protein expressed from the P2 promoter of *RUNX2* (“isoform I”), was cloned in a vector, where it is expressed from the cytomegalovirus (CMV) immediate early promoter. This vector was used in transfections of ten cell lines: the osteoblastoma, hFOB1.19; the osteosarcomas, Saos-2, U-2 Os; the neuroblastomas, SH-SY5Y, SK-N-SH, IMR-32; the astrocytoma, U-87 MG; the adenocarcinoma, ACHN; the hepatoma, HepG2; and the cervical carcinoma, HeLa-S3. 

Six replicate cultures of each cell line were transfected with 2µg of the *RUNX2* expression vector. In parallel, three replicate cultures were transfected with a control vector that is identical to the expression vector except that it lacks the *RUNX2* insert. In addition, one culture of each cell line was transfected with a vector expressing green fluorescent protein under the CMV promoter to monitor transfection efficiency. After 11-48 hours, total RNA was isolated from each culture, and the expression of *RUNX2* was analyzed by quantitative PCR. From cultures that showed an increase in *RUNX2* expression relative to a house-keeping gene (*GAPDH*) between transfected and mock transfected cells, mRNA was purified, indexed cDNA libraries were constructed, and sequenced on the Illumina GA_IIx_ platform. 

The number of uniquely mapped fragments per library ranged from 2,836,317 to 21,718,800 with a median of 9,131,168, and the total number of mapped fragments per cell line ranged from 70,340,341 (U-87 MG) to 225,400,072 (hFOB1.19). Expression of between 17,510 (HeLa-S3) and 21,017 (hFOB1.19) genes (as defined by unique ENSEMBL gene IDs) per cell line was detected. Expression of 13,131 genes was detected in all cell lines, while the expression of an average of 374 genes was detected only in single cell lines.

### *RUNX2* expression

Seven of the cell lines (ACHN, HeLa-S3, hFOB1.19, Saos-2, SK-N-SH, U-2 Os, U-87 MG) expressed *RUNX2* also in the mock transfected cells. In those cell lines, transfection of the expression vector resulted in a 1.6- to 74.2-fold increase in *RUNX2* expression (Table 1). In the remaining cell lines, transfection of the expression vector resulted in *RUNX2* mRNA expression at levels comparable to the expression in the cell lines with endogenous *RUNX2* expression.

### Differential gene expression

To identify genes that changed their expression as a result of *RUNX2* expression we compared the average expression level of each gene in the six replicated cultures where *RUNX2* was overexpressed with the average of the expression level in the three mock-transfected cultures for each cell line. Genes that were differently expressed (Benjamini-Hochberg corrected p-value <0.01 using a negative binomial distribution [21]) were further analyzed. 

To estimate a false discovery rate for each cell line, we switched the “labels” of cultures with respect to if they were transfected with the *RUNX2* vector or mock transfected for all possible combinations of labels, and determined the number of differentially expressed genes using the same test for all combinations of labels not identical with the real data. In all 10 cell lines, the number of genes differentially expressed in the permutations is less than 10% of the number seen in the real data, suggesting that the majority of expression differences detected are due to transfection of the *RUNX2* vector (Figure 1).

In total, 1,715 genes were differentially expressed in at least one of the 10 cell lines (Table 2). Two hundred and eighty eight genes were differentially expressed in at least two cell lines, 68 were differentially expressed in at least three cell lines, 24 in four cell lines (Table 1), eight in five cell lines, and one gene (*BIRC3*) was differentially expressed in seven cell lines.

**Table 1:** Genes differentially expressed in four or more cell lines after *RUNX2* overexpression.

**Gene name**	**Number of cell lines**	**Description**
***BIRC3***	7	baculoviral IAP repeat containing 3
***ATF3***	5	activating transcription factor 3
***EGR1***	5	early growth response 1
***MALAT1***	5	metastasis associated lung adenocarcinoma transcript 1 (non-protein coding)
***MT-ATP6***	5	mitochondrially encoded ATP synthase 6
***MT-CYB***	5	mitochondrially encoded cytochrome b
***MTATP6P1***	5	mitochondrially encoded ATP synthase 6 pseudogene 1
***NEAT1***	5	nuclear paraspeckle assembly transcript 1 (non-protein coding)
***AHNAK***	4	AHNAK nucleoprotein
***CD82***	4	CD82 molecule
***DUSP10***	4	dual specificity phosphatase 10
***HMOX1***	4	heme oxygenase (decycling) 1
***IL11***	4	interleukin 11
***KLF9***	4	Kruppel-like factor 9
***LRP1***	4	low density lipoprotein receptor-related protein 1
***MEGF10***	4	multiple EGF-like-domains 10
***MT-CO3***	4	mitochondrially encoded cytochrome c oxidase III
***MT-ND1***	4	mitochondrially encoded NADH dehydrogenase 1
***MT-ND4L***	4	mitochondrially encoded NADH dehydrogenase 4L
***MT-ND5***	4	mitochondrially encoded NADH dehydrogenase 5
***MT-ND6***	4	mitochondrially encoded NADH dehydrogenase 6
***NFKBIE***	4	nuclear factor of kappa light polypeptide gene enhancer in B-cells inhibitor, epsilon
***PPP1R15A***	4	protein phosphatase 1, regulatory subunit 15A
***UNC5B***	4	unc-5 homolog B (C. elegans)

To test the extent to which the same genes tend to be affected by *RUNX2* expression in different cell lines, we randomly sampled the same number of genes as those that were differentially expressed from among all expressed genes in each cell line, and tested the overlap between all possible pairs of cell lines (Figure 2). For each pair, we did 10,000 comparisons among the random sets of genes. For 35 out of the 45 pairwise combinations of cell lines, the observed number of genes differentially expressed in both the cell lines was five or more standard deviations above the mean of the random sets of expressed genes.

To test whether genes change their expression as a result of *RUNX2* expression in the same direction in the different cell lines, we compared the normalized expression of each gene after *RUNX2* transfection to the expression in the mock transfected cells, and assigned the value “1” for higher and “0” for lower expression, respectively. The average value of all genes irrespective of whether they were affected by *RUNX2* overexpression or not within a given cell line varied between 0.49 and 0.52 (Table S3). The average for the 1,715 genes that were differentially expressed in at least one cell line varied between 0.43 and 0.58. For genes that affected the expression in more cell lines the variance increases progressively, so that the eight genes that are affected by *RUNX2* overexpression in five cell lines vary between zero and one. In eight of the ten cell lines, there is a tendency for RUNX2 to increase rather than decrease the expression of genes, in the adenocarcinoma cell line, ACHN, expression changes are not correlated with each other, and in the neuroblastoma line, IMR-32, *RUNX2* expression tends to decrease the expression of genes. An interesting question for future work is why *RUNX2* tends to decrease gene expression in the latter cell line, while in most other cell lines, including two other neuroblastoma cell lines, it tends to increase gene expression. 

### Comparison to promoter occupancy

When compared to a list of 1,753 genes for which RUNX2 binding to the promoters has been shown in Saos-2 osteosarcoma cells [17], 27 of the 288 genes that are differentially expressed in two or more cell lines overlap (Table S4). This extent of overlap is seen in 7.2% of 1,000 random sets of 288 genes out of all genes with expression in at least two cell lines. When compared to a list of 68 genes that changed their expression after siRNA-induced knock-down of RUNX2 in Saos-2 and U-2 Os cells [17], three genes out of the 288 overlap (*EFNB2*, *EGR1, SNAPC1*). One of these, *EGR1*, is differentially expressed in five cell lines and the others in two cell lines.

## Discussion

In Table 1 we list genes whose expression is significantly changed in four or more of the ten cell lines after RUNX2 over-expression. These genes are likely to be directly or indirectly affected by RUNX2. Among them, *BIRC3* stands out in being differentially expressed in seven cell lines. In six of these, it is up-regulated (HeLa-S3, HepG2, hFOB1.19, SH-SY5Y, U-2 Os, U-87 MG), while it is down-regulated in one (ACHN) (Table 2). In two other cell lines (Saos-2, SK-N-SH), *BIRC3* shows an increase in expression below significance (raw p-values, p=0.05 and p=0.92, respectively) while no expression could be detected in IMR-32. *BIRC3* encodes a member of a family of proteins that inhibit apoptosis by binding to the tumor necrosis factor receptor-associated proteins TRAF1 and TRAF2 [27]. Notably, *TRAF1* is significantly up-regulated in two (HepG2, SH-SY5Y), and down-regulated in one of the cell lines analyzed (ACHN). The differences in *TRAF1* expression have the same direction in the same cell lines as the differences seen for *BIRC3*. In addition, *BIRC3* is involved in multiple other functions, for example, inflammation, immunity, and cell proliferation, often through the NF-κB signaling pathway [28]. *BIRC3* has also been shown to be up-regulated in some osteosarcomas [29].

**Table 2:** Expression differences observed for *BIRC3* and mitochondrially encoded genes.

**Genes**	**Saos-2**	**U-2 Os**	**ACHN**	**U-87 MG**	**HeLa-S3**	**SH-SY5Y**	**IMR-32**	**HepG2**	**SK-N-SH**	**hFOB1. 19**
***BIRC3***	˄	˄ *	˅ *	˄ *	˄ *	˄ *	NA	˄ *	˄	˄ *
***MT-ATP6***	˅	˅ *	˄ *	˅ *	˄	˄ *	˅	˄ *	˄	˄
***MT-ATP8***	˅	˅	˄ *	˅	˄	˄ *	˅	˄ *	˄	˅
***MT-CO1***	˅	˅	˄ *	˅ *	˄	˄	˅	˄ *	˄	˄
***MT-CO2***	˅	˅	˄	˅ *	˄	˄ *	˅	˄	˄	˄
***MT-CO3***	˅	˅	˄ *	˅ *	˄	˄ *	˅	˄ *	˄	˄
***MT-CYB***	˅	˅	˄ *	˅ *	˄	˄ *	˅ *	˄ *	˄	˄
***MT-ND1***	˅	˅	˄ *	˅ *	˄	˄ *	˅	˄ *	˄	˄
***MT-ND2***	˅	˅	˄	˅	˄	˄ *	˅	˄ *	˄	˄
***MT-ND3***	˅	˅	˄ *	˅	˅	˄	˅	˄	˄	˄
***MT-ND4***	˅	˅	˄ *	˅	˄	˄ *	˅	˄ *	˄	˄
***MT-ND4L***	˅	˄	˄ *	˅	˄	˄ *	˅ *	˄ *	˄	˅
***MT-ND5***	˅	˄	˄ *	˅ *	˄	˄	˅ *	˄ *	˄	˄
***MT-ND6***	˅	˄	˄ *	˅ *	˄	˄	˅ *	˄ *	˄	˄
***MT-RNR1***	˅	˅	˄	˄	˄	˄	˅	˄ *	˄	˄
***MT-RNR2***	˄	˅	˄	˄	˄	˄	˄	˄	˄	˄
***MT-TC***	˄	˅	˄ *	˅	˄	˄	˅	˄	˄	˄
***MT-TF***	˅	NA	˄	NA	˅	˄	˅	˄	NA	˄
***MT-TL1***	˄	NA	˅	NA	˄	˄	˅	˄	˅	˅
***MT-TP***	˄	˄	˄	˅	˅	˄	˅	˄ *	˄	˅
***MT-TY***	˅	˅	˄	˅	˅	˅	˅	˄	˄	˄

Direction of gene expression: ˄ upregulated after RUNX2 transfection, ˅ downregulated after RUNX2 transfection, * denotes significant expression differences (*DESeq*, p-value < 0.01) and NA absence of expression.

While no genes were significantly affected by *RUNX2* over-expression in six of the cell lines, seven genes showed a significant difference in expression in five cell lines. Two of these are encoded on the mitochondrial genome (*MT-ATP6, MT-CYB*). In addition, the expression of five other mitochondrially encoded genes (*MT-CO3, MT-ND1, MT-ND4L, MT-ND5, MT-ND6*) is affected in four of the cell lines. Although the expression of these genes is both up- and down-regulated, they correlate within a cell line with respect to their direction of change, and mostly with the expression of other mitochondrially encoded genes (Table 2). It is thus clear that in several cell types, RUNX2 influences the cellular abundance of mitochondrial transcripts. Further work is necessary to elucidate the presumably indirect mechanisms by which this occurs. The other five genes affected in five cell lines are two transcription factors: *ATF3*, which is involved in apoptosis [30], and *EGR1*, a growth response factor involved in differentiation [31], two large non-coding RNAs (*MALAT1, NEAT1*), and an autosomal pseudogene (*MTATP6P1*). 

Sixteen genes are affected by *RUNX2* expression in four cell lines. Among these are a number that are involved in the development of bone and teeth, for example *IL11*, encoding an interleukin involved in the development of craniofacial bones and teeth and the closure of the cranial sutures [32], and *LRP1*, encoding a low-density lipoprotein receptor-related protein also involved in chondrocyte differentiation [33]. In addition, three of the 16 genes have roles in apoptosis; *UNC5B* [34], *MEGF10* [35] and *PPP1R15A*, which encodes a regulator of protein phosphatase 1 [36,37]. Furthermore, *NF-κBIE* is down-regulated in three and up-regulated in one cell line. This may be of interest since NF-*κ*BIE is an inhibitor while BIRC3 may act as an activator of NF-κB, which in turn has been shown to be involved in bone development [38].

Among 43 genes that are differentially expressed in three cell lines, we find three collagen genes, and two metallo-matrix protease genes, *MMP9* and *MMP13*. Both are previously known targets of RUNX2 [39,40], and encode proteins involved in bone development [41]. 

A Gene Ontology analysis of the genes that changed in expression after *RUNX2* over-expression in at least two cell lines shows, among 18 other biological categories, enrichment of genes involved in skeletal system development and apoptotic processes (Table S5).

In summary, we show that a number of genes that were previously not known to be regulated by RUNX2 are directly or indirectly affected by *RUNX2* expression, particularly *BIRC3*, several genes encoded in the mitochondrial genome, and several genes involved in bone and tooth development.

We note that several positions in the *RUNX2* region carry derived alleles of high frequency in present-day humans (>90% derived in humans) while the Denisova [3] and Neandertal genomes (http://www.eva.mpg.de/neandertal/index.html), are both homozygous for the ancestral alleles (Table S6). While no such positions are located in the P1 promoter, two are found in the P2 promoter, close to the transcription start of “isoform I” (rs7751427 and rs7771980). They are in strong linkage disequilibrium with each other and with polymorphisms in exon 2 [42]. The derived allele at rs7771980 has been associated with lower bone mineral density in humans [43], and lower promoter activity after transfection of a reporter construct into an osteoblast-like rat cell line [42], but also with higher bone mineral density in another study [44]. Nevertheless, it is likely that *RUNX2* has changed its expression patterns from the P2 promoter during recent human evolution and it is possible that this may be the causal change that underlies the selective sweep signal in the *RUNX2* gene. It will be interesting to study the expression of the genes identified here in cell lines and tissues from individuals that carry the ancestral and derived alleles at the P2 promoter of the *RUNX2* gene, as well as the morphological phenotypes. 

## Supporting Information

Figure S1 **Pairwise distance matrix of overall mean expression values in control transfections between cell lines.** The hepatoma cell line differs the most from the other cell lines in terms of its response to RUNX2 overexpression. The osteoblastoma and one of the osteosarcoma cell lines are clustering with each other.
(TIF)

Figure S2 **Correlations of expression between pairs of cell lines.** Mean expression of genes in control transfection. Values are log_2_-transformed. Spearman correlations are between 0.67 (IMR-32/HepG2) and 0.9 (SK-N-SH/SH-SY5Y).
(TIF)

Figure S3 **Correlations of expression within cell lines.** Mean expression of genes after RUNX2 overexpression and mean expression of genes in control transfection. Expression of RUNX2 is given in red. Values are log_2_-transformed. Spearman correlations are above 0.99.
(TIF)

Table S1 **Expression of RUNX2 in transfected and mock transfected cell lines and number of differentially expressed genes.** Average expression levels in raw count values are normalized to the relative library sizes using *DESeq*.
(DOCX)

Table S2 **The 1,715 genes found to be differentially expressed after *RUNX2* transfection in 10 cell lines.** Genes are sorted by the number of cell lines in which they were found to be differentially expressed.
(DOCX)

Table S3 **Average directions of expression differences between *RUNX2* transfected and mock transfected cells.** The values 1 and 0 indicate higher and lower expression of a gene after *RUNX2* transfection, respectively. Average numbers across all genes and differentially expressed genes that overlap between cell lines.
(DOCX)

Table S4 **List of 27 genes to which RUNX2 was found to bind by chromatin immunoprecipitation and genes found to be differentially expressed in the present study in two or more cell lines after *RUNX2* overexpression.**
(DOCX)

Table S5 **Categories in the “Biological Process” gene ontology, which are enriched among genes differentially expressed in two or more cell lines after overexpression of *RUNX2*, using a hypergeometric test implemented in *FUNC*.**
(DOCX)

Table S6 **Alleles ancestral in Neandertals and Denisovans, and derived in present-day humans.** Derived alleles are defined as being observed at high frequency (>=90%) in the 1000 Genomes project. Positions on chromosome 6 in the human genome (hg19), the ancestral, and the derived allele are shown. A region between the first transcription start site of RUNX2 +10,000 bases upstream, and the last transcription end site +10,000 bases has been considered. P2 = Promoter 2 of *RUNX2*, regulatory = regulatory element as defined in the ENSEMBL database.
(DOCX)

Table S7 **Culture conditions, transfection kits, transfection programs applied for each cell line.** Cell Line Nucleofector™ Kits: V (VVCA-1003), L (VCA-1005), T (VCA-1002), R (VCA-1001), Mammalian Fibroblast (VPI-1002). Invitrogen media and supplements: A-MEM (12492-013), F12 (21765-029), D-MEM (31966-047), McCoy’s 5A medium (22330-070), D-MEM/F12 (21041-033). Invitrogen supplements: Fetal bovine serum (FBS, 10270-106), 100x Glutamine (25030-123), Geneticin® (G418, 10131-027), 100x Penicillin/Streptomycin (P/S, 15070-063).
(DOCX)
