# Supplementary figures and images for: Identification of Putative Target Genes of the Transcription Factor RUNX2
Source: PLoS One. 2013 Dec 12;8(12):e83218. doi: 10.1371/journal.pone.0083218 (PMC3861491; doi:10.1371/journal.pone.0083218)

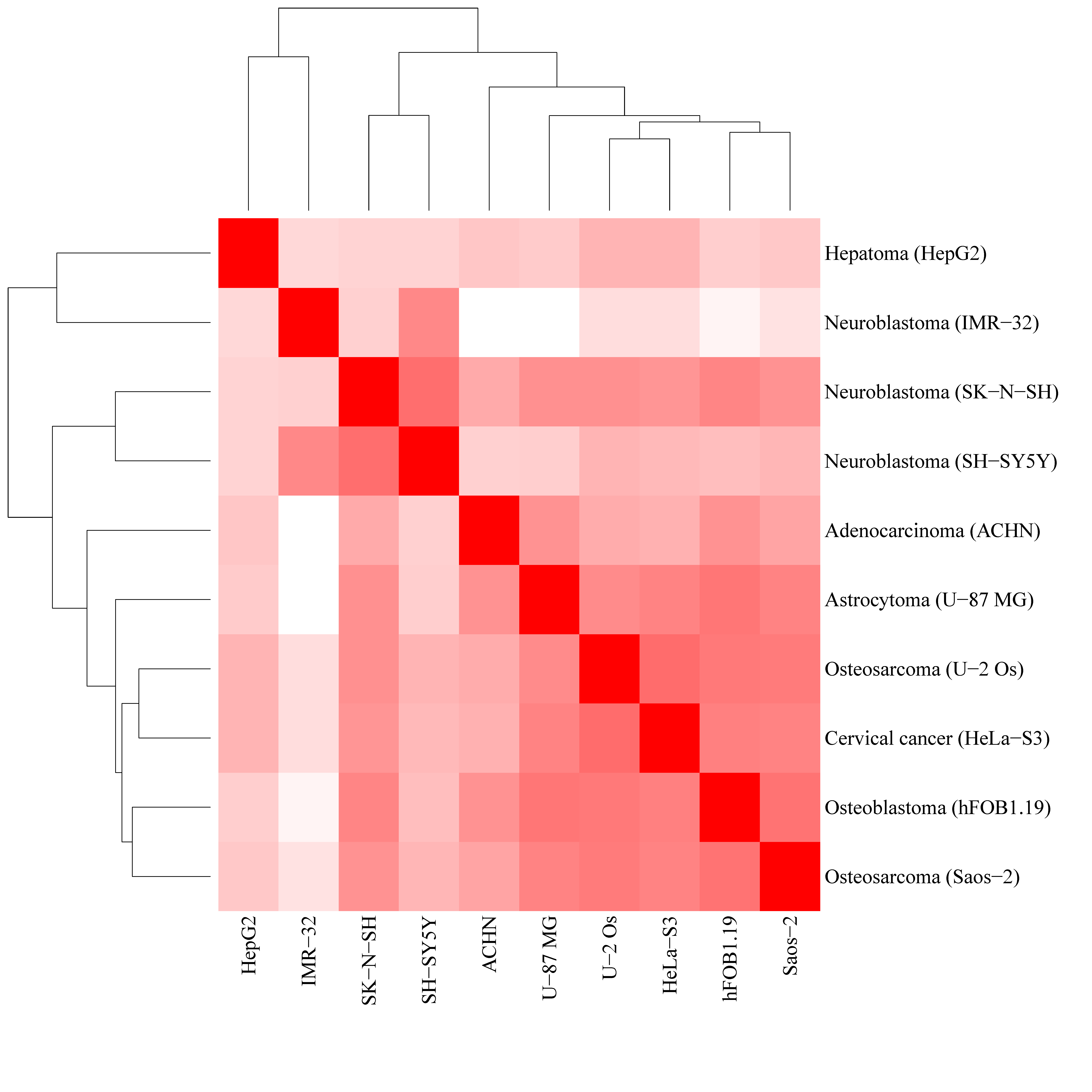

Supplement: Figure S1 — Pairwise distance matrix of overall mean expression values in control transfections between cell lines. The hepatoma cell line differs the most from the other cell lines in terms of its response to RUNX2 overexpression. The osteoblastoma and one of the osteosarcoma cell lines are clustering with each other. (TIF) [file pone.0083218.s001.tif]

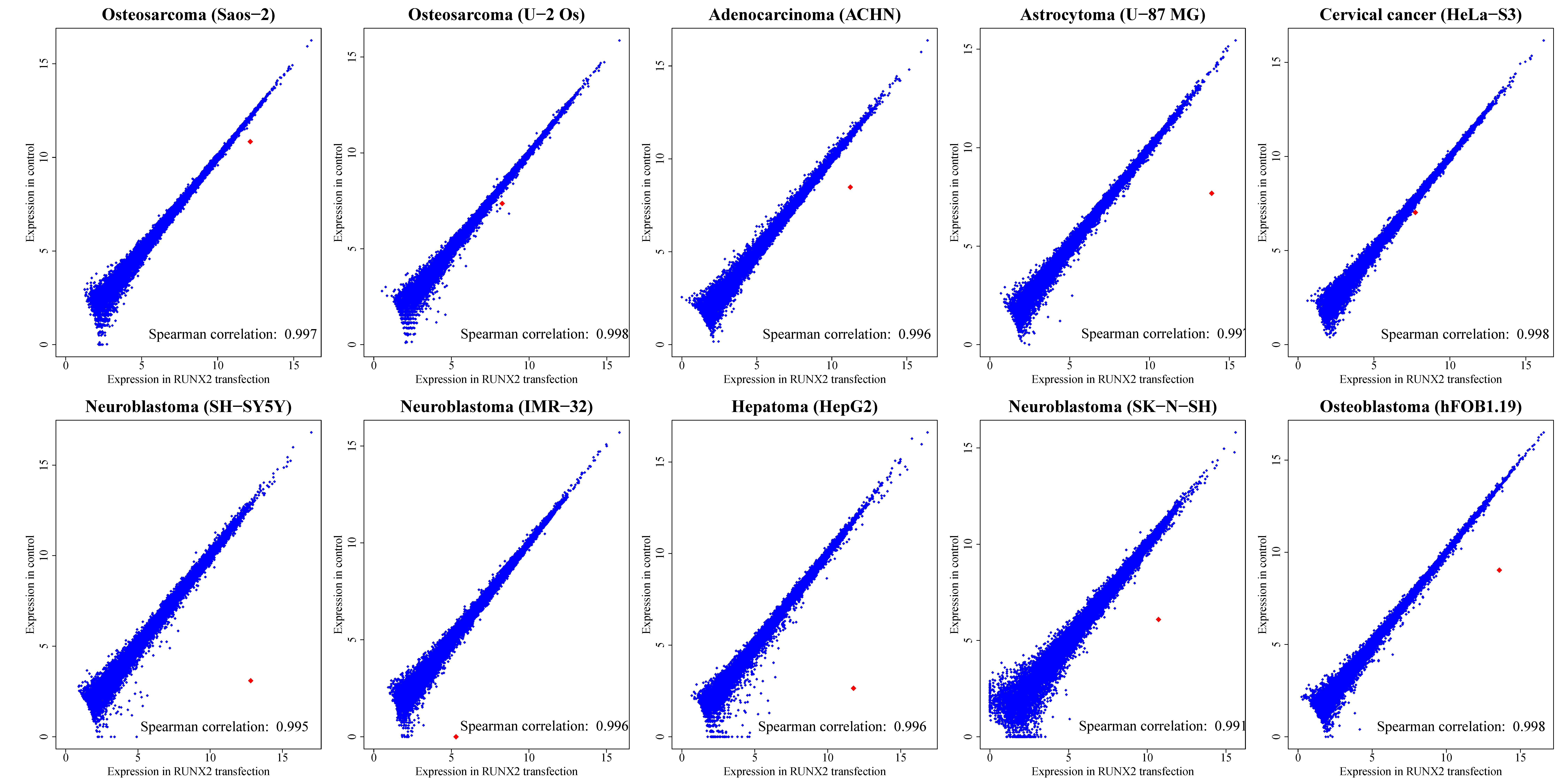

Supplement: Figure S3 — Correlations of expression within cell lines. Mean expression of genes after RUNX2 overexpression and mean expression of genes in control transfection. Expression of RUNX2 is given in red. Values are log2-transformed. Spearman correlations are above 0.99. (TIF) [file pone.0083218.s003.tif]
