# Supplementary material for: Identification of Putative Target Genes of the Transcription Factor RUNX2
Source: PLoS One. 2013 Dec 12;8(12):e83218. doi: 10.1371/journal.pone.0083218 (PMC3861491; doi:10.1371/journal.pone.0083218)
Supplement: Table S1 — Expression of RUNX2 in transfected and mock transfected cell lines and number of differentially expressed genes. Average expression levels in raw count values are normalized to the relative library sizes using DESeq. (DOCX) [file pone.0083218.s004.docx]

**Table S1.** Expression of RUNX2 in transfected and mock transfected cell lines and number of differentially expressed genes. Average expression levels in raw count values are normalized to the relative library sizes using *DESeq*.

| **Cell line** | **Mock transfection** | ***RUNX2* transfection** | **Fold-change** | **Number of differentially expressed genes** |
| --- | --- | --- | --- | --- |
| **ACHN** | 356 | 2406 | 6.76 | 448 |
| **HeLa-S3** | 130 | 211 | 1.62 | 38 |
| **HepG2** | 6 | 3493 | NA | 493 |
| **hFOB1.19** | 522 | 12201 | 23.37 | 89 |
| **IMR-32** | 0 | 39 | NA | 54 |
| **Saos-2** | 1833 | 4524 | 2.47 | 5 |
| **SH-SY5Y** | 9 | 7162 | NA | 691 |
| **SK-N-SH** | 68 | 1677 | 24.63 | 42 |
| **U-2 Os** | 164 | 308 | 1.87 | 42 |
| **U-87 MG** | 206 | 15259 | 74.19 | 205 |
