# Supplementary material for: Identification of Putative Target Genes of the Transcription Factor RUNX2
Source: PLoS One. 2013 Dec 12;8(12):e83218. doi: 10.1371/journal.pone.0083218 (PMC3861491; doi:10.1371/journal.pone.0083218)
Supplement: Table S2 — The 1,715 genes found to be differentially expressed after RUNX2 transfection in 10 cell lines. Genes are sorted by the number of cell lines in which they were found to be differentially expressed. (DOCX) [file pone.0083218.s005.docx]

**Table S2**. The 1,715 genes found to be differentially expressed after *RUNX2* transfection in 10 cell lines, sorted by the number of cell lines in which they were found to be differentially expressed.

| **Gene name** | **Cell lines** | **Ensembl ID** | **Description** |
| --- | --- | --- | --- |
| **BIRC3** | ACHN; HeLa-S3; HepG2; hFOB1.19; SH-SY5Y; U-2 Os; U-87 MG | ENSG00000023445 | baculoviral IAP repeat containing 3 |
| **ATF3** | ACHN; HeLa-S3; HepG2; SH-SY5Y; SK-N-SH | ENSG00000162772 | activating transcription factor 3 |
| **EGR1** | ACHN; hFOB1.19; SH-SY5Y; SK-N-SH; U-87 MG | ENSG00000120738 | early growth response 1 |
| **MALAT1** | HeLa-S3; HepG2; IMR-32; Saos-2; SH-SY5Y | ENSG00000251562 | metastasis associated lung adenocarcinoma transcript 1 (non-protein coding) |
| **MT-ATP6** | ACHN; HepG2; SH-SY5Y; U-2 Os; U-87 MG | ENSG00000198899 | mitochondrially encoded ATP synthase 6 |
| **MT-CYB** | ACHN; HepG2; IMR-32; SH-SY5Y; U-87 MG | ENSG00000198727 | mitochondrially encoded cytochrome b |
| **MTATP6P1** | ACHN; HepG2; SH-SY5Y; U-2 Os; U-87 MG | ENSG00000248527 | mitochondrially encoded ATP synthase 6 pseudogene 1 |
| **NEAT1** | ACHN; HeLa-S3; hFOB1.19; SH-SY5Y; U-87 MG | ENSG00000245532 | nuclear paraspeckle assembly transcript 1 (non-protein coding) |
| **AHNAK** | HeLa-S3; hFOB1.19; IMR-32; U-87 MG | ENSG00000124942 | AHNAK nucleoprotein |
| **CD82** | HepG2; hFOB1.19; SH-SY5Y; U-2 Os | ENSG00000085117 | CD82 molecule |
| **DUSP10** | hFOB1.19; SH-SY5Y; SK-N-SH; U-87 MG | ENSG00000143507 | dual specificity phosphatase 10 |
| **HMOX1** | ACHN; IMR-32; SH-SY5Y; SK-N-SH | ENSG00000100292 | heme oxygenase (decycling) 1 |
| **IL11** | HepG2; hFOB1.19; SH-SY5Y; U-87 MG | ENSG00000095752 | interleukin 11 |
| **KLF9** | HepG2; hFOB1.19; SH-SY5Y; U-87 MG | ENSG00000119138 | Kruppel-like factor 9 |
| **LRP1** | HepG2; IMR-32; SH-SY5Y; U-2 Os | ENSG00000123384 | low density lipoprotein receptor-related protein 1 |
| **MEGF10** | hFOB1.19; SH-SY5Y; SK-N-SH; U-2 Os | ENSG00000145794 | multiple EGF-like-domains 10 |
| **MT-CO3** | ACHN; HepG2; SH-SY5Y; U-87 MG | ENSG00000198938 | mitochondrially encoded cytochrome c oxidase III |
| **MT-ND1** | ACHN; HepG2; SH-SY5Y; U-87 MG | ENSG00000198888 | mitochondrially encoded NADH dehydrogenase 1 |
| **MT-ND4L** | ACHN; HepG2; IMR-32; SH-SY5Y | ENSG00000212907 | mitochondrially encoded NADH dehydrogenase 4L |
| **MT-ND5** | ACHN; HepG2; IMR-32; U-87 MG | ENSG00000198786 | mitochondrially encoded NADH dehydrogenase 5 |
| **MT-ND6** | ACHN; HepG2; IMR-32; U-87 MG | ENSG00000198695 | mitochondrially encoded NADH dehydrogenase 6 |
| **NFKBIE** | ACHN; IMR-32; SH-SY5Y; SK-N-SH | ENSG00000146232 | nuclear factor of kappa light polypeptide gene enhancer in B-cells inhibitor, epsilon |
| **PPP1R15A** | HeLa-S3; HepG2; SH-SY5Y; U-87 MG | ENSG00000087074 | protein phosphatase 1, regulatory subunit 15A |
| **UNC5B** | hFOB1.19; IMR-32; U-2 Os; U-87 MG | ENSG00000107731 | unc-5 homolog B (*C. elegans*) |
| **AMIGO2** | ACHN; hFOB1.19; U-87 MG | ENSG00000139211 | adhesion molecule with Ig-like domain 2 |
| **ARHGEF2** | HepG2; SH-SY5Y; U-87 MG | ENSG00000116584 | Rho/Rac guanine nucleotide exchange factor (GEF) 2 |
| **ASF1B** | HepG2; SH-SY5Y; U-87 MG | ENSG00000105011 | ASF1 anti-silencing function 1 homolog B (*S. cerevisiae*) |
| **BHLHE40** | ACHN; HepG2; SH-SY5Y | ENSG00000134107 | basic helix-loop-helix family, member e40 |
| **COL12A1** | ACHN; HepG2; U-87 MG | ENSG00000111799 | collagen, type XII, alpha 1 |
| **COL1A1** | HepG2; IMR-32; SH-SY5Y | ENSG00000108821 | collagen, type I, alpha 1 |
| **COL7A1** | ACHN; HepG2; hFOB1.19 | ENSG00000114270 | collagen, type VII, alpha 1 |
| **DRAXIN** | HepG2; SH-SY5Y; U-2 Os | ENSG00000162490 | dorsal inhibitory axon guidance protein |
| **E2F1** | HepG2; SH-SY5Y; U-87 MG | ENSG00000101412 | E2F transcription factor 1 |
| **FSCN1** | SH-SY5Y; U-2 Os; U-87 MG | ENSG00000075618 | fascin homolog 1, actin-bundling protein (Strongylocentrotus purpuratus) |
| **GADD45A** | ACHN; HepG2; SH-SY5Y | ENSG00000116717 | growth arrest and DNA-damage-inducible, alpha |
| **GADD45B** | ACHN; SH-SY5Y; SK-N-SH | ENSG00000099860 | growth arrest and DNA-damage-inducible, beta |
| **GFPT2** | HepG2; hFOB1.19; U-2 Os | ENSG00000131459 | glutamine-fructose-6-phosphate transaminase 2 |
| **GJB2** | ACHN; HepG2; U-87 MG | ENSG00000165474 | gap junction protein, beta 2, 26kDa |
| **HIVEP2** | ACHN; HepG2; U-87 MG | ENSG00000010818 | human immunodeficiency virus type I enhancer binding protein 2 |
| **ID3** | IMR-32; SH-SY5Y; U-87 MG | ENSG00000117318 | inhibitor of DNA binding 3, dominant negative helix-loop-helix protein |
| **IL23A** | HepG2; SK-N-SH; U-87 MG | ENSG00000110944 | interleukin 23, alpha subunit p19 |
| **ITGA2** | ACHN; HeLa-S3; U-87 MG | ENSG00000164171 | integrin, alpha 2 (CD49B, alpha 2 subunit of VLA-2 receptor) |
| **JUNB** | ACHN; HepG2; SH-SY5Y | ENSG00000171223 | jun B proto-oncogene |
| **KLF10** | ACHN; HepG2; SH-SY5Y | ENSG00000155090 | Kruppel-like factor 10 |
| **LACC1** | hFOB1.19; SH-SY5Y; U-87 MG | ENSG00000179630 | laccase (multicopper oxidoreductase) domain containing 1 |
| **LIG1** | ACHN; HepG2; SH-SY5Y | ENSG00000105486 | ligase I, DNA, ATP-dependent |
| **MMP13** | SH-SY5Y; SK-N-SH; U-2 Os | ENSG00000137745 | matrix metallopeptidase 13 (collagenase 3) |
| **MMP9** | hFOB1.19; SH-SY5Y; U-2 Os | ENSG00000100985 | matrix metallopeptidase 9 (gelatinase B, 92kDa gelatinase, 92kDa type IV collagenase) |
| **MT-ATP8** | ACHN; HepG2; SH-SY5Y | ENSG00000228253 | mitochondrially encoded ATP synthase 8 |
| **MT-CO1** | ACHN; HepG2; U-87 MG | ENSG00000198804 | mitochondrially encoded cytochrome c oxidase I |
| **MT-ND4** | ACHN; HepG2; SH-SY5Y | ENSG00000198886 | mitochondrially encoded NADH dehydrogenase 4 |
| **MTND2P28** | ACHN; HepG2; SH-SY5Y | ENSG00000225630 | MT-ND2 pseudogene 28 |
| **MYBL2** | HepG2; SH-SY5Y; U-87 MG | ENSG00000101057 | v-myb myeloblastosis viral oncogene homolog (avian)-like 2 |
| **NGFR** | HepG2; hFOB1.19; SH-SY5Y | ENSG00000064300 | nerve growth factor receptor |
| **PLAU** | ACHN; hFOB1.19; SK-N-SH | ENSG00000122861 | plasminogen activator, urokinase |
| **PLXNA4** | IMR-32; SH-SY5Y; U-2 Os | ENSG00000221866 | plexin A4 |
| **PMAIP1** | HepG2; SH-SY5Y; U-87 MG | ENSG00000141682 | phorbol-12-myristate-13-acetate-induced protein 1 |
| **RELB** | ACHN; HepG2; SH-SY5Y | ENSG00000104856 | v-rel reticuloendotheliosis viral oncogene homolog B |
| **SLC16A6** | HepG2; hFOB1.19; U-87 MG | ENSG00000108932 | solute carrier family 16, member 6 (monocarboxylic acid transporter 7) |
| **SLC7A11** | Saos-2; SH-SY5Y; U-87 MG | ENSG00000151012 | solute carrier family 7 (anionic amino acid transporter light chain, xc- system), member 11 |
| **SLFN5** | ACHN; HepG2; U-87 MG | ENSG00000166750 | schlafen family member 5 |
| **SPOCK1** | HepG2; IMR-32; SH-SY5Y | ENSG00000152377 | sparc/osteonectin, cwcv and kazal-like domains proteoglycan (testican) 1 |
| **SYNE2** | hFOB1.19; IMR-32; SH-SY5Y | ENSG00000054654 | spectrin repeat containing, nuclear envelope 2 |
| **TAF1D** | ACHN; SH-SY5Y; SK-N-SH | ENSG00000166012 | TATA box binding protein (TBP)-associated factor, RNA polymerase I, D, 41kDa |
| **TRAF1** | ACHN; HepG2; SH-SY5Y | ENSG00000056558 | TNF receptor-associated factor 1 |
| **UBR4** | HeLa-S3; IMR-32; U-87 MG | ENSG00000127481 | ubiquitin protein ligase E3 component n-recognin 4 |
| **WNT5A** | hFOB1.19; SK-N-SH; U-87 MG | ENSG00000114251 | wingless-type MMTV integration site family, member 5A |
| **YPEL5** | HepG2; SH-SY5Y; U-87 MG | ENSG00000119801 | yippee-like 5 (Drosophila) |
| **ABL2** | HepG2; U-87 MG | ENSG00000143322 | v-abl Abelson murine leukemia viral oncogene homolog 2 |
| **AC020571.3** | HepG2; hFOB1.19 | ENSG00000229056 | NA |
| **ACTBP2** | HepG2; U-87 MG | ENSG00000213763 | actin, beta pseudogene 2 |
| **ADAMTS5** | hFOB1.19; U-87 MG | ENSG00000154736 | ADAM metallopeptidase with thrombospondin type 1 motif, 5 |
| **AEN** | ACHN; SH-SY5Y | ENSG00000181026 | apoptosis enhancing nuclease |
| **AGPAT9** | HeLa-S3; HepG2 | ENSG00000138678 | 1-acylglycerol-3-phosphate O-acyltransferase 9 |
| **AHNAK2** | hFOB1.19; SH-SY5Y | ENSG00000185567 | AHNAK nucleoprotein 2 |
| **AIM1** | HepG2; SH-SY5Y | ENSG00000112297 | absent in melanoma 1 |
| **AKAP8L** | ACHN; SH-SY5Y | ENSG00000011243 | A kinase (PRKA) anchor protein 8-like |
| **ALPK2** | HepG2; U-87 MG | ENSG00000198796 | alpha-kinase 2 |
| **ANGPTL4** | SH-SY5Y; U-87 MG | ENSG00000167772 | angiopoietin-like 4 |
| **ANKRD28** | SH-SY5Y; U-87 MG | ENSG00000206560 | ankyrin repeat domain 28 |
| **ARC** | ACHN; SK-N-SH | ENSG00000198576 | activity-regulated cytoskeleton-associated protein |
| **ASB2** | HepG2; SH-SY5Y | ENSG00000100628 | ankyrin repeat and SOCS box containing 2 |
| **ATF4** | SH-SY5Y; U-87 MG | ENSG00000128272 | activating transcription factor 4 (tax-responsive enhancer element B67) |
| **ATOH8** | Saos-2; U-87 MG | ENSG00000168874 | atonal homolog 8 (Drosophila) |
| **ATP6V0D2** | U-2 Os; U-87 MG | ENSG00000147614 | ATPase, H+ transporting, lysosomal 38kDa, V0 subunit d2 |
| **ATP6V0E2** | HepG2; SH-SY5Y | ENSG00000171130 | ATPase, H+ transporting V0 subunit e2 |
| **AXL** | HepG2; SH-SY5Y | ENSG00000167601 | AXL receptor tyrosine kinase |
| **BAZ2B** | ACHN; HepG2 | ENSG00000123636 | bromodomain adjacent to zinc finger domain, 2B |
| **BDKRB2** | HepG2; SH-SY5Y | ENSG00000168398 | bradykinin receptor B2 |
| **BHLHE41** | ACHN; SK-N-SH | ENSG00000123095 | basic helix-loop-helix family, member e41 |
| **C10orf2** | SH-SY5Y; U-87 MG | ENSG00000107815 | chromosome 10 open reading frame 2 |
| **C4orf26** | U-2 Os; U-87 MG | ENSG00000174792 | chromosome 4 open reading frame 26 |
| **CARS** | HepG2; SH-SY5Y | ENSG00000110619 | cysteinyl-tRNA synthetase |
| **CCNA2** | HepG2; SH-SY5Y | ENSG00000145386 | cyclin A2 |
| **CCNL1** | ACHN; SH-SY5Y | ENSG00000163660 | cyclin L1 |
| **CCRN4L** | ACHN; SK-N-SH | ENSG00000151014 | CCR4 carbon catabolite repression 4-like (*S. cerevisiae*) |
| **CD44** | HepG2; SH-SY5Y | ENSG00000026508 | CD44 molecule (Indian blood group) |
| **CDC42EP3** | hFOB1.19; U-87 MG | ENSG00000163171 | CDC42 effector protein (Rho GTPase binding) 3 |
| **CDC6** | HepG2; SH-SY5Y | ENSG00000094804 | cell division cycle 6 |
| **CDCA5** | HepG2; SH-SY5Y | ENSG00000146670 | cell division cycle associated 5 |
| **CDCP1** | HeLa-S3; HepG2 | ENSG00000163814 | CUB domain containing protein 1 |
| **CDKN2C** | ACHN; HepG2 | ENSG00000123080 | cyclin-dependent kinase inhibitor 2C (p18, inhibits CDK4) |
| **CDT1** | HepG2; SH-SY5Y | ENSG00000167513 | chromatin licensing and DNA replication factor 1 |
| **CEBPB** | ACHN; SH-SY5Y | ENSG00000172216 | CCAAT/enhancer binding protein (C/EBP), beta |
| **CLDN1** | HeLa-S3; HepG2 | ENSG00000163347 | claudin 1 |
| **COL11A2** | ACHN; HepG2 | ENSG00000204248 | collagen, type XI, alpha 2 |
| **COL6A3** | SK-N-SH; U-2 Os | ENSG00000163359 | collagen, type VI, alpha 3 |
| **CPA4** | HeLa-S3; HepG2 | ENSG00000128510 | carboxypeptidase A4 |
| **CRABP2** | HepG2; IMR-32 | ENSG00000143320 | cellular retinoic acid binding protein 2 |
| **CSRNP1** | ACHN; SK-N-SH | ENSG00000144655 | cysteine-serine-rich nuclear protein 1 |
| **CTD-2021J15.2** | HepG2; U-87 MG | ENSG00000240476 | NA |
| **CTGF** | U-2 Os; U-87 MG | ENSG00000118523 | connective tissue growth factor |
| **CTH** | HepG2; U-87 MG | ENSG00000116761 | cystathionase (cystathionine gamma-lyase) |
| **DCBLD2** | HepG2; SH-SY5Y | ENSG00000057019 | discoidin, CUB and LCCL domain containing 2 |
| **DDC** | HepG2; SH-SY5Y | ENSG00000132437 | dopa decarboxylase (aromatic L-amino acid decarboxylase) |
| **DDIT3** | HepG2; SH-SY5Y | ENSG00000175197 | DNA-damage-inducible transcript 3 |
| **DKK1** | HepG2; IMR-32 | ENSG00000107984 | dickkopf 1 homolog (*Xenopus laevis*) |
| **DLK1** | HepG2; SH-SY5Y | ENSG00000185559 | delta-like 1 homolog (Drosophila) |
| **DMBT1** | HepG2; U-2 Os | ENSG00000187908 | deleted in malignant brain tumors 1 |
| **DNAH17** | ACHN; HepG2 | ENSG00000187775 | dynein, axonemal, heavy chain 17 |
| **DOCK9** | ACHN; HepG2 | ENSG00000088387 | dedicator of cytokinesis 9 |
| **DPYSL3** | HepG2; SH-SY5Y | ENSG00000113657 | dihydropyrimidinase-like 3 |
| **DSP** | HeLa-S3; hFOB1.19 | ENSG00000096696 | desmoplakin |
| **DST** | SH-SY5Y; U-87 MG | ENSG00000151914 | dystonin |
| **DUSP4** | IMR-32; SH-SY5Y | ENSG00000120875 | dual specificity phosphatase 4 |
| **DUSP5** | HepG2; SH-SY5Y | ENSG00000138166 | dual specificity phosphatase 5 |
| **DYNC1H1** | HeLa-S3; U-2 Os | ENSG00000197102 | dynein, cytoplasmic 1, heavy chain 1 |
| **EFNB2** | ACHN; SH-SY5Y | ENSG00000125266 | ephrin-B2 |
| **EGR4** | ACHN; SH-SY5Y | ENSG00000135625 | early growth response 4 |
| **EMP1** | HepG2; SH-SY5Y | ENSG00000134531 | epithelial membrane protein 1 |
| **ENPP1** | HepG2; U-2 Os | ENSG00000197594 | ectonucleotide pyrophosphatase/phosphodiesterase 1 |
| **ERBB3** | HepG2; hFOB1.19 | ENSG00000065361 | v-erb-b2 erythroblastic leukemia viral oncogene homolog 3 (avian) |
| **ESPL1** | HepG2; SH-SY5Y | ENSG00000135476 | extra spindle pole bodies homolog 1 (*S. cerevisiae*) |
| **F3** | HeLa-S3; U-87 MG | ENSG00000117525 | coagulation factor III (thromboplastin, tissue factor) |
| **FAM49A** | ACHN; U-2 Os | ENSG00000197872 | family with sequence similarity 49, member A |
| **FAM53C** | ACHN; SH-SY5Y | ENSG00000120709 | family with sequence similarity 53, member C |
| **FANCI** | HepG2; SH-SY5Y | ENSG00000140525 | Fanconi anemia, complementation group I |
| **FAT1** | HeLa-S3; U-87 MG | ENSG00000083857 | FAT tumor suppressor homolog 1 (Drosophila) |
| **FAT3** | SH-SY5Y; U-2 Os | ENSG00000165323 | FAT tumor suppressor homolog 3 (Drosophila) |
| **FBN1** | SH-SY5Y; SK-N-SH | ENSG00000166147 | fibrillin 1 |
| **FILIP1L** | ACHN; SK-N-SH | ENSG00000168386 | filamin A interacting protein 1-like |
| **FMNL1** | HepG2; U-2 Os | ENSG00000184922 | formin-like 1 |
| **FOSL1** | HeLa-S3; SH-SY5Y | ENSG00000175592 | FOS-like antigen 1 |
| **FOSL2** | HepG2; SH-SY5Y | ENSG00000075426 | FOS-like antigen 2 |
| **FOXM1** | HepG2; SH-SY5Y | ENSG00000111206 | forkhead box M1 |
| **GABARAPL1** | SH-SY5Y; U-87 MG | ENSG00000139112 | GABA(A) receptor-associated protein like 1 |
| **GDF15** | SH-SY5Y; U-87 MG | ENSG00000130513 | growth differentiation factor 15 |
| **GLRX** | ACHN; HepG2 | ENSG00000173221 | glutaredoxin (thioltransferase) |
| **GPR84** | HepG2; U-87 MG | ENSG00000139572 | G protein-coupled receptor 84 |
| **GPRC5C** | ACHN; HepG2 | ENSG00000170412 | G protein-coupled receptor, family C, group 5, member C |
| **GTSE1** | HepG2; SH-SY5Y | ENSG00000075218 | G-2 and S-phase expressed 1 |
| **HBEGF** | HepG2; hFOB1.19 | ENSG00000113070 | heparin-binding EGF-like growth factor |
| **HIST1H1C** | HepG2; SK-N-SH | ENSG00000187837 | histone cluster 1, H1c |
| **HLF** | ACHN; HepG2 | ENSG00000108924 | hepatic leukemia factor |
| **hsa-mir-6723** | HepG2; IMR-32 | ENSG00000237973 | hsa-mir-6723 |
| **ICAM1** | ACHN; hFOB1.19 | ENSG00000090339 | intercellular adhesion molecule 1 |
| **IER2** | ACHN; SH-SY5Y | ENSG00000160888 | immediate early response 2 |
| **IGF2** | hFOB1.19; SH-SY5Y | ENSG00000167244 | insulin-like growth factor 2 (somatomedin A) |
| **IGFBP3** | hFOB1.19; SH-SY5Y | ENSG00000146674 | insulin-like growth factor binding protein 3 |
| **IL1A** | hFOB1.19; U-87 MG | ENSG00000115008 | interleukin 1, alpha |
| **IL1R1** | hFOB1.19; U-87 MG | ENSG00000115594 | interleukin 1 receptor, type I |
| **IL6** | ACHN; hFOB1.19 | ENSG00000136244 | interleukin 6 (interferon, beta 2) |
| **IL8** | ACHN; U-87 MG | ENSG00000169429 | interleukin 8 |
| **INCENP** | HepG2; SH-SY5Y | ENSG00000149503 | inner centromere protein antigens 135/155kDa |
| **IRF1** | SH-SY5Y; U-87 MG | ENSG00000125347 | interferon regulatory factor 1 |
| **ITGAM** | ACHN; HepG2 | ENSG00000169896 | integrin, alpha M (complement component 3 receptor 3 subunit) |
| **ITPKC** | ACHN; SH-SY5Y | ENSG00000086544 | inositol-trisphosphate 3-kinase C |
| **JMY** | ACHN; U-87 MG | ENSG00000152409 | junction mediating and regulatory protein, p53 cofactor |
| **KALRN** | HepG2; SH-SY5Y | ENSG00000160145 | kalirin, RhoGEF kinase |
| **KIAA0101** | HepG2; SH-SY5Y | ENSG00000166803 | KIAA0101 |
| **KIAA1217** | U-2 Os; U-87 MG | ENSG00000120549 | KIAA1217 |
| **KIF18B** | HepG2; SH-SY5Y | ENSG00000186185 | kinesin family member 18B |
| **KIF20A** | HepG2; SH-SY5Y | ENSG00000112984 | kinesin family member 20A |
| **KIF26B** | SH-SY5Y; U-87 MG | ENSG00000162849 | kinesin family member 26B |
| **KLF5** | ACHN; U-87 MG | ENSG00000102554 | Kruppel-like factor 5 (intestinal) |
| **KLF6** | HeLa-S3; SH-SY5Y | ENSG00000067082 | Kruppel-like factor 6 |
| **LAMA5** | HepG2; SH-SY5Y | ENSG00000130702 | laminin, alpha 5 |
| **LAMB3** | ACHN; HepG2 | ENSG00000196878 | laminin, beta 3 |
| **LMNB1** | HepG2; SH-SY5Y | ENSG00000113368 | lamin B1 |
| **LMO7** | ACHN; SK-N-SH | ENSG00000136153 | LIM domain 7 |
| **LPAR6** | ACHN; HepG2 | ENSG00000139679 | lysophosphatidic acid receptor 6 |
| **LUM** | hFOB1.19; U-87 MG | ENSG00000139329 | lumican |
| **MACF1** | HeLa-S3; SH-SY5Y | ENSG00000127603 | microtubule-actin crosslinking factor 1 |
| **MAFF** | ACHN; HepG2 | ENSG00000185022 | v-maf musculoaponeurotic fibrosarcoma oncogene homolog F (avian) |
| **MAP3K14** | ACHN; SK-N-SH | ENSG00000006062 | mitogen-activated protein kinase kinase kinase 14 |
| **MCM2** | HepG2; SH-SY5Y | ENSG00000073111 | minichromosome maintenance complex component 2 |
| **MCM3** | HepG2; SH-SY5Y | ENSG00000112118 | minichromosome maintenance complex component 3 |
| **MCM5** | HepG2; SH-SY5Y | ENSG00000100297 | minichromosome maintenance complex component 5 |
| **MKI67** | HepG2; SH-SY5Y | ENSG00000148773 | antigen identified by monoclonal antibody Ki-67 |
| **MLL2** | IMR-32; SH-SY5Y | ENSG00000167548 | myeloid/lymphoid or mixed-lineage leukemia 2 |
| **MMP14** | HepG2; hFOB1.19 | ENSG00000157227 | matrix metallopeptidase 14 (membrane-inserted) |
| **MT-CO2** | SH-SY5Y; U-87 MG | ENSG00000198712 | mitochondrially encoded cytochrome c oxidase II |
| **MT-ND2** | HepG2; SH-SY5Y | ENSG00000198763 | mitochondrially encoded NADH dehydrogenase 2 |
| **MT2A** | HepG2; hFOB1.19 | ENSG00000125148 | metallothionein 2A |
| **MXD1** | HepG2; SH-SY5Y | ENSG00000059728 | MAX dimerization protein 1 |
| **MYBL1** | HeLa-S3; U-87 MG | ENSG00000185697 | v-myb myeloblastosis viral oncogene homolog (avian)-like 1 |
| **MYNN** | ACHN; SH-SY5Y | ENSG00000085274 | myoneurin |
| **NAV1** | HepG2; IMR-32 | ENSG00000134369 | neuron navigator 1 |
| **NAV2** | hFOB1.19; SH-SY5Y | ENSG00000166833 | neuron navigator 2 |
| **NCAPD2** | HepG2; SH-SY5Y | ENSG00000010292 | non-SMC condensin I complex, subunit D2 |
| **NCAPH** | HepG2; SH-SY5Y | ENSG00000121152 | non-SMC condensin I complex, subunit H |
| **NFAT5** | ACHN; U-87 MG | ENSG00000102908 | nuclear factor of activated T-cells 5, tonicity-responsive |
| **NFKBIB** | ACHN; SH-SY5Y | ENSG00000104825 | nuclear factor of kappa light polypeptide gene enhancer in B-cells inhibitor, beta |
| **NQO1** | ACHN; SH-SY5Y | ENSG00000181019 | NAD(P)H dehydrogenase, quinone 1 |
| **NR1D1** | ACHN; SK-N-SH | ENSG00000126368 | nuclear receptor subfamily 1, group D, member 1 |
| **NR1D2** | ACHN; U-87 MG | ENSG00000174738 | nuclear receptor subfamily 1, group D, member 2 |
| **NRG1** | hFOB1.19; U-87 MG | ENSG00000157168 | neuregulin 1 |
| **NT5E** | ACHN; SH-SY5Y | ENSG00000135318 | 5'-nucleotidase, ecto (CD73) |
| **NUAK2** | ACHN; SK-N-SH | ENSG00000163545 | NUAK family, SNF1-like kinase, 2 |
| **OSBPL3** | HepG2; SH-SY5Y | ENSG00000070882 | oxysterol binding protein-like 3 |
| **PDGFA** | HepG2; SH-SY5Y | ENSG00000197461 | platelet-derived growth factor alpha polypeptide |
| **PDLIM4** | HepG2; SH-SY5Y | ENSG00000131435 | PDZ and LIM domain 4 |
| **PER1** | ACHN; SH-SY5Y | ENSG00000179094 | period circadian clock 1 |
| **PHLDA1** | HeLa-S3; U-87 MG | ENSG00000139289 | pleckstrin homology-like domain, family A, member 1 |
| **PHLDA3** | ACHN; SH-SY5Y | ENSG00000174307 | pleckstrin homology-like domain, family A, member 3 |
| **PKMYT1** | HepG2; SH-SY5Y | ENSG00000127564 | protein kinase, membrane associated tyrosine/threonine 1 |
| **PLA2G4C** | ACHN; HepG2 | ENSG00000105499 | phospholipase A2, group IVC (cytosolic, calcium-independent) |
| **PLAUR** | HepG2; hFOB1.19 | ENSG00000011422 | plasminogen activator, urokinase receptor |
| **PLK1** | HepG2; SH-SY5Y | ENSG00000166851 | polo-like kinase 1 |
| **PLK2** | HepG2; SH-SY5Y | ENSG00000145632 | polo-like kinase 2 |
| **PLK3** | ACHN; SH-SY5Y | ENSG00000173846 | polo-like kinase 3 |
| **PNPLA3** | hFOB1.19; U-87 MG | ENSG00000100344 | patatin-like phospholipase domain containing 3 |
| **POLE** | HepG2; SH-SY5Y | ENSG00000177084 | polymerase (DNA directed), epsilon, catalytic subunit |
| **PPM1D** | ACHN; SH-SY5Y | ENSG00000170836 | protein phosphatase, Mg2+/Mn2+ dependent, 1D |
| **PRKAB1** | ACHN; SH-SY5Y | ENSG00000111725 | protein kinase, AMP-activated, beta 1 non-catalytic subunit |
| **PTPRJ** | ACHN; HepG2 | ENSG00000149177 | protein tyrosine phosphatase, receptor type, J |
| **RELN** | HepG2; SH-SY5Y | ENSG00000189056 | reelin |
| **RIOK3** | HepG2; SH-SY5Y | ENSG00000101782 | RIO kinase 3 |
| **RND3** | SH-SY5Y; U-87 MG | ENSG00000115963 | Rho family GTPase 3 |
| **RNF213** | hFOB1.19; SH-SY5Y | ENSG00000173821 | ring finger protein 213 |
| **ROBO4** | hFOB1.19; SH-SY5Y | ENSG00000154133 | roundabout, axon guidance receptor, homolog 4 (Drosophila) |
| **RP11-47I22.3** | HepG2; U-2 Os | ENSG00000232774 | Uncharacterized protein |
| **RRAD** | ACHN; HepG2 | ENSG00000166592 | Ras-related associated with diabetes |
| **RRM2** | HepG2; SH-SY5Y | ENSG00000171848 | ribonucleotide reductase M2 |
| **RSRC2** | ACHN; SH-SY5Y | ENSG00000111011 | arginine/serine-rich coiled-coil 2 |
| **RTL1** | SH-SY5Y; U-2 Os | ENSG00000254656 | retrotransposon-like 1 |
| **SAPCD2** | HepG2; SH-SY5Y | ENSG00000186193 | suppressor APC domain containing 2 |
| **SDK2** | IMR-32; SH-SY5Y | ENSG00000069188 | sidekick cell adhesion molecule 2 |
| **SEL1L3** | ACHN; SH-SY5Y | ENSG00000091490 | sel-1 suppressor of lin-12-like 3 (*C. elegans*) |
| **SEMA7A** | hFOB1.19; SK-N-SH | ENSG00000138623 | semaphorin 7A, GPI membrane anchor (John Milton Hagen blood group) |
| **SERPINA6** | ACHN; HepG2 | ENSG00000170099 | serpin peptidase inhibitor, clade A (alpha-1 antiproteinase, antitrypsin), member 6 |
| **SERPINB1** | HepG2; hFOB1.19 | ENSG00000021355 | serpin peptidase inhibitor, clade B (ovalbumin), member 1 |
| **SESN2** | HepG2; SH-SY5Y | ENSG00000130766 | sestrin 2 |
| **SHROOM2** | hFOB1.19; U-87 MG | ENSG00000146950 | shroom family member 2 |
| **SHROOM3** | ACHN; HepG2 | ENSG00000138771 | shroom family member 3 |
| **SLC2A12** | HeLa-S3; HepG2 | ENSG00000146411 | solute carrier family 2 (facilitated glucose transporter), member 12 |
| **SLC3A2** | ACHN; SH-SY5Y | ENSG00000168003 | solute carrier family 3 (activators of dibasic and neutral amino acid transport), member 2 |
| **SMOX** | hFOB1.19; U-87 MG | ENSG00000088826 | spermine oxidase |
| **SNAI2** | HepG2; SH-SY5Y | ENSG00000019549 | snail homolog 2 (Drosophila) |
| **SNAPC1** | ACHN; U-87 MG | ENSG00000023608 | small nuclear RNA activating complex, polypeptide 1, 43kDa |
| **SNHG1** | SH-SY5Y; U-87 MG | ENSG00000255717 | small nucleolar RNA host gene 1 (non-protein coding) |
| **SORL1** | HepG2; SH-SY5Y | ENSG00000137642 | sortilin-related receptor, L(DLR class) A repeats containing |
| **SPAG5** | HepG2; SH-SY5Y | ENSG00000076382 | sperm associated antigen 5 |
| **SPRY4** | IMR-32; SH-SY5Y | ENSG00000187678 | sprouty homolog 4 (Drosophila) |
| **STC2** | HeLa-S3; SH-SY5Y | ENSG00000113739 | stanniocalcin 2 |
| **STK17B** | HepG2; U-87 MG | ENSG00000081320 | serine/threonine kinase 17b |
| **STRA6** | IMR-32; U-2 Os | ENSG00000137868 | stimulated by retinoic acid 6 |
| **SVIL** | HepG2; SH-SY5Y | ENSG00000197321 | supervillin |
| **SYNE1** | ACHN; U-87 MG | ENSG00000131018 | spectrin repeat containing, nuclear envelope 1 |
| **TACC3** | HepG2; SH-SY5Y | ENSG00000013810 | transforming, acidic coiled-coil containing protein 3 |
| **TBC1D5** | SH-SY5Y; SK-N-SH | ENSG00000131374 | TBC1 domain family, member 5 |
| **TENM4** | IMR-32; SH-SY5Y | ENSG00000149256 | teneurin transmembrane protein 4 |
| **TGFB2** | HepG2; hFOB1.19 | ENSG00000092969 | transforming growth factor, beta 2 |
| **THBS1** | HeLa-S3; SK-N-SH | ENSG00000137801 | thrombospondin 1 |
| **TICRR** | HepG2; SH-SY5Y | ENSG00000140534 | TOPBP1-interacting checkpoint and replication regulator |
| **TIMELESS** | HepG2; SH-SY5Y | ENSG00000111602 | timeless circadian clock |
| **TIPARP** | ACHN; SH-SY5Y | ENSG00000163659 | TCDD-inducible poly(ADP-ribose) polymerase |
| **TK1** | HepG2; SH-SY5Y | ENSG00000167900 | thymidine kinase 1, soluble |
| **TMCC3** | ACHN; U-87 MG | ENSG00000057704 | transmembrane and coiled-coil domain family 3 |
| **TNFAIP3** | ACHN; SK-N-SH | ENSG00000118503 | tumor necrosis factor, alpha-induced protein 3 |
| **TNFRSF10D** | ACHN; HepG2 | ENSG00000173530 | tumor necrosis factor receptor superfamily, member 10d, decoy with truncated death domain |
| **TNS4** | HeLa-S3; HepG2 | ENSG00000131746 | tensin 4 |
| **TRIB3** | SH-SY5Y; U-87 MG | ENSG00000101255 | tribbles homolog 3 (Drosophila) |
| **TRPV2** | ACHN; HepG2 | ENSG00000187688 | transient receptor potential cation channel, subfamily V, member 2 |
| **TSC22D1** | HepG2; U-87 MG | ENSG00000102804 | TSC22 domain family, member 1 |
| **TSPYL2** | ACHN; SH-SY5Y | ENSG00000184205 | TSPY-like 2 |
| **TXNRD1** | ACHN; SH-SY5Y | ENSG00000198431 | thioredoxin reductase 1 |
| **UBASH3B** | HepG2; SH-SY5Y | ENSG00000154127 | ubiquitin associated and SH3 domain containing B |
| **ULBP1** | HepG2; SH-SY5Y | ENSG00000111981 | UL16 binding protein 1 |
| **USP36** | SH-SY5Y; U-87 MG | ENSG00000055483 | ubiquitin specific peptidase 36 |
| **VDR** | HepG2; SH-SY5Y | ENSG00000111424 | vitamin D (1,25- dihydroxyvitamin D3) receptor |
| **VEGFA** | HepG2; hFOB1.19 | ENSG00000112715 | vascular endothelial growth factor A |
| **VPS11** | ACHN; SH-SY5Y | ENSG00000160695 | vacuolar protein sorting 11 homolog (*S. cerevisiae*) |
| **VWA5B2** | HepG2; SH-SY5Y | ENSG00000145198 | von Willebrand factor A domain containing 5B2 |
| **WNK2** | HepG2; SH-SY5Y | ENSG00000165238 | WNK lysine deficient protein kinase 2 |
| **ZFYVE28** | HepG2; U-87 MG | ENSG00000159733 | zinc finger, FYVE domain containing 28 |
| **ZMIZ2** | ACHN; SH-SY5Y | ENSG00000122515 | zinc finger, MIZ-type containing 2 |
| **ZNF274** | ACHN; HepG2 | ENSG00000171606 | zinc finger protein 274 |
| **ZNF554** | ACHN; HepG2 | ENSG00000172006 | zinc finger protein 554 |
| **ZNF697** | ACHN; HepG2 | ENSG00000143067 | zinc finger protein 697 |
| **AAK1** | U-87 MG | ENSG00000115977 | AP2 associated kinase 1 |
| **AAK1** | SK-N-SH | ENSG00000188971 | Uncharacterized protein |
| **AASS** | ACHN | ENSG00000008311 | aminoadipate-semialdehyde synthase |
| **ABCA12** | ACHN | ENSG00000144452 | ATP-binding cassette, sub-family A (ABC1), member 12 |
| **ABCA5** | ACHN | ENSG00000154265 | ATP-binding cassette, sub-family A (ABC1), member 5 |
| **ABCB1** | HepG2 | ENSG00000085563 | ATP-binding cassette, sub-family B (MDR/TAP), member 1 |
| **ABHD2** | HepG2 | ENSG00000140526 | abhydrolase domain containing 2 |
| **ABTB2** | HepG2 | ENSG00000166016 | ankyrin repeat and BTB (POZ) domain containing 2 |
| **AC004381.6** | ACHN | ENSG00000005189 | Putative RNA exonuclease NEF-sp |
| **AC009362.2** | U-2 Os | ENSG00000233287 | NA |
| **AC058791.2** | SH-SY5Y | ENSG00000231721 | NA |
| **AC112721.1** | hFOB1.19 | ENSG00000222022 | Uncharacterized protein |
| **ACADSB** | HepG2 | ENSG00000196177 | acyl-CoA dehydrogenase, short/branched chain |
| **ACHE** | ACHN | ENSG00000087085 | acetylcholinesterase |
| **ACPP** | SH-SY5Y | ENSG00000014257 | acid phosphatase, prostate |
| **ACSL4** | HepG2 | ENSG00000068366 | acyl-CoA synthetase long-chain family member 4 |
| **ACTA1** | HepG2 | ENSG00000143632 | actin, alpha 1, skeletal muscle |
| **ACTBP12** | U-87 MG | ENSG00000233125 | actin, beta pseudogene 12 |
| **ACTBP8** | U-87 MG | ENSG00000220267 | actin, beta pseudogene 8 |
| **ACTG1P1** | hFOB1.19 | ENSG00000178631 | actin, gamma 1 pseudogene 1 |
| **ACTR1B** | ACHN | ENSG00000115073 | ARP1 actin-related protein 1 homolog B, centractin beta (yeast) |
| **ADAM12** | SH-SY5Y | ENSG00000148848 | ADAM metallopeptidase domain 12 |
| **ADAM22** | SH-SY5Y | ENSG00000008277 | ADAM metallopeptidase domain 22 |
| **ADAMTS15** | hFOB1.19 | ENSG00000166106 | ADAM metallopeptidase with thrombospondin type 1 motif, 15 |
| **ADAMTS7** | SH-SY5Y | ENSG00000136378 | ADAM metallopeptidase with thrombospondin type 1 motif, 7 |
| **ADARB1** | hFOB1.19 | ENSG00000197381 | adenosine deaminase, RNA-specific, B1 |
| **ADCK4** | ACHN | ENSG00000123815 | aarF domain containing kinase 4 |
| **ADCY1** | SH-SY5Y | ENSG00000164742 | adenylate cyclase 1 (brain) |
| **ADCYAP1R1** | SH-SY5Y | ENSG00000078549 | adenylate cyclase activating polypeptide 1 (pituitary) receptor type I |
| **ADD2** | SH-SY5Y | ENSG00000075340 | adducin 2 (beta) |
| **ADH6** | HepG2 | ENSG00000172955 | alcohol dehydrogenase 6 (class V) |
| **ADIRF** | ACHN | ENSG00000148671 | adipogenesis regulatory factor |
| **ADM** | U-87 MG | ENSG00000148926 | adrenomedullin |
| **ADORA1** | U-87 MG | ENSG00000163485 | adenosine A1 receptor |
| **ADRBK2** | SH-SY5Y | ENSG00000100077 | adrenergic, beta, receptor kinase 2 |
| **AFF4** | U-87 MG | ENSG00000072364 | AF4/FMR2 family, member 4 |
| **AGFG2** | HepG2 | ENSG00000106351 | ArfGAP with FG repeats 2 |
| **AGMAT** | HepG2 | ENSG00000116771 | agmatine ureohydrolase (agmatinase) |
| **AGRN** | ACHN | ENSG00000188157 | agrin |
| **AGT** | HepG2 | ENSG00000135744 | angiotensinogen (serpin peptidase inhibitor, clade A, member 8) |
| **AHR** | U-87 MG | ENSG00000106546 | aryl hydrocarbon receptor |
| **AHSG** | HepG2 | ENSG00000145192 | alpha-2-HS-glycoprotein |
| **AJUBA** | U-87 MG | ENSG00000129474 | ajuba LIM protein |
| **AK5** | SH-SY5Y | ENSG00000154027 | adenylate kinase 5 |
| **AKAP6** | SH-SY5Y | ENSG00000151320 | A kinase (PRKA) anchor protein 6 |
| **AKIRIN2** | SH-SY5Y | ENSG00000135334 | akirin 2 |
| **AKR1B10** | HepG2 | ENSG00000198074 | aldo-keto reductase family 1, member B10 (aldose reductase) |
| **AKR1C1** | HepG2 | ENSG00000187134 | aldo-keto reductase family 1, member C1 |
| **AKR1C2** | HepG2 | ENSG00000151632 | aldo-keto reductase family 1, member C2 |
| **AKR1C3** | HepG2 | ENSG00000196139 | aldo-keto reductase family 1, member C3 |
| **AKR1D1** | HepG2 | ENSG00000122787 | aldo-keto reductase family 1, member D1 |
| **AKT1S1** | ACHN | ENSG00000204673 | AKT1 substrate 1 (proline-rich) |
| **AL117190.1** | SH-SY5Y | ENSG00000221077 | NA |
| **ALCAM** | SH-SY5Y | ENSG00000170017 | activated leukocyte cell adhesion molecule |
| **ALK** | SH-SY5Y | ENSG00000171094 | anaplastic lymphoma receptor tyrosine kinase |
| **ALOXE3** | ACHN | ENSG00000179148 | arachidonate lipoxygenase 3 |
| **ALPL** | HepG2 | ENSG00000162551 | alkaline phosphatase, liver/bone/kidney |
| **AMBRA1** | ACHN | ENSG00000110497 | autophagy/beclin-1 regulator 1 |
| **AMOT** | hFOB1.19 | ENSG00000126016 | angiomotin |
| **AMOTL2** | HeLa-S3 | ENSG00000114019 | angiomotin like 2 |
| **AMZ2P1** | ACHN | ENSG00000214174 | archaelysin family metallopeptidase 2 pseudogene 1 |
| **ANAPC2** | IMR-32 | ENSG00000176248 | anaphase promoting complex subunit 2 |
| **ANK2** | SH-SY5Y | ENSG00000145362 | ankyrin 2, neuronal |
| **ANK3** | U-87 MG | ENSG00000151150 | ankyrin 3, node of Ranvier (ankyrin G) |
| **ANKLE2** | ACHN | ENSG00000176915 | ankyrin repeat and LEM domain containing 2 |
| **ANKRD1** | HepG2 | ENSG00000148677 | ankyrin repeat domain 1 (cardiac muscle) |
| **ANKRD11** | SK-N-SH | ENSG00000167522 | ankyrin repeat domain 11 |
| **ANKRD2** | ACHN | ENSG00000165887 | ankyrin repeat domain 2 (stretch responsive muscle) |
| **ANKRD54** | U-87 MG | ENSG00000100124 | ankyrin repeat domain 54 |
| **ANLN** | SH-SY5Y | ENSG00000011426 | anillin, actin binding protein |
| **ANP32E** | SH-SY5Y | ENSG00000143401 | acidic (leucine-rich) nuclear phosphoprotein 32 family, member E |
| **ANTXR2** | ACHN | ENSG00000163297 | anthrax toxin receptor 2 |
| **AP1B1** | ACHN | ENSG00000100280 | adaptor-related protein complex 1, beta 1 subunit |
| **AP1S2** | HepG2 | ENSG00000182287 | adaptor-related protein complex 1, sigma 2 subunit |
| **AP5Z1** | ACHN | ENSG00000242802 | adaptor-related protein complex 5, zeta 1 subunit |
| **APEX2** | HepG2 | ENSG00000169188 | APEX nuclease (apurinic/apyrimidinic endonuclease) 2 |
| **APLN** | U-87 MG | ENSG00000171388 | apelin |
| **APOE** | SH-SY5Y | ENSG00000130203 | apolipoprotein E |
| **APOL1** | HepG2 | ENSG00000100342 | apolipoprotein L, 1 |
| **APOL2** | HepG2 | ENSG00000128335 | apolipoprotein L, 2 |
| **AQP3** | SH-SY5Y | ENSG00000165272 | aquaporin 3 (Gill blood group) |
| **ARFIP2** | ACHN | ENSG00000132254 | ADP-ribosylation factor interacting protein 2 |
| **ARHGAP22** | SK-N-SH | ENSG00000128805 | Rho GTPase activating protein 22 |
| **ARHGAP31** | HepG2 | ENSG00000031081 | Rho GTPase activating protein 31 |
| **ARHGAP36** | SH-SY5Y | ENSG00000147256 | Rho GTPase activating protein 36 |
| **ARHGAP42** | HepG2 | ENSG00000165895 | Rho GTPase activating protein 42 |
| **ARHGEF39** | SH-SY5Y | ENSG00000137135 | Rho guanine nucleotide exchange factor (GEF) 39 |
| **ARHGEF40** | ACHN | ENSG00000165801 | Rho guanine nucleotide exchange factor (GEF) 40 |
| **ARID4A** | ACHN | ENSG00000032219 | AT rich interactive domain 4A (RBP1-like) |
| **ARID5A** | SH-SY5Y | ENSG00000196843 | AT rich interactive domain 5A (MRF1-like) |
| **ARL14EP** | ACHN | ENSG00000152219 | ADP-ribosylation factor-like 14 effector protein |
| **ARL5B** | U-87 MG | ENSG00000165997 | ADP-ribosylation factor-like 5B |
| **ARMC5** | SH-SY5Y | ENSG00000140691 | armadillo repeat containing 5 |
| **ARRDC3** | ACHN | ENSG00000113369 | arrestin domain containing 3 |
| **ARVCF** | ACHN | ENSG00000099889 | armadillo repeat gene deleted in velocardiofacial syndrome |
| **AS3MT** | SH-SY5Y | ENSG00000214435 | arsenic (+3 oxidation state) methyltransferase |
| **ASAP2** | HepG2 | ENSG00000151693 | ArfGAP with SH3 domain, ankyrin repeat and PH domain 2 |
| **ASB6** | SH-SY5Y | ENSG00000148331 | ankyrin repeat and SOCS box containing 6 |
| **ASCL1** | SH-SY5Y | ENSG00000139352 | achaete-scute complex homolog 1 (Drosophila) |
| **ASGR1** | HepG2 | ENSG00000141505 | asialoglycoprotein receptor 1 |
| **ASNS** | hFOB1.19 | ENSG00000070669 | asparagine synthetase (glutamine-hydrolyzing) |
| **ASPM** | SH-SY5Y | ENSG00000066279 | asp (abnormal spindle) homolog, microcephaly associated (Drosophila) |
| **ASS1P1** | IMR-32 | ENSG00000220517 | argininosuccinate synthetase 1 pseudogene 1 |
| **ATAD2** | SH-SY5Y | ENSG00000156802 | ATPase family, AAA domain containing 2 |
| **ATF4P4** | U-87 MG | ENSG00000256167 | activating transcription factor 4 pseudogene 4 |
| **ATF7IP** | ACHN | ENSG00000171681 | activating transcription factor 7 interacting protein |
| **ATG2A** | ACHN | ENSG00000110046 | autophagy related 2A |
| **ATP11A** | SH-SY5Y | ENSG00000068650 | ATPase, class VI, type 11A |
| **ATP2B2** | HepG2 | ENSG00000157087 | ATPase, Ca++ transporting, plasma membrane 2 |
| **ATP6AP1L** | HepG2 | ENSG00000205464 | ATPase, H+ transporting, lysosomal accessory protein 1-like |
| **ATP6V0A1** | ACHN | ENSG00000033627 | ATPase, H+ transporting, lysosomal V0 subunit a1 |
| **ATP6V0D1** | ACHN | ENSG00000159720 | ATPase, H+ transporting, lysosomal 38kDa, V0 subunit d1 |
| **ATP8B1** | ACHN | ENSG00000081923 | ATPase, aminophospholipid transporter, class I, type 8B, member 1 |
| **ATP8B3** | ACHN | ENSG00000130270 | ATPase, aminophospholipid transporter, class I, type 8B, member 3 |
| **ATRX** | SK-N-SH | ENSG00000085224 | alpha thalassemia/mental retardation syndrome X-linked |
| **AURKB** | SH-SY5Y | ENSG00000178999 | aurora kinase B |
| **AUTS2** | SH-SY5Y | ENSG00000158321 | autism susceptibility candidate 2 |
| **AVPI1** | SH-SY5Y | ENSG00000119986 | arginine vasopressin-induced 1 |
| **B3GNT4** | SH-SY5Y | ENSG00000176383 | UDP-GlcNAc:betaGal beta-1,3-N-acetylglucosaminyltransferase 4 |
| **B3GNT7** | HepG2 | ENSG00000156966 | UDP-GlcNAc:betaGal beta-1,3-N-acetylglucosaminyltransferase 7 |
| **B4GALT2** | HepG2 | ENSG00000117411 | UDP-Gal:betaGlcNAc beta 1,4- galactosyltransferase, polypeptide 2 |
| **BAG1** | SH-SY5Y | ENSG00000107262 | BCL2-associated athanogene |
| **BAHCC1** | ACHN | ENSG00000171282 | BAH and coiled-coil domain-containing protein 1 |
| **BBC3** | SH-SY5Y | ENSG00000105327 | BCL2 binding component 3 |
| **BCAM** | HepG2 | ENSG00000187244 | basal cell adhesion molecule (Lutheran blood group) |
| **BCAR1** | SH-SY5Y | ENSG00000050820 | breast cancer anti-estrogen resistance 1 |
| **BCAR3** | HepG2 | ENSG00000137936 | breast cancer anti-estrogen resistance 3 |
| **BCAT1** | ACHN | ENSG00000060982 | branched chain amino-acid transaminase 1, cytosolic |
| **BCL11B** | U-87 MG | ENSG00000127152 | B-cell CLL/lymphoma 11B (zinc finger protein) |
| **BCL2** | SH-SY5Y | ENSG00000171791 | B-cell CLL/lymphoma 2 |
| **BCL3** | ACHN | ENSG00000069399 | B-cell CLL/lymphoma 3 |
| **BCL6** | U-87 MG | ENSG00000113916 | B-cell CLL/lymphoma 6 |
| **BCRP3** | SH-SY5Y | ENSG00000215481 | breakpoint cluster region pseudogene 3 |
| **BDKRB1** | U-87 MG | ENSG00000100739 | bradykinin receptor B1 |
| **BEGAIN** | SH-SY5Y | ENSG00000183092 | brain-enriched guanylate kinase-associated |
| **BEST1** | HepG2 | ENSG00000167995 | bestrophin 1 |
| **BGN** | HeLa-S3 | ENSG00000182492 | biglycan |
| **BICC1** | ACHN | ENSG00000122870 | bicaudal C homolog 1 (Drosophila) |
| **BIK** | ACHN | ENSG00000100290 | BCL2-interacting killer (apoptosis-inducing) |
| **BIRC2** | SH-SY5Y | ENSG00000110330 | baculoviral IAP repeat containing 2 |
| **BIRC5** | SH-SY5Y | ENSG00000089685 | baculoviral IAP repeat containing 5 |
| **BLK** | SH-SY5Y | ENSG00000136573 | B lymphoid tyrosine kinase |
| **BLM** | SH-SY5Y | ENSG00000197299 | Bloom syndrome, RecQ helicase-like |
| **BMF** | HeLa-S3 | ENSG00000104081 | Bcl2 modifying factor |
| **BMPR2** | HepG2 | ENSG00000204217 | bone morphogenetic protein receptor, type II (serine/threonine kinase) |
| **BNIP3L** | HepG2 | ENSG00000104765 | BCL2/adenovirus E1B 19kDa interacting protein 3-like |
| **BRAF** | ACHN | ENSG00000157764 | v-raf murine sarcoma viral oncogene homolog B1 |
| **BRCA1** | SH-SY5Y | ENSG00000012048 | breast cancer 1, early onset |
| **BRCA2** | SH-SY5Y | ENSG00000139618 | breast cancer 2, early onset |
| **BRD2** | SH-SY5Y | ENSG00000204256 | bromodomain containing 2 |
| **BRI3BP** | SH-SY5Y | ENSG00000184992 | BRI3 binding protein |
| **BRIP1** | SH-SY5Y | ENSG00000136492 | BRCA1 interacting protein C-terminal helicase 1 |
| **BTG2** | ACHN | ENSG00000159388 | BTG family, member 2 |
| **BUB1** | SH-SY5Y | ENSG00000169679 | BUB1 mitotic checkpoint serine/threonine kinase |
| **BUB1B** | SH-SY5Y | ENSG00000156970 | BUB1 mitotic checkpoint serine/threonine kinase B |
| **BUD31** | SH-SY5Y | ENSG00000106245 | BUD31 homolog (*S. cerevisiae*) |
| **C11orf86** | HepG2 | ENSG00000173237 | chromosome 11 open reading frame 86 |
| **C12orf44** | SH-SY5Y | ENSG00000123395 | chromosome 12 open reading frame 44 |
| **C16orf72** | SH-SY5Y | ENSG00000182831 | chromosome 16 open reading frame 72 |
| **C17orf53** | ACHN | ENSG00000125319 | chromosome 17 open reading frame 53 |
| **C17orf96** | HepG2 | ENSG00000179294 | chromosome 17 open reading frame 96 |
| **C19orf21** | HepG2 | ENSG00000099812 | chromosome 19 open reading frame 21 |
| **C1orf106** | SH-SY5Y | ENSG00000163362 | chromosome 1 open reading frame 106 |
| **C1orf116** | ACHN | ENSG00000182795 | chromosome 1 open reading frame 116 |
| **C1orf186** | ACHN | ENSG00000196533 | chromosome 1 open reading frame 186 |
| **C1orf21** | SH-SY5Y | ENSG00000116667 | chromosome 1 open reading frame 21 |
| **C1orf51** | ACHN | ENSG00000159208 | chromosome 1 open reading frame 51 |
| **C20orf111** | SH-SY5Y | ENSG00000132823 | chromosome 20 open reading frame 111 |
| **C20orf166-AS1** | SH-SY5Y | ENSG00000174403 | C20orf166 antisense RNA 1 |
| **C21orf90** | SH-SY5Y | ENSG00000182912 | chromosome 21 open reading frame 90 |
| **C22orf29** | HepG2 | ENSG00000215012 | chromosome 22 open reading frame 29 |
| **C2CD2** | ACHN | ENSG00000157617 | C2 calcium-dependent domain containing 2 |
| **C2orf72** | HepG2 | ENSG00000204128 | chromosome 2 open reading frame 72 |
| **C3orf52** | SK-N-SH | ENSG00000114529 | chromosome 3 open reading frame 52 |
| **C4B** | HepG2 | ENSG00000224389 | complement component 4B (Chido blood group) |
| **C5** | HepG2 | ENSG00000106804 | complement component 5 |
| **C6orf15** | HepG2 | ENSG00000204542 | chromosome 6 open reading frame 15 |
| **C7orf63** | ACHN | ENSG00000105792 | chromosome 7 open reading frame 63 |
| **C8orf42** | SH-SY5Y | ENSG00000180190 | chromosome 8 open reading frame 42 |
| **C9orf89** | SH-SY5Y | ENSG00000165233 | chromosome 9 open reading frame 89 |
| **CABYR** | ACHN | ENSG00000154040 | calcium binding tyrosine-(Y)-phosphorylation regulated |
| **CACNA1G** | SH-SY5Y | ENSG00000006283 | calcium channel, voltage-dependent, T type, alpha 1G subunit |
| **CACNA2D2** | SH-SY5Y | ENSG00000007402 | calcium channel, voltage-dependent, alpha 2/delta subunit 2 |
| **CACNG4** | SH-SY5Y | ENSG00000075461 | calcium channel, voltage-dependent, gamma subunit 4 |
| **CALB2** | HepG2 | ENSG00000172137 | calbindin 2 |
| **CALM2** | ACHN | ENSG00000143933 | calmodulin 2 (phosphorylase kinase, delta) |
| **CALM3** | U-87 MG | ENSG00000160014 | calmodulin 3 (phosphorylase kinase, delta) |
| **CAPG** | HepG2 | ENSG00000042493 | capping protein (actin filament), gelsolin-like |
| **CAPN2** | HepG2 | ENSG00000162909 | calpain 2, (m/II) large subunit |
| **CAPN3** | HepG2 | ENSG00000092529 | calpain 3, (p94) |
| **CASC5** | SH-SY5Y | ENSG00000137812 | cancer susceptibility candidate 5 |
| **CAST** | HepG2 | ENSG00000153113 | calpastatin |
| **CBX5** | SH-SY5Y | ENSG00000094916 | chromobox homolog 5 |
| **CCDC108** | ACHN | ENSG00000181378 | coiled-coil domain containing 108 |
| **CCDC130** | ACHN | ENSG00000104957 | coiled-coil domain containing 130 |
| **CCDC157** | ACHN | ENSG00000187860 | coiled-coil domain containing 157 |
| **CCDC40** | ACHN | ENSG00000141519 | coiled-coil domain containing 40 |
| **CCDC84** | ACHN | ENSG00000186166 | coiled-coil domain containing 84 |
| **CCL2** | ACHN | ENSG00000108691 | chemokine (C-C motif) ligand 2 |
| **CCL5** | ACHN | ENSG00000161570 | chemokine (C-C motif) ligand 5 |
| **CCND1** | SH-SY5Y | ENSG00000110092 | cyclin D1 |
| **CCND2** | HepG2 | ENSG00000118971 | cyclin D2 |
| **CCR7** | HepG2 | ENSG00000126353 | chemokine (C-C motif) receptor 7 |
| **CD109** | HepG2 | ENSG00000156535 | CD109 molecule |
| **CD300C** | HepG2 | ENSG00000167850 | CD300c molecule |
| **CD300LB** | ACHN | ENSG00000178789 | CD300 molecule-like family member b |
| **CD55** | HepG2 | ENSG00000196352 | CD55 molecule, decay accelerating factor for complement (Cromer blood group) |
| **CD9** | HepG2 | ENSG00000010278 | CD9 molecule |
| **CD99P1** | ACHN | ENSG00000223773 | CD99 molecule pseudogene 1 |
| **CDA** | HepG2 | ENSG00000158825 | cytidine deaminase |
| **CDC20P1** | HepG2 | ENSG00000231007 | cell division cycle 20 pseudogene 1 |
| **CDC25A** | HepG2 | ENSG00000164045 | cell division cycle 25A |
| **CDC45** | SH-SY5Y | ENSG00000093009 | cell division cycle 45 |
| **CDC7** | SH-SY5Y | ENSG00000097046 | cell division cycle 7 |
| **CDCA7L** | SH-SY5Y | ENSG00000164649 | cell division cycle associated 7-like |
| **CDH15** | HepG2 | ENSG00000129910 | cadherin 15, type 1, M-cadherin (myotubule) |
| **CDH24** | SH-SY5Y | ENSG00000139880 | cadherin 24, type 2 |
| **CDH4** | SH-SY5Y | ENSG00000179242 | cadherin 4, type 1, R-cadherin (retinal) |
| **CDHR2** | SH-SY5Y | ENSG00000074276 | cadherin-related family member 2 |
| **CDIP1** | ACHN | ENSG00000089486 | cell death-inducing p53 target 1 |
| **CDK1** | SH-SY5Y | ENSG00000170312 | cyclin-dependent kinase 1 |
| **CDK11A** | ACHN | ENSG00000008128 | cyclin-dependent kinase 11A |
| **CDK2** | SH-SY5Y | ENSG00000123374 | cyclin-dependent kinase 2 |
| **CDK6** | U-87 MG | ENSG00000105810 | cyclin-dependent kinase 6 |
| **CEBPG** | U-87 MG | ENSG00000153879 | CCAAT/enhancer binding protein (C/EBP), gamma |
| **CELF2** | SH-SY5Y | ENSG00000048740 | CUGBP, Elav-like family member 2 |
| **CENPE** | SH-SY5Y | ENSG00000138778 | centromere protein E, 312kDa |
| **CENPF** | SH-SY5Y | ENSG00000117724 | centromere protein F, 350/400kDa |
| **CENPI** | SH-SY5Y | ENSG00000102384 | centromere protein I |
| **CENPK** | SH-SY5Y | ENSG00000123219 | centromere protein K |
| **CENPO** | SH-SY5Y | ENSG00000138092 | centromere protein O |
| **CEP192** | SH-SY5Y | ENSG00000101639 | centrosomal protein 192kDa |
| **CEP55** | SH-SY5Y | ENSG00000138180 | centrosomal protein 55kDa |
| **CEP78** | SH-SY5Y | ENSG00000148019 | centrosomal protein 78kDa |
| **CEP85L** | ACHN | ENSG00000111860 | centrosomal protein 85kDa-like |
| **CFLAR** | ACHN | ENSG00000003402 | CASP8 and FADD-like apoptosis regulator |
| **CHAC1** | SH-SY5Y | ENSG00000128965 | ChaC, cation transport regulator homolog 1 (*E. coli*) |
| **CHADL** | HepG2 | ENSG00000100399 | chondroadherin-like |
| **CHAF1A** | SH-SY5Y | ENSG00000167670 | chromatin assembly factor 1, subunit A (p150) |
| **CHAF1B** | SH-SY5Y | ENSG00000159259 | chromatin assembly factor 1, subunit B (p60) |
| **CHD2** | ACHN | ENSG00000173575 | chromodomain helicase DNA binding protein 2 |
| **CHGB** | SH-SY5Y | ENSG00000089199 | chromogranin B (secretogranin 1) |
| **CHI3L1** | HepG2 | ENSG00000133048 | chitinase 3-like 1 (cartilage glycoprotein-39) |
| **CHKA** | ACHN | ENSG00000110721 | choline kinase alpha |
| **CHMP1B** | U-87 MG | ENSG00000255112 | charged multivesicular body protein 1B |
| **CHN1** | hFOB1.19 | ENSG00000128656 | chimerin 1 |
| **CHRM2** | SH-SY5Y | ENSG00000181072 | cholinergic receptor, muscarinic 2 |
| **CHRM3** | SH-SY5Y | ENSG00000133019 | cholinergic receptor, muscarinic 3 |
| **CHST11** | HepG2 | ENSG00000171310 | carbohydrate (chondroitin 4) sulfotransferase 11 |
| **CHST2** | hFOB1.19 | ENSG00000175040 | carbohydrate (N-acetylglucosamine-6-O) sulfotransferase 2 |
| **CHST7** | SH-SY5Y | ENSG00000147119 | carbohydrate (N-acetylglucosamine 6-O) sulfotransferase 7 |
| **CHTF18** | SH-SY5Y | ENSG00000127586 | CTF18, chromosome transmission fidelity factor 18 homolog (*S. cerevisiae*) |
| **CIC** | ACHN | ENSG00000079432 | capicua transcriptional repressor |
| **CIT** | SH-SY5Y | ENSG00000122966 | citron (rho-interacting, serine/threonine kinase 21) |
| **CKAP2L** | SH-SY5Y | ENSG00000169607 | cytoskeleton associated protein 2-like |
| **CLCN3** | SH-SY5Y | ENSG00000109572 | chloride channel, voltage-sensitive 3 |
| **CLCN4** | ACHN | ENSG00000073464 | chloride channel, voltage-sensitive 4 |
| **CLCN6** | ACHN | ENSG00000011021 | chloride channel, voltage-sensitive 6 |
| **CLDN11** | HepG2 | ENSG00000013297 | claudin 11 |
| **CLK1** | SH-SY5Y | ENSG00000013441 | CDC-like kinase 1 |
| **CLMP** | ACHN | ENSG00000166250 | CXADR-like membrane protein |
| **CLSPN** | SH-SY5Y | ENSG00000092853 | claspin |
| **CLTB** | HepG2 | ENSG00000175416 | clathrin, light chain B |
| **CLUHP3** | ACHN | ENSG00000131797 | clustered mitochondria (cluA/CLU1) homolog pseudogene 3 |
| **CNIH** | ACHN | ENSG00000100528 | cornichon homolog (Drosophila) |
| **CNIH3** | SH-SY5Y | ENSG00000143786 | cornichon homolog 3 (Drosophila) |
| **CNOT4** | ACHN | ENSG00000080802 | CCR4-NOT transcription complex, subunit 4 |
| **CNTN1** | SH-SY5Y | ENSG00000018236 | contactin 1 |
| **CNTN6** | ACHN | ENSG00000134115 | contactin 6 |
| **CNTNAP1** | SH-SY5Y | ENSG00000108797 | contactin associated protein 1 |
| **CNTNAP2** | SH-SY5Y | ENSG00000174469 | contactin associated protein-like 2 |
| **CNTROB** | ACHN | ENSG00000170037 | centrobin, centrosomal BRCA2 interacting protein |
| **COL10A1** | hFOB1.19 | ENSG00000123500 | collagen, type X, alpha 1 |
| **COL2A1** | HepG2 | ENSG00000139219 | collagen, type II, alpha 1 |
| **COL5A1** | SH-SY5Y | ENSG00000130635 | collagen, type V, alpha 1 |
| **COLEC10** | ACHN | ENSG00000184374 | collectin sub-family member 10 (C-type lectin) |
| **COQ9** | ACHN | ENSG00000088682 | coenzyme Q9 homolog (*S. cerevisiae*) |
| **CORIN** | SH-SY5Y | ENSG00000145244 | corin, serine peptidase |
| **CPLX2** | HepG2 | ENSG00000145920 | complexin 2 |
| **CPN2** | HepG2 | ENSG00000178772 | carboxypeptidase N, polypeptide 2 |
| **CPXM1** | HepG2 | ENSG00000088882 | carboxypeptidase X (M14 family), member 1 |
| **CREB3L2** | SH-SY5Y | ENSG00000182158 | cAMP responsive element binding protein 3-like 2 |
| **CREB5** | ACHN | ENSG00000146592 | cAMP responsive element binding protein 5 |
| **CREBRF** | U-87 MG | ENSG00000164463 | CREB3 regulatory factor |
| **CRY2** | ACHN | ENSG00000121671 | cryptochrome 2 (photolyase-like) |
| **CSDC2** | ACHN | ENSG00000172346 | cold shock domain containing C2, RNA binding |
| **CSF1** | ACHN | ENSG00000184371 | colony stimulating factor 1 (macrophage) |
| **CSMD2** | SH-SY5Y | ENSG00000121904 | CUB and Sushi multiple domains 2 |
| **CST5** | HepG2 | ENSG00000170367 | cystatin D |
| **CST7** | U-2 Os | ENSG00000077984 | cystatin F (leukocystatin) |
| **CTD-2547E10.2** | ACHN | ENSG00000180747 | NA |
| **CTHRC1** | HepG2 | ENSG00000164932 | collagen triple helix repeat containing 1 |
| **CTNNA1** | HepG2 | ENSG00000044115 | catenin (cadherin-associated protein), alpha 1, 102kDa |
| **CTNNAL1** | SH-SY5Y | ENSG00000119326 | catenin (cadherin-associated protein), alpha-like 1 |
| **CTNNAP1** | HepG2 | ENSG00000249026 | NA |
| **CTSE** | HepG2 | ENSG00000196188 | cathepsin E |
| **CUBN** | SH-SY5Y | ENSG00000107611 | cubilin (intrinsic factor-cobalamin receptor) |
| **CUX2** | SH-SY5Y | ENSG00000111249 | cut-like homeobox 2 |
| **CXCL1** | ACHN | ENSG00000163739 | chemokine (C-X-C motif) ligand 1 (melanoma growth stimulating activity, alpha) |
| **CXCL2** | ACHN | ENSG00000081041 | chemokine (C-X-C motif) ligand 2 |
| **CXCL3** | ACHN | ENSG00000163734 | chemokine (C-X-C motif) ligand 3 |
| **CXCR4** | SH-SY5Y | ENSG00000121966 | chemokine (C-X-C motif) receptor 4 |
| **CYP1A1** | ACHN | ENSG00000140465 | cytochrome P450, family 1, subfamily A, polypeptide 1 |
| **CYP1B1** | hFOB1.19 | ENSG00000138061 | cytochrome P450, family 1, subfamily B, polypeptide 1 |
| **CYP26B1** | U-87 MG | ENSG00000003137 | cytochrome P450, family 26, subfamily B, polypeptide 1 |
| **CYP27A1** | HepG2 | ENSG00000135929 | cytochrome P450, family 27, subfamily A, polypeptide 1 |
| **CYP2W1** | HepG2 | ENSG00000073067 | cytochrome P450, family 2, subfamily W, polypeptide 1 |
| **CYP3A5** | ACHN | ENSG00000106258 | cytochrome P450, family 3, subfamily A, polypeptide 5 |
| **CYR61** | HepG2 | ENSG00000142871 | cysteine-rich, angiogenic inducer, 61 |
| **CYTH4** | ACHN | ENSG00000100055 | cytohesin 4 |
| **DAGLB** | ACHN | ENSG00000164535 | diacylglycerol lipase, beta |
| **DAK** | HepG2 | ENSG00000149476 | dihydroxyacetone kinase 2 homolog (*S. cerevisiae*) |
| **DAPK3** | ACHN | ENSG00000167657 | death-associated protein kinase 3 |
| **DCDC2** | HepG2 | ENSG00000146038 | doublecortin domain containing 2 |
| **DCLK1** | SH-SY5Y | ENSG00000133083 | doublecortin-like kinase 1 |
| **DCP1B** | ACHN | ENSG00000151065 | DCP1 decapping enzyme homolog B (*S. cerevisiae*) |
| **DCX** | SH-SY5Y | ENSG00000077279 | doublecortin |
| **DDB2** | ACHN | ENSG00000134574 | damage-specific DNA binding protein 2, 48kDa |
| **DDI2** | HepG2 | ENSG00000197312 | DNA-damage inducible 1 homolog 2 (*S. cerevisiae*) |
| **DDX11** | ACHN | ENSG00000013573 | DEAD/H (Asp-Glu-Ala-Asp/His) box helicase 11 |
| **DDX12P** | SH-SY5Y | ENSG00000214826 | DEAD/H (Asp-Glu-Ala-Asp/His) box polypeptide 12, pseudogene |
| **DDX39A** | ACHN | ENSG00000123136 | DEAD (Asp-Glu-Ala-Asp) box polypeptide 39A |
| **DGKH** | U-87 MG | ENSG00000102780 | diacylglycerol kinase, eta |
| **DHCR24** | SH-SY5Y | ENSG00000116133 | 24-dehydrocholesterol reductase |
| **DHFR** | SH-SY5Y | ENSG00000228716 | dihydrofolate reductase |
| **DHRS2** | ACHN | ENSG00000100867 | dehydrogenase/reductase (SDR family) member 2 |
| **DHRS3** | SH-SY5Y | ENSG00000162496 | dehydrogenase/reductase (SDR family) member 3 |
| **DHX9** | ACHN | ENSG00000135829 | DEAH (Asp-Glu-Ala-His) box polypeptide 9 |
| **DHX9P1** | ACHN | ENSG00000228002 | DEAH (Asp-Glu-Ala-His) box polypeptide 9 pseudogene 1 |
| **DIAPH3** | SH-SY5Y | ENSG00000139734 | diaphanous homolog 3 (Drosophila) |
| **DIRAS3** | hFOB1.19 | ENSG00000162595 | DIRAS family, GTP-binding RAS-like 3 |
| **DIS3L** | ACHN | ENSG00000166938 | DIS3 mitotic control homolog (*S. cerevisiae*)-like |
| **DIS3L2** | ACHN | ENSG00000144535 | DIS3 mitotic control homolog (*S. cerevisiae*)-like 2 |
| **DLC1** | U-87 MG | ENSG00000164741 | deleted in liver cancer 1 |
| **DLGAP5** | SH-SY5Y | ENSG00000126787 | discs, large (Drosophila) homolog-associated protein 5 |
| **DLX1** | HepG2 | ENSG00000144355 | distal-less homeobox 1 |
| **DLX2** | HepG2 | ENSG00000115844 | distal-less homeobox 2 |
| **DLX3** | HepG2 | ENSG00000064195 | distal-less homeobox 3 |
| **DNA2** | SH-SY5Y | ENSG00000138346 | DNA replication helicase 2 homolog (yeast) |
| **DNAJB1** | U-87 MG | ENSG00000132002 | DnaJ (Hsp40) homolog, subfamily B, member 1 |
| **DNAJB4** | ACHN | ENSG00000162616 | DnaJ (Hsp40) homolog, subfamily B, member 4 |
| **DNAJB5** | ACHN | ENSG00000137094 | DnaJ (Hsp40) homolog, subfamily B, member 5 |
| **DNER** | SH-SY5Y | ENSG00000187957 | delta/notch-like EGF repeat containing |
| **DNMBP** | SH-SY5Y | ENSG00000107554 | dynamin binding protein |
| **DOCK4** | HepG2 | ENSG00000128512 | dedicator of cytokinesis 4 |
| **DOK4** | SH-SY5Y | ENSG00000125170 | docking protein 4 |
| **DPEP1** | HepG2 | ENSG00000015413 | dipeptidase 1 (renal) |
| **DPYSL2** | ACHN | ENSG00000092964 | dihydropyrimidinase-like 2 |
| **DSN1** | SH-SY5Y | ENSG00000149636 | DSN1, MIND kinetochore complex component, homolog (*S. cerevisiae*) |
| **DTL** | SH-SY5Y | ENSG00000143476 | denticleless E3 ubiquitin protein ligase homolog (Drosophila) |
| **DTX3L** | ACHN | ENSG00000163840 | deltex 3-like (Drosophila) |
| **DUS3L** | ACHN | ENSG00000141994 | dihydrouridine synthase 3-like (*S. cerevisiae*) |
| **DUSP1** | SK-N-SH | ENSG00000120129 | dual specificity phosphatase 1 |
| **DUSP14** | ACHN | ENSG00000161326 | dual specificity phosphatase 14 |
| **DUSP16** | ACHN | ENSG00000111266 | dual specificity phosphatase 16 |
| **DUSP8** | ACHN | ENSG00000184545 | dual specificity phosphatase 8 |
| **DUSP9** | HepG2 | ENSG00000130829 | dual specificity phosphatase 9 |
| **DYNLL1** | ACHN | ENSG00000088986 | dynein, light chain, LC8-type 1 |
| **DYRK1B** | ACHN | ENSG00000105204 | dual-specificity tyrosine-(Y)-phosphorylation regulated kinase 1B |
| **DYSF** | U-2 Os | ENSG00000135636 | dysferlin, limb girdle muscular dystrophy 2B (autosomal recessive) |
| **DYX1C1** | SH-SY5Y | ENSG00000256061 | dyslexia susceptibility 1 candidate 1 |
| **E2F2** | SH-SY5Y | ENSG00000007968 | E2F transcription factor 2 |
| **E2F8** | SH-SY5Y | ENSG00000129173 | E2F transcription factor 8 |
| **ECEL1** | SH-SY5Y | ENSG00000171551 | endothelin converting enzyme-like 1 |
| **ECM1** | SH-SY5Y | ENSG00000143369 | extracellular matrix protein 1 |
| **ECT2** | SH-SY5Y | ENSG00000114346 | epithelial cell transforming sequence 2 oncogene |
| **EDA2R** | U-87 MG | ENSG00000131080 | ectodysplasin A2 receptor |
| **EDIL3** | SH-SY5Y | ENSG00000164176 | EGF-like repeats and discoidin I-like domains 3 |
| **EDN1** | U-2 Os | ENSG00000078401 | endothelin 1 |
| **EDN2** | ACHN | ENSG00000127129 | endothelin 2 |
| **EEF1A2** | HepG2 | ENSG00000101210 | eukaryotic translation elongation factor 1 alpha 2 |
| **EFCAB7** | ACHN | ENSG00000203965 | EF-hand calcium binding domain 7 |
| **EFHD2** | SH-SY5Y | ENSG00000142634 | EF-hand domain family, member D2 |
| **EFR3B** | SH-SY5Y | ENSG00000084710 | EFR3 homolog B (*S. cerevisiae*) |
| **EFS** | SH-SY5Y | ENSG00000100842 | embryonal Fyn-associated substrate |
| **EGFR** | HeLa-S3 | ENSG00000146648 | epidermal growth factor receptor |
| **EGR3** | SH-SY5Y | ENSG00000179388 | early growth response 3 |
| **EIF1** | SH-SY5Y | ENSG00000173812 | eukaryotic translation initiation factor 1 |
| **EIF4EBP1** | HepG2 | ENSG00000187840 | eukaryotic translation initiation factor 4E binding protein 1 |
| **ELAVL4** | SH-SY5Y | ENSG00000162374 | ELAV (embryonic lethal, abnormal vision, Drosophila)-like 4 |
| **ELF3** | ACHN | ENSG00000163435 | E74-like factor 3 (ets domain transcription factor, epithelial-specific ) |
| **ELF4** | HepG2 | ENSG00000102034 | E74-like factor 4 (ets domain transcription factor) |
| **ELOVL4** | SH-SY5Y | ENSG00000118402 | ELOVL fatty acid elongase 4 |
| **ELOVL6** | ACHN | ENSG00000170522 | ELOVL fatty acid elongase 6 |
| **EML4** | SH-SY5Y | ENSG00000143924 | echinoderm microtubule associated protein like 4 |
| **EML5** | SH-SY5Y | ENSG00000165521 | echinoderm microtubule associated protein like 5 |
| **EMP2** | SH-SY5Y | ENSG00000213853 | epithelial membrane protein 2 |
| **EMP3** | HepG2 | ENSG00000142227 | epithelial membrane protein 3 |
| **ENAH** | HepG2 | ENSG00000154380 | enabled homolog (Drosophila) |
| **ENTPD2** | HepG2 | ENSG00000054179 | ectonucleoside triphosphate diphosphohydrolase 2 |
| **EP400** | SH-SY5Y | ENSG00000183495 | E1A binding protein p400 |
| **EPC1** | ACHN | ENSG00000120616 | enhancer of polycomb homolog 1 (Drosophila) |
| **EPC2** | SH-SY5Y | ENSG00000135999 | enhancer of polycomb homolog 2 (Drosophila) |
| **EPG5** | HepG2 | ENSG00000152223 | ectopic P-granules autophagy protein 5 homolog (*C. elegans*) |
| **EPHB1** | SH-SY5Y | ENSG00000154928 | EPH receptor B1 |
| **EPHB4** | HepG2 | ENSG00000196411 | EPH receptor B4 |
| **EPS8L2** | ACHN | ENSG00000177106 | EPS8-like 2 |
| **EPS8L3** | HepG2 | ENSG00000198758 | EPS8-like 3 |
| **ERBB2IP** | U-87 MG | ENSG00000112851 | erbb2 interacting protein |
| **ERCC6L** | SH-SY5Y | ENSG00000186871 | excision repair cross-complementing rodent repair deficiency, complementation group 6-like |
| **ERF** | SH-SY5Y | ENSG00000105722 | Ets2 repressor factor |
| **ERN1** | U-87 MG | ENSG00000178607 | endoplasmic reticulum to nucleus signaling 1 |
| **ERRFI1** | HepG2 | ENSG00000116285 | ERBB receptor feedback inhibitor 1 |
| **ESCO2** | SH-SY5Y | ENSG00000171320 | establishment of cohesion 1 homolog 2 (*S. cerevisiae*) |
| **ESM1** | U-2 Os | ENSG00000164283 | endothelial cell-specific molecule 1 |
| **ESRRG** | IMR-32 | ENSG00000196482 | estrogen-related receptor gamma |
| **ETS2** | U-87 MG | ENSG00000157557 | v-ets erythroblastosis virus E26 oncogene homolog 2 (avian) |
| **ETV4** | IMR-32 | ENSG00000175832 | ets variant 4 |
| **ETV5** | IMR-32 | ENSG00000244405 | ets variant 5 |
| **ETV7** | ACHN | ENSG00000010030 | ets variant 7 |
| **EVC** | ACHN | ENSG00000072840 | Ellis van Creveld syndrome |
| **EVI2A** | U-87 MG | ENSG00000126860 | ecotropic viral integration site 2A |
| **EXO1** | SH-SY5Y | ENSG00000174371 | exonuclease 1 |
| **EZH2** | ACHN | ENSG00000106462 | enhancer of zeste homolog 2 (Drosophila) |
| **F2RL1** | ACHN | ENSG00000164251 | coagulation factor II (thrombin) receptor-like 1 |
| **FABP1** | HepG2 | ENSG00000163586 | fatty acid binding protein 1, liver |
| **FADD** | SH-SY5Y | ENSG00000168040 | Fas (TNFRSF6)-associated via death domain |
| **FADS1** | HepG2 | ENSG00000149485 | fatty acid desaturase 1 |
| **FADS3** | SH-SY5Y | ENSG00000221968 | fatty acid desaturase 3 |
| **FAM101B** | hFOB1.19 | ENSG00000183688 | family with sequence similarity 101, member B |
| **FAM111B** | SH-SY5Y | ENSG00000189057 | family with sequence similarity 111, member B |
| **FAM129A** | SH-SY5Y | ENSG00000135842 | family with sequence similarity 129, member A |
| **FAM163A** | SH-SY5Y | ENSG00000143340 | family with sequence similarity 163, member A |
| **FAM167A** | SH-SY5Y | ENSG00000154319 | family with sequence similarity 167, member A |
| **FAM179B** | ACHN | ENSG00000198718 | family with sequence similarity 179, member B |
| **FAM219A** | ACHN | ENSG00000164970 | family with sequence similarity 219, member A |
| **FAM222A** | SH-SY5Y | ENSG00000139438 | family with sequence similarity 222, member A |
| **FAM222A-AS1** | SH-SY5Y | ENSG00000255650 | FAM222A antisense RNA 1 |
| **FAM46A** | SH-SY5Y | ENSG00000112773 | family with sequence similarity 46, member A |
| **FAM46B** | ACHN | ENSG00000158246 | family with sequence similarity 46, member B |
| **FAM65C** | SH-SY5Y | ENSG00000042062 | family with sequence similarity 65, member C |
| **FAM83G** | ACHN | ENSG00000188522 | family with sequence similarity 83, member G |
| **FAM87B** | U-87 MG | ENSG00000177757 | family with sequence similarity 87, member B |
| **FANCA** | SH-SY5Y | ENSG00000187741 | Fanconi anemia, complementation group A |
| **FANCD2** | SH-SY5Y | ENSG00000144554 | Fanconi anemia, complementation group D2 |
| **FANCG** | SH-SY5Y | ENSG00000221829 | Fanconi anemia, complementation group G |
| **FASN** | HepG2 | ENSG00000169710 | fatty acid synthase |
| **FAT4** | U-87 MG | ENSG00000196159 | FAT tumor suppressor homolog 4 (Drosophila) |
| **FBRS** | SH-SY5Y | ENSG00000156860 | fibrosin |
| **FBXO31** | HepG2 | ENSG00000103264 | F-box protein 31 |
| **FBXO32** | U-87 MG | ENSG00000156804 | F-box protein 32 |
| **FCER1G** | HepG2 | ENSG00000158869 | Fc fragment of IgE, high affinity I, receptor for; gamma polypeptide |
| **FDXR** | ACHN | ENSG00000161513 | ferredoxin reductase |
| **FEN1** | SH-SY5Y | ENSG00000168496 | flap structure-specific endonuclease 1 |
| **FEZ1** | SH-SY5Y | ENSG00000149557 | fasciculation and elongation protein zeta 1 (zygin I) |
| **FGA** | HepG2 | ENSG00000171560 | fibrinogen alpha chain |
| **FGF10** | IMR-32 | ENSG00000070193 | fibroblast growth factor 10 |
| **FGF2** | U-87 MG | ENSG00000138685 | fibroblast growth factor 2 (basic) |
| **FGFR4** | HepG2 | ENSG00000160867 | fibroblast growth factor receptor 4 |
| **FGFRL1** | hFOB1.19 | ENSG00000127418 | fibroblast growth factor receptor-like 1 |
| **FHDC1** | HepG2 | ENSG00000137460 | FH2 domain containing 1 |
| **FHL2** | HepG2 | ENSG00000115641 | four and a half LIM domains 2 |
| **FHOD3** | HepG2 | ENSG00000134775 | formin homology 2 domain containing 3 |
| **FIGNL1** | SH-SY5Y | ENSG00000132436 | fidgetin-like 1 |
| **FLG** | HepG2 | ENSG00000143631 | filaggrin |
| **FLNB** | SH-SY5Y | ENSG00000136068 | filamin B, beta |
| **FLRT2** | U-87 MG | ENSG00000185070 | fibronectin leucine rich transmembrane protein 2 |
| **FMN1** | U-2 Os | ENSG00000248905 | formin 1 |
| **FNDC5** | IMR-32 | ENSG00000160097 | fibronectin type III domain containing 5 |
| **FOLR1** | HepG2 | ENSG00000110195 | folate receptor 1 (adult) |
| **FOS** | SH-SY5Y | ENSG00000170345 | FBJ murine osteosarcoma viral oncogene homolog |
| **FOSB** | SH-SY5Y | ENSG00000125740 | FBJ murine osteosarcoma viral oncogene homolog B |
| **FOXJ1** | ACHN | ENSG00000129654 | forkhead box J1 |
| **FOXK1** | SK-N-SH | ENSG00000164916 | forkhead box K1 |
| **FOXO1** | SH-SY5Y | ENSG00000150907 | forkhead box O1 |
| **FRAS1** | SH-SY5Y | ENSG00000138759 | Fraser syndrome 1 |
| **FRG1** | SH-SY5Y | ENSG00000109536 | FSHD region gene 1 |
| **FRMD3** | SH-SY5Y | ENSG00000172159 | FERM domain containing 3 |
| **FRMD6** | U-87 MG | ENSG00000139926 | FERM domain containing 6 |
| **FRS2** | U-87 MG | ENSG00000166225 | fibroblast growth factor receptor substrate 2 |
| **FSTL4** | ACHN | ENSG00000053108 | follistatin-like 4 |
| **FSTL5** | SH-SY5Y | ENSG00000168843 | follistatin-like 5 |
| **FTH1** | SH-SY5Y | ENSG00000167996 | ferritin, heavy polypeptide 1 |
| **FTH1P7** | SH-SY5Y | ENSG00000232187 | ferritin, heavy polypeptide 1 pseudogene 7 |
| **FUCA1** | ACHN | ENSG00000179163 | fucosidase, alpha-L- 1, tissue |
| **FZD1** | hFOB1.19 | ENSG00000157240 | frizzled family receptor 1 |
| **FZD4** | HepG2 | ENSG00000174804 | frizzled family receptor 4 |
| **GABRQ** | SH-SY5Y | ENSG00000147402 | gamma-aminobutyric acid (GABA) A receptor, theta |
| **GADD45G** | ACHN | ENSG00000130222 | growth arrest and DNA-damage-inducible, gamma |
| **GALNT10** | HepG2 | ENSG00000164574 | UDP-N-acetyl-alpha-D-galactosamine:polypeptide N-acetylgalactosaminyltransferase 10 (GalNAc-T10) |
| **GALNT13** | SH-SY5Y | ENSG00000144278 | UDP-N-acetyl-alpha-D-galactosamine:polypeptide N-acetylgalactosaminyltransferase 13 (GalNAc-T13) |
| **GALNT18** | ACHN | ENSG00000110328 | UDP-N-acetyl-alpha-D-galactosamine:polypeptide N-acetylgalactosaminyltransferase 18 |
| **GALNT2** | SH-SY5Y | ENSG00000143641 | UDP-N-acetyl-alpha-D-galactosamine:polypeptide N-acetylgalactosaminyltransferase 2 (GalNAc-T2) |
| **GALNT6** | SH-SY5Y | ENSG00000139629 | UDP-N-acetyl-alpha-D-galactosamine:polypeptide N-acetylgalactosaminyltransferase 6 (GalNAc-T6) |
| **GALNT9** | HepG2 | ENSG00000182870 | UDP-N-acetyl-alpha-D-galactosamine:polypeptide N-acetylgalactosaminyltransferase 9 (GalNAc-T9) |
| **GAP43** | SH-SY5Y | ENSG00000172020 | growth associated protein 43 |
| **GARS** | SH-SY5Y | ENSG00000106105 | glycyl-tRNA synthetase |
| **GAS2L1** | U-87 MG | ENSG00000185340 | growth arrest-specific 2 like 1 |
| **GAS2L3** | ACHN | ENSG00000139354 | growth arrest-specific 2 like 3 |
| **GAS6** | SH-SY5Y | ENSG00000183087 | growth arrest-specific 6 |
| **GAS8** | ACHN | ENSG00000141013 | growth arrest-specific 8 |
| **GBF1** | ACHN | ENSG00000107862 | golgi brefeldin A resistant guanine nucleotide exchange factor 1 |
| **GBP2** | ACHN | ENSG00000162645 | guanylate binding protein 2, interferon-inducible |
| **GBP5** | ACHN | ENSG00000154451 | guanylate binding protein 5 |
| **GCC2** | HepG2 | ENSG00000135968 | GRIP and coiled-coil domain containing 2 |
| **GDNF** | U-87 MG | ENSG00000168621 | glial cell derived neurotrophic factor |
| **GDPD5** | ACHN | ENSG00000158555 | glycerophosphodiester phosphodiesterase domain containing 5 |
| **GEM** | ACHN | ENSG00000164949 | GTP binding protein overexpressed in skeletal muscle |
| **GFRA1** | IMR-32 | ENSG00000151892 | GDNF family receptor alpha 1 |
| **GGT1** | ACHN | ENSG00000100031 | gamma-glutamyltransferase 1 |
| **GGT5** | HepG2 | ENSG00000099998 | gamma-glutamyltransferase 5 |
| **GGT7** | ACHN | ENSG00000131067 | gamma-glutamyltransferase 7 |
| **GINS1** | HepG2 | ENSG00000101003 | GINS complex subunit 1 (Psf1 homolog) |
| **GINS2** | SH-SY5Y | ENSG00000131153 | GINS complex subunit 2 (Psf2 homolog) |
| **GINS4** | SH-SY5Y | ENSG00000147536 | GINS complex subunit 4 (Sld5 homolog) |
| **GJB1** | HepG2 | ENSG00000169562 | gap junction protein, beta 1, 32kDa |
| **GK** | HepG2 | ENSG00000198814 | glycerol kinase |
| **GLIPR1** | HepG2 | ENSG00000139278 | GLI pathogenesis-related 1 |
| **GLIS3** | U-87 MG | ENSG00000107249 | GLIS family zinc finger 3 |
| **GLP2R** | ACHN | ENSG00000065325 | glucagon-like peptide 2 receptor |
| **GLUD1** | HepG2 | ENSG00000148672 | glutamate dehydrogenase 1 |
| **GLUD2** | HepG2 | ENSG00000182890 | glutamate dehydrogenase 2 |
| **GLYCTK** | HepG2 | ENSG00000168237 | glycerate kinase |
| **GMCL1** | ACHN | ENSG00000087338 | germ cell-less, spermatogenesis associated 1 |
| **GMFG** | HepG2 | ENSG00000130755 | glia maturation factor, gamma |
| **GMNN** | SH-SY5Y | ENSG00000112312 | geminin, DNA replication inhibitor |
| **GNG4** | HepG2 | ENSG00000168243 | guanine nucleotide binding protein (G protein), gamma 4 |
| **GNPTAB** | ACHN | ENSG00000111670 | N-acetylglucosamine-1-phosphate transferase, alpha and beta subunits |
| **GOLGB1** | U-87 MG | ENSG00000173230 | golgin B1 |
| **GOLM1** | HepG2 | ENSG00000135052 | golgi membrane protein 1 |
| **GORAB** | ACHN | ENSG00000120370 | golgin, RAB6-interacting |
| **GOT1** | SH-SY5Y | ENSG00000120053 | glutamic-oxaloacetic transaminase 1, soluble (aspartate aminotransferase 1) |
| **GPC6** | SH-SY5Y | ENSG00000183098 | glypican 6 |
| **GPD1** | HepG2 | ENSG00000167588 | glycerol-3-phosphate dehydrogenase 1 (soluble) |
| **GPR1** | U-87 MG | ENSG00000183671 | G protein-coupled receptor 1 |
| **GPR176** | ACHN | ENSG00000166073 | G protein-coupled receptor 176 |
| **GPR64** | SH-SY5Y | ENSG00000173698 | G protein-coupled receptor 64 |
| **GPR68** | hFOB1.19 | ENSG00000119714 | G protein-coupled receptor 68 |
| **GPR75** | HepG2 | ENSG00000119737 | G protein-coupled receptor 75 |
| **GPR98** | SH-SY5Y | ENSG00000164199 | G protein-coupled receptor 98 |
| **GPT2** | SH-SY5Y | ENSG00000166123 | glutamic pyruvate transaminase (alanine aminotransferase) 2 |
| **GPX2** | HepG2 | ENSG00000176153 | glutathione peroxidase 2 (gastrointestinal) |
| **GRB10** | HepG2 | ENSG00000106070 | growth factor receptor-bound protein 10 |
| **GRHL3** | ACHN | ENSG00000158055 | grainyhead-like 3 (Drosophila) |
| **GTPBP2** | U-87 MG | ENSG00000172432 | GTP binding protein 2 |
| **GTPBP3** | HepG2 | ENSG00000130299 | GTP binding protein 3 (mitochondrial) |
| **GZMB** | HepG2 | ENSG00000100453 | granzyme B (granzyme 2, cytotoxic T-lymphocyte-associated serine esterase 1) |
| **H2AFJ** | ACHN | ENSG00000246705 | H2A histone family, member J |
| **H2AFV** | SH-SY5Y | ENSG00000105968 | H2A histone family, member V |
| **H2AFX** | HepG2 | ENSG00000188486 | H2A histone family, member X |
| **HADH** | U-87 MG | ENSG00000138796 | hydroxyacyl-CoA dehydrogenase |
| **HAS2** | hFOB1.19 | ENSG00000170961 | hyaluronan synthase 2 |
| **HCFC1** | HepG2 | ENSG00000172534 | host cell factor C1 (VP16-accessory protein) |
| **HCFC2** | ACHN | ENSG00000111727 | host cell factor C2 |
| **HCP5** | ACHN | ENSG00000206337 | HLA complex P5 (non-protein coding) |
| **HDAC5** | ACHN | ENSG00000108840 | histone deacetylase 5 |
| **HECTD4** | SH-SY5Y | ENSG00000173064 | HECT domain containing E3 ubiquitin protein ligase 4 |
| **HELLS** | SH-SY5Y | ENSG00000119969 | helicase, lymphoid-specific |
| **HELQ** | ACHN | ENSG00000163312 | helicase, POLQ-like |
| **HELZ2** | HeLa-S3 | ENSG00000130589 | helicase with zinc finger 2, transcriptional coactivator |
| **HEPACAM** | HepG2 | ENSG00000165478 | hepatic and glial cell adhesion molecule |
| **HERC1** | SH-SY5Y | ENSG00000103657 | HECT and RLD domain containing E3 ubiquitin protein ligase family member 1 |
| **HERC2** | SH-SY5Y | ENSG00000128731 | HECT and RLD domain containing E3 ubiquitin protein ligase 2 |
| **HERC2P2** | SH-SY5Y | ENSG00000140181 | hect domain and RLD 2 pseudogene 2 |
| **HES1** | ACHN | ENSG00000114315 | hairy and enhancer of split 1, (Drosophila) |
| **HES6** | HepG2 | ENSG00000144485 | hairy and enhancer of split 6 (Drosophila) |
| **HGF** | SH-SY5Y | ENSG00000019991 | hepatocyte growth factor (hepapoietin A; scatter factor) |
| **HHEX** | HepG2 | ENSG00000152804 | hematopoietically expressed homeobox |
| **HIC1** | SH-SY5Y | ENSG00000177374 | hypermethylated in cancer 1 |
| **HIF1A** | U-87 MG | ENSG00000100644 | hypoxia inducible factor 1, alpha subunit (basic helix-loop-helix transcription factor) |
| **HIST1H2AC** | ACHN | ENSG00000180573 | histone cluster 1, H2ac |
| **HIST1H4H** | ACHN | ENSG00000158406 | histone cluster 1, H4h |
| **HJURP** | SH-SY5Y | ENSG00000123485 | Holliday junction recognition protein |
| **HLA-E** | ACHN | ENSG00000204592 | major histocompatibility complex, class I, E |
| **HMGA2** | ACHN | ENSG00000149948 | high mobility group AT-hook 2 |
| **HMGB1** | SH-SY5Y | ENSG00000189403 | high mobility group box 1 |
| **HMGCS1** | HepG2 | ENSG00000112972 | 3-hydroxy-3-methylglutaryl-CoA synthase 1 (soluble) |
| **HMGXB3** | SH-SY5Y | ENSG00000113716 | HMG box domain containing 3 |
| **HMMR** | ACHN | ENSG00000072571 | hyaluronan-mediated motility receptor (RHAMM) |
| **HNRNPU-AS1** | SH-SY5Y | ENSG00000188206 | HNRNPU antisense RNA 1 |
| **HOXD-AS1** | HepG2 | ENSG00000224189 | HOXD cluster antisense RNA 1 |
| **HP** | HepG2 | ENSG00000257017 | haptoglobin |
| **HPCAL1** | hFOB1.19 | ENSG00000115756 | hippocalcin-like 1 |
| **HPN** | HepG2 | ENSG00000105707 | hepsin |
| **HPS1** | ACHN | ENSG00000107521 | Hermansky-Pudlak syndrome 1 |
| **HS3ST3B1** | HepG2 | ENSG00000125430 | heparan sulfate (glucosamine) 3-O-sulfotransferase 3B1 |
| **HS6ST1** | HepG2 | ENSG00000136720 | heparan sulfate 6-O-sulfotransferase 1 |
| **HS6ST3** | SH-SY5Y | ENSG00000185352 | heparan sulfate 6-O-sulfotransferase 3 |
| **HSD17B7** | ACHN | ENSG00000132196 | hydroxysteroid (17-beta) dehydrogenase 7 |
| **HSPA12A** | HepG2 | ENSG00000165868 | heat shock 70kDa protein 12A |
| **HSPA1A** | SH-SY5Y | ENSG00000204389 | heat shock 70kDa protein 1A |
| **HSPA1B** | U-87 MG | ENSG00000204388 | heat shock 70kDa protein 1B |
| **HSPA4L** | ACHN | ENSG00000164070 | heat shock 70kDa protein 4-like |
| **HSPA5** | SH-SY5Y | ENSG00000044574 | heat shock 70kDa protein 5 (glucose-regulated protein, 78kDa) |
| **HSPA8** | U-87 MG | ENSG00000109971 | heat shock 70kDa protein 8 |
| **HSPA8P1** | U-87 MG | ENSG00000234176 | heat shock 70kDa protein 8 pseudogene 1 |
| **HSPA8P5** | U-87 MG | ENSG00000256356 | heat shock 70kDa protein 8 pseudogene 5 |
| **HSPA8P8** | U-87 MG | ENSG00000229091 | heat shock 70kDa protein 8 pseudogene 8 |
| **HSPB1** | IMR-32 | ENSG00000106211 | heat shock 27kDa protein 1 |
| **HSPB8** | HepG2 | ENSG00000152137 | heat shock 22kDa protein 8 |
| **HSPG2** | SH-SY5Y | ENSG00000142798 | heparan sulfate proteoglycan 2 |
| **HUNK** | SH-SY5Y | ENSG00000142149 | hormonally up-regulated Neu-associated kinase |
| **ICK** | HepG2 | ENSG00000112144 | intestinal cell (MAK-like) kinase |
| **ICOSLG** | ACHN | ENSG00000160223 | inducible T-cell co-stimulator ligand |
| **ID1** | U-87 MG | ENSG00000125968 | inhibitor of DNA binding 1, dominant negative helix-loop-helix protein |
| **ID2** | SH-SY5Y | ENSG00000115738 | inhibitor of DNA binding 2, dominant negative helix-loop-helix protein |
| **IDH1** | ACHN | ENSG00000138413 | isocitrate dehydrogenase 1 (NADP+), soluble |
| **IDH2** | SH-SY5Y | ENSG00000182054 | isocitrate dehydrogenase 2 (NADP+), mitochondrial |
| **IDS** | HepG2 | ENSG00000010404 | iduronate 2-sulfatase |
| **IER3** | SH-SY5Y | ENSG00000137331 | immediate early response 3 |
| **IFIT2** | ACHN | ENSG00000119922 | interferon-induced protein with tetratricopeptide repeats 2 |
| **IFIT5** | ACHN | ENSG00000152778 | interferon-induced protein with tetratricopeptide repeats 5 |
| **IFITM10** | SH-SY5Y | ENSG00000244242 | interferon induced transmembrane protein 10 |
| **IFNB1** | hFOB1.19 | ENSG00000171855 | interferon, beta 1, fibroblast |
| **IFRD1** | SH-SY5Y | ENSG00000006652 | interferon-related developmental regulator 1 |
| **IGF1R** | SH-SY5Y | ENSG00000140443 | insulin-like growth factor 1 receptor |
| **IGFBP1** | HepG2 | ENSG00000146678 | insulin-like growth factor binding protein 1 |
| **IGFBP4** | HepG2 | ENSG00000141753 | insulin-like growth factor binding protein 4 |
| **IGFBP5** | IMR-32 | ENSG00000115461 | insulin-like growth factor binding protein 5 |
| **IGFBP7** | hFOB1.19 | ENSG00000163453 | insulin-like growth factor binding protein 7 |
| **IGSF1** | HepG2 | ENSG00000147255 | immunoglobulin superfamily, member 1 |
| **IGSF3** | SH-SY5Y | ENSG00000143061 | immunoglobulin superfamily, member 3 |
| **IGSF9B** | SH-SY5Y | ENSG00000080854 | immunoglobulin superfamily, member 9B |
| **IKBKE** | ACHN | ENSG00000143466 | inhibitor of kappa light polypeptide gene enhancer in B-cells, kinase epsilon |
| **IL11RA** | SH-SY5Y | ENSG00000137070 | interleukin 11 receptor, alpha |
| **IL16** | U-87 MG | ENSG00000172349 | interleukin 16 |
| **IL18** | HepG2 | ENSG00000150782 | interleukin 18 (interferon-gamma-inducing factor) |
| **IL1B** | hFOB1.19 | ENSG00000125538 | interleukin 1, beta |
| **IL20RA** | SH-SY5Y | ENSG00000016402 | interleukin 20 receptor, alpha |
| **IL21R** | HepG2 | ENSG00000103522 | interleukin 21 receptor |
| **IL32** | ACHN | ENSG00000008517 | interleukin 32 |
| **IL4I1** | ACHN | ENSG00000104951 | interleukin 4 induced 1 |
| **IMPA2** | HepG2 | ENSG00000141401 | inositol(myo)-1(or 4)-monophosphatase 2 |
| **INA** | ACHN | ENSG00000148798 | internexin neuronal intermediate filament protein, alpha |
| **INHBA** | hFOB1.19 | ENSG00000122641 | inhibin, beta A |
| **INHBE** | HepG2 | ENSG00000139269 | inhibin, beta E |
| **INPP5F** | HepG2 | ENSG00000198825 | inositol polyphosphate-5-phosphatase F |
| **INSIG1** | U-87 MG | ENSG00000186480 | insulin induced gene 1 |
| **INTS6P1** | SH-SY5Y | ENSG00000250492 | integrator complex subunit 6 pseudogene 1 |
| **IP6K2** | ACHN | ENSG00000068745 | inositol hexakisphosphate kinase 2 |
| **IP6K3** | HepG2 | ENSG00000161896 | inositol hexakisphosphate kinase 3 |
| **IQGAP2** | SH-SY5Y | ENSG00000145703 | IQ motif containing GTPase activating protein 2 |
| **IQGAP3** | SH-SY5Y | ENSG00000183856 | IQ motif containing GTPase activating protein 3 |
| **IQSEC2** | HepG2 | ENSG00000124313 | IQ motif and Sec7 domain 2 |
| **IQUB** | SH-SY5Y | ENSG00000164675 | IQ motif and ubiquitin domain containing |
| **IRX3** | hFOB1.19 | ENSG00000177508 | iroquois homeobox 3 |
| **ISG15** | ACHN | ENSG00000187608 | ISG15 ubiquitin-like modifier |
| **ISLR** | IMR-32 | ENSG00000129009 | immunoglobulin superfamily containing leucine-rich repeat |
| **ISLR2** | IMR-32 | ENSG00000167178 | immunoglobulin superfamily containing leucine-rich repeat 2 |
| **ISM1** | HepG2 | ENSG00000101230 | isthmin 1 homolog (zebrafish) |
| **ITGA3** | HepG2 | ENSG00000005884 | integrin, alpha 3 (antigen CD49C, alpha 3 subunit of VLA-3 receptor) |
| **ITGA5** | U-87 MG | ENSG00000161638 | integrin, alpha 5 (fibronectin receptor, alpha polypeptide) |
| **JAG1** | SK-N-SH | ENSG00000101384 | jagged 1 |
| **JAK1** | SH-SY5Y | ENSG00000162434 | Janus kinase 1 |
| **JUN** | SH-SY5Y | ENSG00000177606 | jun proto-oncogene |
| **KAL1** | hFOB1.19 | ENSG00000011201 | Kallmann syndrome 1 sequence |
| **KANSL1L** | ACHN | ENSG00000144445 | KAT8 regulatory NSL complex subunit 1-like |
| **KCNB1** | SH-SY5Y | ENSG00000158445 | potassium voltage-gated channel, Shab-related subfamily, member 1 |
| **KCNJ10** | HepG2 | ENSG00000177807 | potassium inwardly-rectifying channel, subfamily J, member 10 |
| **KCNN4** | HepG2 | ENSG00000104783 | potassium intermediate/small conductance calcium-activated channel, subfamily N, member 4 |
| **KCNQ3** | hFOB1.19 | ENSG00000184156 | potassium voltage-gated channel, KQT-like subfamily, member 3 |
| **KCNT1** | SH-SY5Y | ENSG00000107147 | potassium channel, subfamily T, member 1 |
| **KCTD11** | HepG2 | ENSG00000213859 | potassium channel tetramerisation domain containing 11 |
| **KCTD12** | SH-SY5Y | ENSG00000178695 | potassium channel tetramerisation domain containing 12 |
| **KDM3A** | HepG2 | ENSG00000115548 | lysine (K)-specific demethylase 3A |
| **KDM4C** | SH-SY5Y | ENSG00000107077 | lysine (K)-specific demethylase 4C |
| **KDM5C** | HepG2 | ENSG00000126012 | lysine (K)-specific demethylase 5C |
| **KDM6B** | ACHN | ENSG00000132510 | lysine (K)-specific demethylase 6B |
| **KIAA0146** | SH-SY5Y | ENSG00000164808 | KIAA0146 |
| **KIAA0513** | ACHN | ENSG00000135709 | KIAA0513 |
| **KIAA0586** | ACHN | ENSG00000100578 | KIAA0586 |
| **KIAA0753** | ACHN | ENSG00000198920 | KIAA0753 |
| **KIAA0754** | SH-SY5Y | ENSG00000255103 | KIAA0754 |
| **KIAA1462** | ACHN | ENSG00000165757 | KIAA1462 |
| **KIAA1549** | SH-SY5Y | ENSG00000122778 | KIAA1549 |
| **KIAA1549L** | SH-SY5Y | ENSG00000110427 | KIAA1549-like |
| **KIAA1614** | SH-SY5Y | ENSG00000135835 | KIAA1614 |
| **KIAA1958** | SH-SY5Y | ENSG00000165185 | KIAA1958 |
| **KIF11** | SH-SY5Y | ENSG00000138160 | kinesin family member 11 |
| **KIF14** | SH-SY5Y | ENSG00000118193 | kinesin family member 14 |
| **KIF15** | SH-SY5Y | ENSG00000163808 | kinesin family member 15 |
| **KIF24** | ACHN | ENSG00000186638 | kinesin family member 24 |
| **KIF2C** | SH-SY5Y | ENSG00000142945 | kinesin family member 2C |
| **KIF4A** | SH-SY5Y | ENSG00000090889 | kinesin family member 4A |
| **KIF4B** | SH-SY5Y | ENSG00000226650 | kinesin family member 4B |
| **KIFC1** | SH-SY5Y | ENSG00000237649 | kinesin family member C1 |
| **KIRREL** | SH-SY5Y | ENSG00000183853 | kin of IRRE like (Drosophila) |
| **KIT** | hFOB1.19 | ENSG00000157404 | v-kit Hardy-Zuckerman 4 feline sarcoma viral oncogene homolog |
| **KL** | HepG2 | ENSG00000133116 | klotho |
| **KLB** | HepG2 | ENSG00000134962 | klotho beta |
| **KLF12** | U-87 MG | ENSG00000118922 | Kruppel-like factor 12 |
| **KLF15** | ACHN | ENSG00000163884 | Kruppel-like factor 15 |
| **KLF4** | hFOB1.19 | ENSG00000136826 | Kruppel-like factor 4 (gut) |
| **KLF7** | HepG2 | ENSG00000118263 | Kruppel-like factor 7 (ubiquitous) |
| **KLHDC7A** | ACHN | ENSG00000179023 | kelch domain containing 7A |
| **KLHL30** | ACHN | ENSG00000168427 | kelch-like family member 30 |
| **KLK6** | HepG2 | ENSG00000167755 | kallikrein-related peptidase 6 |
| **KNTC1** | SH-SY5Y | ENSG00000184445 | kinetochore associated 1 |
| **KPNA4** | SH-SY5Y | ENSG00000186432 | karyopherin alpha 4 (importin alpha 3) |
| **KRT37** | HepG2 | ENSG00000108417 | keratin 37 |
| **KRT7** | ACHN | ENSG00000135480 | keratin 7 |
| **KRT75** | U-2 Os | ENSG00000170454 | keratin 75 |
| **KRT80** | HepG2 | ENSG00000167767 | keratin 80 |
| **KRTAP2-3** | U-2 Os | ENSG00000212724 | keratin associated protein 2-3 |
| **LAPTM5** | HepG2 | ENSG00000162511 | lysosomal protein transmembrane 5 |
| **LATS2** | SH-SY5Y | ENSG00000150457 | large tumor suppressor kinase 2 |
| **LBR** | HepG2 | ENSG00000143815 | lamin B receptor |
| **LCP1** | SH-SY5Y | ENSG00000136167 | lymphocyte cytosolic protein 1 (L-plastin) |
| **LDHA** | SH-SY5Y | ENSG00000134333 | lactate dehydrogenase A |
| **LDHBP2** | ACHN | ENSG00000213684 | lactate dehydrogenase B pseudogene 2 |
| **LDLR** | SH-SY5Y | ENSG00000130164 | low density lipoprotein receptor |
| **LENG8** | U-87 MG | ENSG00000167615 | leukocyte receptor cluster (LRC) member 8 |
| **LFNG** | HeLa-S3 | ENSG00000106003 | LFNG O-fucosylpeptide 3-beta-N-acetylglucosaminyltransferase |
| **LGALS3** | SH-SY5Y | ENSG00000131981 | lectin, galactoside-binding, soluble, 3 |
| **LGR6** | SH-SY5Y | ENSG00000133067 | leucine-rich repeat containing G protein-coupled receptor 6 |
| **LIF** | HepG2 | ENSG00000128342 | leukemia inhibitory factor |
| **LIMCH1** | SH-SY5Y | ENSG00000064042 | LIM and calponin homology domains 1 |
| **LINC00176** | HepG2 | ENSG00000196421 | long intergenic non-protein coding RNA 176 |
| **LINC00265** | ACHN | ENSG00000188185 | long intergenic non-protein coding RNA 265 |
| **LINC00472** | ACHN | ENSG00000233237 | long intergenic non-protein coding RNA 472 |
| **LINC00511** | ACHN | ENSG00000227036 | long intergenic non-protein coding RNA 511 |
| **LINC00707** | HeLa-S3 | ENSG00000238266 | long intergenic non-protein coding RNA 707 |
| **LMCD1** | hFOB1.19 | ENSG00000071282 | LIM and cysteine-rich domains 1 |
| **LOXL2** | HepG2 | ENSG00000134013 | lysyl oxidase-like 2 |
| **LOXL3** | HepG2 | ENSG00000115318 | lysyl oxidase-like 3 |
| **LPAR1** | SH-SY5Y | ENSG00000198121 | lysophosphatidic acid receptor 1 |
| **LPCAT1** | U-87 MG | ENSG00000153395 | lysophosphatidylcholine acyltransferase 1 |
| **LPP** | HeLa-S3 | ENSG00000145012 | LIM domain containing preferred translocation partner in lipoma |
| **LPXN** | hFOB1.19 | ENSG00000110031 | leupaxin |
| **LRBA** | SH-SY5Y | ENSG00000198589 | LPS-responsive vesicle trafficking, beach and anchor containing |
| **LRP3** | HepG2 | ENSG00000130881 | low density lipoprotein receptor-related protein 3 |
| **LRRC2** | SH-SY5Y | ENSG00000163827 | leucine rich repeat containing 2 |
| **LRRN3** | hFOB1.19 | ENSG00000173114 | leucine rich repeat neuronal 3 |
| **LRRN4** | HepG2 | ENSG00000125872 | leucine rich repeat neuronal 4 |
| **LRRTM2** | SH-SY5Y | ENSG00000146006 | leucine rich repeat transmembrane neuronal 2 |
| **LTA** | SH-SY5Y | ENSG00000226979 | lymphotoxin alpha (TNF superfamily, member 1) |
| **LTBP2** | HepG2 | ENSG00000119681 | latent transforming growth factor beta binding protein 2 |
| **LUZP1** | U-87 MG | ENSG00000169641 | leucine zipper protein 1 |
| **LUZP2** | SH-SY5Y | ENSG00000187398 | leucine zipper protein 2 |
| **LYST** | U-87 MG | ENSG00000143669 | lysosomal trafficking regulator |
| **LZTS1** | HepG2 | ENSG00000061337 | leucine zipper, putative tumor suppressor 1 |
| **MAD2L2** | SH-SY5Y | ENSG00000116670 | MAD2 mitotic arrest deficient-like 2 (yeast) |
| **MAFG** | ACHN | ENSG00000197063 | v-maf musculoaponeurotic fibrosarcoma oncogene homolog G (avian) |
| **MAGI3** | SH-SY5Y | ENSG00000081026 | membrane associated guanylate kinase, WW and PDZ domain containing 3 |
| **MAL2** | HepG2 | ENSG00000147676 | mal, T-cell differentiation protein 2 (gene/pseudogene) |
| **MALSU1** | U-87 MG | ENSG00000156928 | mitochondrial assembly of ribosomal large subunit 1 |
| **MANSC1** | SH-SY5Y | ENSG00000111261 | MANSC domain containing 1 |
| **MAP1LC3B** | SH-SY5Y | ENSG00000140941 | microtubule-associated protein 1 light chain 3 beta |
| **MAP1LC3B2** | SH-SY5Y | ENSG00000171471 | microtubule-associated protein 1 light chain 3 beta 2 |
| **MAP2K3** | ACHN | ENSG00000034152 | mitogen-activated protein kinase kinase 3 |
| **MAPK4** | SH-SY5Y | ENSG00000141639 | mitogen-activated protein kinase 4 |
| **MAPK8IP3** | ACHN | ENSG00000138834 | mitogen-activated protein kinase 8 interacting protein 3 |
| **1-Mar** | HepG2 | ENSG00000186205 | mitochondrial amidoxime reducing component 1 |
| **3-Mar** | SH-SY5Y | ENSG00000173926 | membrane-associated ring finger (C3HC4) 3, E3 ubiquitin protein ligase |
| **7-Mar** | HepG2 | ENSG00000136536 | membrane-associated ring finger (C3HC4) 7, E3 ubiquitin protein ligase |
| **MARS2** | HepG2 | ENSG00000247626 | methionyl-tRNA synthetase 2, mitochondrial |
| **MAT1A** | HepG2 | ENSG00000151224 | methionine adenosyltransferase I, alpha |
| **MATN3** | HepG2 | ENSG00000132031 | matrilin 3 |
| **MB** | HepG2 | ENSG00000198125 | myoglobin |
| **MBIP** | ACHN | ENSG00000151332 | MAP3K12 binding inhibitory protein 1 |
| **MBNL2** | IMR-32 | ENSG00000139793 | muscleblind-like splicing regulator 2 |
| **MCAM** | HepG2 | ENSG00000076706 | melanoma cell adhesion molecule |
| **MCC** | HepG2 | ENSG00000171444 | mutated in colorectal cancers |
| **MCM10** | SH-SY5Y | ENSG00000065328 | minichromosome maintenance complex component 10 |
| **MCM6** | SH-SY5Y | ENSG00000076003 | minichromosome maintenance complex component 6 |
| **MCM7** | HepG2 | ENSG00000166508 | minichromosome maintenance complex component 7 |
| **MCOLN1** | ACHN | ENSG00000090674 | mucolipin 1 |
| **MDFI** | HepG2 | ENSG00000112559 | MyoD family inhibitor |
| **MDM4** | U-87 MG | ENSG00000198625 | Mdm4 p53 binding protein homolog (mouse) |
| **MDN1** | U-87 MG | ENSG00000112159 | MDN1, midasin homolog (yeast) |
| **ME1** | SH-SY5Y | ENSG00000065833 | malic enzyme 1, NADP(+)-dependent, cytosolic |
| **MED15** | ACHN | ENSG00000099917 | mediator complex subunit 15 |
| **MEF2D** | SH-SY5Y | ENSG00000116604 | myocyte enhancer factor 2D |
| **MEG3** | U-87 MG | ENSG00000214548 | maternally expressed 3 (non-protein coding) |
| **MEGF8** | SH-SY5Y | ENSG00000105429 | multiple EGF-like-domains 8 |
| **MELK** | SH-SY5Y | ENSG00000165304 | maternal embryonic leucine zipper kinase |
| **MEX3C** | HepG2 | ENSG00000176624 | mex-3 homolog C (*C. elegans*) |
| **MFAP2** | HeLa-S3 | ENSG00000117122 | microfibrillar-associated protein 2 |
| **MGAT4B** | SH-SY5Y | ENSG00000161013 | mannosyl (alpha-1,3-)-glycoprotein beta-1,4-N-acetylglucosaminyltransferase, isozyme B |
| **MGEA5** | U-87 MG | ENSG00000198408 | meningioma expressed antigen 5 (hyaluronidase) |
| **MICA** | ACHN | ENSG00000204520 | MHC class I polypeptide-related sequence A |
| **MICALL2** | ACHN | ENSG00000164877 | MICAL-like 2 |
| **MIR7-3HG** | SH-SY5Y | ENSG00000176840 | MIR7-3 host gene (non-protein coding) |
| **MITF** | ACHN | ENSG00000187098 | microphthalmia-associated transcription factor |
| **MIXL1** | HepG2 | ENSG00000185155 | Mix paired-like homeobox |
| **MKLN1** | U-87 MG | ENSG00000128585 | muskelin 1, intracellular mediator containing kelch motifs |
| **MLF1** | SH-SY5Y | ENSG00000178053 | myeloid leukemia factor 1 |
| **MLF1IP** | SH-SY5Y | ENSG00000151725 | MLF1 interacting protein |
| **MLL** | U-87 MG | ENSG00000118058 | myeloid/lymphoid or mixed-lineage leukemia (trithorax homolog, Drosophila) |
| **MLXIPL** | HepG2 | ENSG00000009950 | MLX interacting protein-like |
| **MMP19** | ACHN | ENSG00000123342 | matrix metallopeptidase 19 |
| **MMP25** | SH-SY5Y | ENSG00000008516 | matrix metallopeptidase 25 |
| **MMS22L** | SH-SY5Y | ENSG00000146263 | MMS22-like, DNA repair protein |
| **MOK** | hFOB1.19 | ENSG00000080823 | MOK protein kinase |
| **MPP6** | SH-SY5Y | ENSG00000105926 | membrane protein, palmitoylated 6 (MAGUK p55 subfamily member 6) |
| **MRPL39** | SH-SY5Y | ENSG00000154719 | mitochondrial ribosomal protein L39 |
| **MSI2** | SH-SY5Y | ENSG00000153944 | musashi RNA-binding protein 2 |
| **MSL1** | ACHN | ENSG00000188895 | male-specific lethal 1 homolog (Drosophila) |
| **MSX2** | SH-SY5Y | ENSG00000120149 | msh homeobox 2 |
| **MT-ND3** | ACHN | ENSG00000198840 | mitochondrially encoded NADH dehydrogenase 3 |
| **MT-RNR1** | HepG2 | ENSG00000211459 | mitochondrially encoded 12S RNA |
| **MT-TC** | ACHN | ENSG00000210140 | mitochondrially encoded tRNA cysteine |
| **MT-TP** | HepG2 | ENSG00000210196 | mitochondrially encoded tRNA proline |
| **MT1E** | ACHN | ENSG00000169715 | metallothionein 1E |
| **MT1G** | HepG2 | ENSG00000125144 | metallothionein 1G |
| **MT2P1** | HepG2 | ENSG00000162840 | metallothionein 2 pseudogene 1 |
| **MTHFR** | ACHN | ENSG00000177000 | methylenetetrahydrofolate reductase (NAD(P)H) |
| **MTMR10** | ACHN | ENSG00000166912 | myotubularin related protein 10 |
| **MTND4P12** | SH-SY5Y | ENSG00000247627 | MT-ND4 pseudogene 12 |
| **MTR** | SH-SY5Y | ENSG00000116984 | 5-methyltetrahydrofolate-homocysteine methyltransferase |
| **MTRNR2L10** | HepG2 | ENSG00000256045 | MT-RNR2-like 10 |
| **MTTP** | HepG2 | ENSG00000138823 | microsomal triglyceride transfer protein |
| **MUC5AC** | HepG2 | ENSG00000215182 | mucin 5AC, oligomeric mucus/gel-forming |
| **MVK** | ACHN | ENSG00000110921 | mevalonate kinase |
| **MXRA7** | HepG2 | ENSG00000182534 | matrix-remodelling associated 7 |
| **MYBPH** | HepG2 | ENSG00000133055 | myosin binding protein H |
| **MYEOV** | HepG2 | ENSG00000172927 | myeloma overexpressed |
| **MYH16** | HepG2 | ENSG00000002079 | myosin, heavy chain 16 pseudogene |
| **MYLK** | SH-SY5Y | ENSG00000065534 | myosin light chain kinase |
| **MYO1D** | SH-SY5Y | ENSG00000176658 | myosin ID |
| **MYOF** | HepG2 | ENSG00000138119 | myoferlin |
| **MYRIP** | SH-SY5Y | ENSG00000170011 | myosin VIIA and Rab interacting protein |
| **NADKD1** | HepG2 | ENSG00000152620 | NAD kinase domain containing 1 |
| **NAMPT** | U-87 MG | ENSG00000105835 | nicotinamide phosphoribosyltransferase |
| **NARS** | ACHN | ENSG00000134440 | asparaginyl-tRNA synthetase |
| **NARS2** | SH-SY5Y | ENSG00000137513 | asparaginyl-tRNA synthetase 2, mitochondrial (putative) |
| **NASP** | SH-SY5Y | ENSG00000132780 | nuclear autoantigenic sperm protein (histone-binding) |
| **NBEAL2** | SH-SY5Y | ENSG00000160796 | neurobeachin-like 2 |
| **NBPF3** | SH-SY5Y | ENSG00000142794 | neuroblastoma breakpoint family, member 3 |
| **NCAM1** | SH-SY5Y | ENSG00000149294 | neural cell adhesion molecule 1 |
| **NCAPD3** | SH-SY5Y | ENSG00000151503 | non-SMC condensin II complex, subunit D3 |
| **NCAPG** | SH-SY5Y | ENSG00000109805 | non-SMC condensin I complex, subunit G |
| **NCAPG2** | SH-SY5Y | ENSG00000146918 | non-SMC condensin II complex, subunit G2 |
| **NCLP1** | IMR-32 | ENSG00000213212 | nucleolin pseudogene 1 |
| **NCOR2** | IMR-32 | ENSG00000196498 | nuclear receptor corepressor 2 |
| **NEDD9** | SK-N-SH | ENSG00000111859 | neural precursor cell expressed, developmentally down-regulated 9 |
| **NET1** | ACHN | ENSG00000173848 | neuroepithelial cell transforming 1 |
| **NEU1** | ACHN | ENSG00000204386 | sialidase 1 (lysosomal sialidase) |
| **NEURL1B** | SH-SY5Y | ENSG00000214357 | neuralized homolog 1B (Drosophila) |
| **NEURL3** | ACHN | ENSG00000163121 | neuralized homolog 3 (Drosophila) pseudogene |
| **NEUROD1** | IMR-32 | ENSG00000162992 | neuronal differentiation 1 |
| **NFE2L3** | HepG2 | ENSG00000050344 | nuclear factor (erythroid-derived 2)-like 3 |
| **NFIA** | SH-SY5Y | ENSG00000162599 | nuclear factor I/A |
| **NFIB** | SH-SY5Y | ENSG00000147862 | nuclear factor I/B |
| **NFKB1** | ACHN | ENSG00000109320 | nuclear factor of kappa light polypeptide gene enhancer in B-cells 1 |
| **NFKB2** | ACHN | ENSG00000077150 | nuclear factor of kappa light polypeptide gene enhancer in B-cells 2 (p49/p100) |
| **NFKBIA** | ACHN | ENSG00000100906 | nuclear factor of kappa light polypeptide gene enhancer in B-cells inhibitor, alpha |
| **NFKBID** | ACHN | ENSG00000167604 | nuclear factor of kappa light polypeptide gene enhancer in B-cells inhibitor, delta |
| **NHLRC2** | U-87 MG | ENSG00000196865 | NHL repeat containing 2 |
| **NID1** | HepG2 | ENSG00000116962 | nidogen 1 |
| **NIN** | HepG2 | ENSG00000100503 | ninein (GSK3B interacting protein) |
| **NISCH** | ACHN | ENSG00000010322 | nischarin |
| **NLGN1** | SH-SY5Y | ENSG00000169760 | neuroligin 1 |
| **NLRP3** | U-2 Os | ENSG00000162711 | NLR family, pyrin domain containing 3 |
| **NMI** | ACHN | ENSG00000123609 | N-myc (and STAT) interactor |
| **NNAT** | SH-SY5Y | ENSG00000053438 | neuronatin |
| **NOP58** | SH-SY5Y | ENSG00000055044 | NOP58 ribonucleoprotein |
| **NPAS2** | ACHN | ENSG00000170485 | neuronal PAS domain protein 2 |
| **NPC1L1** | SH-SY5Y | ENSG00000015520 | NPC1-like 1 |
| **NPFFR2** | ACHN | ENSG00000056291 | neuropeptide FF receptor 2 |
| **NPNT** | HepG2 | ENSG00000168743 | nephronectin |
| **NPY** | SH-SY5Y | ENSG00000122585 | neuropeptide Y |
| **NR1H4** | HepG2 | ENSG00000012504 | nuclear receptor subfamily 1, group H, member 4 |
| **NR2F6** | ACHN | ENSG00000160113 | nuclear receptor subfamily 2, group F, member 6 |
| **NR4A2** | U-87 MG | ENSG00000153234 | nuclear receptor subfamily 4, group A, member 2 |
| **NR4A3** | U-87 MG | ENSG00000119508 | nuclear receptor subfamily 4, group A, member 3 |
| **NRCAM** | SH-SY5Y | ENSG00000091129 | neuronal cell adhesion molecule |
| **NRIP3** | SH-SY5Y | ENSG00000175352 | nuclear receptor interacting protein 3 |
| **NRP1** | ACHN | ENSG00000099250 | neuropilin 1 |
| **NUAK1** | HepG2 | ENSG00000074590 | NUAK family, SNF1-like kinase, 1 |
| **NUF2** | SH-SY5Y | ENSG00000143228 | NUF2, NDC80 kinetochore complex component, homolog (*S. cerevisiae*) |
| **NUP210** | SH-SY5Y | ENSG00000132182 | nucleoporin 210kDa |
| **NUSAP1** | SH-SY5Y | ENSG00000137804 | nucleolar and spindle associated protein 1 |
| **OASL** | ACHN | ENSG00000135114 | 2'-5'-oligoadenylate synthetase-like |
| **OBSCN** | IMR-32 | ENSG00000154358 | obscurin, cytoskeletal calmodulin and titin-interacting RhoGEF |
| **ODAM** | SH-SY5Y | ENSG00000109205 | odontogenic, ameloblast asssociated |
| **OIP5** | HepG2 | ENSG00000104147 | Opa interacting protein 5 |
| **OLFML2A** | SH-SY5Y | ENSG00000185585 | olfactomedin-like 2A |
| **OPTN** | HepG2 | ENSG00000123240 | optineurin |
| **ORC1** | SH-SY5Y | ENSG00000085840 | origin recognition complex, subunit 1 |
| **OSBPL10** | HepG2 | ENSG00000144645 | oxysterol binding protein-like 10 |
| **OSGIN1** | ACHN | ENSG00000140961 | oxidative stress induced growth inhibitor 1 |
| **OTP** | SH-SY5Y | ENSG00000171540 | orthopedia homeobox |
| **OTUD1** | SH-SY5Y | ENSG00000165312 | OTU domain containing 1 |
| **OVGP1** | SH-SY5Y | ENSG00000085465 | oviductal glycoprotein 1, 120kDa |
| **OVOL1** | HepG2 | ENSG00000172818 | ovo-like 1(Drosophila) |
| **OXTR** | hFOB1.19 | ENSG00000180914 | oxytocin receptor |
| **P4HA3** | HeLa-S3 | ENSG00000149380 | prolyl 4-hydroxylase, alpha polypeptide III |
| **PAEP** | HepG2 | ENSG00000122133 | progestagen-associated endometrial protein |
| **PAGR1** | U-87 MG | ENSG00000185928 | PAXIP1 associated glutamate-rich protein 1 |
| **PAH** | HepG2 | ENSG00000171759 | phenylalanine hydroxylase |
| **PAK1** | HepG2 | ENSG00000149269 | p21 protein (Cdc42/Rac)-activated kinase 1 |
| **PANK2** | ACHN | ENSG00000125779 | pantothenate kinase 2 |
| **PANK3** | ACHN | ENSG00000120137 | pantothenate kinase 3 |
| **PAPPA** | U-87 MG | ENSG00000182752 | pregnancy-associated plasma protein A, pappalysin 1 |
| **PAQR8** | SH-SY5Y | ENSG00000170915 | progestin and adipoQ receptor family member VIII |
| **PARP1** | SH-SY5Y | ENSG00000143799 | poly (ADP-ribose) polymerase 1 |
| **PARP1P1** | SH-SY5Y | ENSG00000227105 | poly (ADP-ribose) polymerase family, member 1 pseudogene 1 |
| **PARVA** | HepG2 | ENSG00000197702 | parvin, alpha |
| **PASK** | SH-SY5Y | ENSG00000115687 | PAS domain containing serine/threonine kinase |
| **PAX6** | HepG2 | ENSG00000007372 | paired box 6 |
| **PBK** | SH-SY5Y | ENSG00000168078 | PDZ binding kinase |
| **PCDH1** | HepG2 | ENSG00000156453 | protocadherin 1 |
| **PCLO** | SH-SY5Y | ENSG00000186472 | piccolo presynaptic cytomatrix protein |
| **PCSK9** | HepG2 | ENSG00000169174 | proprotein convertase subtilisin/kexin type 9 |
| **PDDC1** | HepG2 | ENSG00000177225 | Parkinson disease 7 domain containing 1 |
| **PDE4D** | SH-SY5Y | ENSG00000113448 | phosphodiesterase 4D, cAMP-specific |
| **PDE7B** | SH-SY5Y | ENSG00000171408 | phosphodiesterase 7B |
| **PDGFC** | HepG2 | ENSG00000145431 | platelet derived growth factor C |
| **PDGFRB** | U-2 Os | ENSG00000113721 | platelet-derived growth factor receptor, beta polypeptide |
| **PDIA4** | SH-SY5Y | ENSG00000155660 | protein disulfide isomerase family A, member 4 |
| **PDP1** | U-87 MG | ENSG00000164951 | pyruvate dehyrogenase phosphatase catalytic subunit 1 |
| **PEG3** | HepG2 | ENSG00000198300 | paternally expressed 3 |
| **PER2** | ACHN | ENSG00000132326 | period circadian clock 2 |
| **PFAS** | HepG2 | ENSG00000178921 | phosphoribosylformylglycinamidine synthase |
| **PGF** | ACHN | ENSG00000119630 | placental growth factor |
| **PGM1** | HepG2 | ENSG00000079739 | phosphoglucomutase 1 |
| **PHC3** | U-87 MG | ENSG00000173889 | polyhomeotic homolog 3 (Drosophila) |
| **PHF21A** | ACHN | ENSG00000135365 | PHD finger protein 21A |
| **PHGDH** | IMR-32 | ENSG00000092621 | phosphoglycerate dehydrogenase |
| **PHKG2** | ACHN | ENSG00000156873 | phosphorylase kinase, gamma 2 (testis) |
| **PHLDA2** | SH-SY5Y | ENSG00000181649 | pleckstrin homology-like domain, family A, member 2 |
| **PHLDB2** | IMR-32 | ENSG00000144824 | pleckstrin homology-like domain, family B, member 2 |
| **PI15** | SH-SY5Y | ENSG00000137558 | peptidase inhibitor 15 |
| **PI4KA** | ACHN | ENSG00000241973 | phosphatidylinositol 4-kinase, catalytic, alpha |
| **PI4KB** | ACHN | ENSG00000143393 | phosphatidylinositol 4-kinase, catalytic, beta |
| **PIDD** | ACHN | ENSG00000177595 | p53-induced death domain protein |
| **PIK3IP1** | ACHN | ENSG00000100100 | phosphoinositide-3-kinase interacting protein 1 |
| **PILRA** | ACHN | ENSG00000085514 | paired immunoglobin-like type 2 receptor alpha |
| **PILRB** | ACHN | ENSG00000121716 | paired immunoglobin-like type 2 receptor beta |
| **PIM3** | ACHN | ENSG00000198355 | pim-3 oncogene |
| **PIP4K2A** | HepG2 | ENSG00000150867 | phosphatidylinositol-5-phosphate 4-kinase, type II, alpha |
| **PIP5K1A** | ACHN | ENSG00000143398 | phosphatidylinositol-4-phosphate 5-kinase, type I, alpha |
| **PISD** | ACHN | ENSG00000241878 | phosphatidylserine decarboxylase |
| **PKIA** | SH-SY5Y | ENSG00000171033 | protein kinase (cAMP-dependent, catalytic) inhibitor alpha |
| **PLA2G2A** | HepG2 | ENSG00000188257 | phospholipase A2, group IIA (platelets, synovial fluid) |
| **PLA2G6** | ACHN | ENSG00000184381 | phospholipase A2, group VI (cytosolic, calcium-independent) |
| **PLAC8** | HepG2 | ENSG00000145287 | placenta-specific 8 |
| **PLB1** | HepG2 | ENSG00000163803 | phospholipase B1 |
| **PLCB1** | HepG2 | ENSG00000182621 | phospholipase C, beta 1 (phosphoinositide-specific) |
| **PLCD1** | ACHN | ENSG00000187091 | phospholipase C, delta 1 |
| **PLCE1** | SH-SY5Y | ENSG00000138193 | phospholipase C, epsilon 1 |
| **PLCH2** | HepG2 | ENSG00000149527 | phospholipase C, eta 2 |
| **PLCXD3** | SH-SY5Y | ENSG00000182836 | phosphatidylinositol-specific phospholipase C, X domain containing 3 |
| **PLD2** | SH-SY5Y | ENSG00000129219 | phospholipase D2 |
| **PLD6** | ACHN | ENSG00000179598 | phospholipase D family, member 6 |
| **PLEC** | HeLa-S3 | ENSG00000178209 | plectin |
| **PLEKHA2** | SH-SY5Y | ENSG00000169499 | pleckstrin homology domain containing, family A (phosphoinositide binding specific) member 2 |
| **PLEKHG4B** | hFOB1.19 | ENSG00000153404 | pleckstrin homology domain containing, family G (with RhoGef domain) member 4B |
| **PLEKHG6** | hFOB1.19 | ENSG00000008323 | pleckstrin homology domain containing, family G (with RhoGef domain) member 6 |
| **PLEKHH1** | SH-SY5Y | ENSG00000054690 | pleckstrin homology domain containing, family H (with MyTH4 domain) member 1 |
| **PLK1S1** | HepG2 | ENSG00000088970 | polo-like kinase 1 substrate 1 |
| **PLOD2** | IMR-32 | ENSG00000152952 | procollagen-lysine, 2-oxoglutarate 5-dioxygenase 2 |
| **PLXNA2** | SH-SY5Y | ENSG00000076356 | plexin A2 |
| **PLXNB2** | ACHN | ENSG00000196576 | plexin B2 |
| **PNISR** | U-87 MG | ENSG00000132424 | PNN-interacting serine/arginine-rich protein |
| **POFUT2** | ACHN | ENSG00000186866 | protein O-fucosyltransferase 2 |
| **POLA2** | SH-SY5Y | ENSG00000014138 | polymerase (DNA directed), alpha 2, accessory subunit |
| **POLD2** | SH-SY5Y | ENSG00000106628 | polymerase (DNA directed), delta 2, accessory subunit |
| **POLH** | ACHN | ENSG00000170734 | polymerase (DNA directed), eta |
| **POLR2J4** | ACHN | ENSG00000214783 | polymerase (RNA) II (DNA directed) polypeptide J4, pseudogene |
| **PPARD** | SK-N-SH | ENSG00000112033 | peroxisome proliferator-activated receptor delta |
| **PPARGC1A** | ACHN | ENSG00000109819 | peroxisome proliferator-activated receptor gamma, coactivator 1 alpha |
| **PPFIBP2** | HepG2 | ENSG00000166387 | PTPRF interacting protein, binding protein 2 (liprin beta 2) |
| **PPIF** | U-87 MG | ENSG00000108179 | peptidylprolyl isomerase F |
| **PPL** | ACHN | ENSG00000118898 | periplakin |
| **PPP1R10** | U-87 MG | ENSG00000204569 | protein phosphatase 1, regulatory subunit 10 |
| **PPP1R13B** | IMR-32 | ENSG00000088808 | protein phosphatase 1, regulatory subunit 13B |
| **PPP1R16B** | SH-SY5Y | ENSG00000101445 | protein phosphatase 1, regulatory subunit 16B |
| **PPP1R3C** | ACHN | ENSG00000119938 | protein phosphatase 1, regulatory subunit 3C |
| **PPP2R2B** | SH-SY5Y | ENSG00000156475 | protein phosphatase 2, regulatory subunit B, beta |
| **PPP2R2C** | HepG2 | ENSG00000074211 | protein phosphatase 2, regulatory subunit B, gamma |
| **PPP4R4** | ACHN | ENSG00000119698 | protein phosphatase 4, regulatory subunit 4 |
| **PRDX1** | ACHN | ENSG00000117450 | peroxiredoxin 1 |
| **PRF1** | HepG2 | ENSG00000180644 | perforin 1 (pore forming protein) |
| **PRIMA1** | HepG2 | ENSG00000175785 | proline rich membrane anchor 1 |
| **PRKCA** | SH-SY5Y | ENSG00000154229 | protein kinase C, alpha |
| **PRKDC** | SH-SY5Y | ENSG00000253729 | protein kinase, DNA-activated, catalytic polypeptide |
| **PROS1** | SH-SY5Y | ENSG00000184500 | protein S (alpha) |
| **PROX1** | SH-SY5Y | ENSG00000117707 | prospero homeobox 1 |
| **PRR11** | SH-SY5Y | ENSG00000068489 | proline rich 11 |
| **PRR14** | ACHN | ENSG00000156858 | proline rich 14 |
| **PRR7** | SH-SY5Y | ENSG00000131188 | proline rich 7 (synaptic) |
| **PRSS12** | SH-SY5Y | ENSG00000164099 | protease, serine, 12 (neurotrypsin, motopsin) |
| **PRSS22** | HepG2 | ENSG00000005001 | protease, serine, 22 |
| **PSAT1P3** | U-87 MG | ENSG00000230787 | phosphoserine aminotransferase 1 pseudogene 3 |
| **PSG1** | HepG2 | ENSG00000231924 | pregnancy specific beta-1-glycoprotein 1 |
| **PSPC1** | SH-SY5Y | ENSG00000121390 | paraspeckle component 1 |
| **PSRC1** | SH-SY5Y | ENSG00000134222 | proline/serine-rich coiled-coil 1 |
| **PTAFR** | HepG2 | ENSG00000169403 | platelet-activating factor receptor |
| **PTGER2** | SH-SY5Y | ENSG00000125384 | prostaglandin E receptor 2 (subtype EP2), 53kDa |
| **PTGES3L-AARSD1** | ACHN | ENSG00000108825 | PTGES3L-AARSD1 readthrough |
| **PTK7** | U-87 MG | ENSG00000112655 | protein tyrosine kinase 7 |
| **PTMAP5** | SH-SY5Y | ENSG00000214182 | prothymosin, alpha pseudogene 5 |
| **PTN** | SH-SY5Y | ENSG00000105894 | pleiotrophin |
| **PTP4A3** | HepG2 | ENSG00000184489 | protein tyrosine phosphatase type IVA, member 3 |
| **PTPN14** | U-87 MG | ENSG00000152104 | protein tyrosine phosphatase, non-receptor type 14 |
| **PTPRU** | U-2 Os | ENSG00000060656 | protein tyrosine phosphatase, receptor type, U |
| **PTRF** | SH-SY5Y | ENSG00000177469 | polymerase I and transcript release factor |
| **PVT1** | ACHN | ENSG00000249859 | Pvt1 oncogene (non-protein coding) |
| **PXDN** | SH-SY5Y | ENSG00000130508 | peroxidasin homolog (Drosophila) |
| **PYGM** | SH-SY5Y | ENSG00000068976 | phosphorylase, glycogen, muscle |
| **RAB3B** | HepG2 | ENSG00000169213 | RAB3B, member RAS oncogene family |
| **RAC2** | HepG2 | ENSG00000128340 | ras-related C3 botulinum toxin substrate 2 (rho family, small GTP binding protein Rac2) |
| **RAD51** | SH-SY5Y | ENSG00000051180 | RAD51 homolog (*S. cerevisiae*) |
| **RAD51AP1** | HepG2 | ENSG00000111247 | RAD51 associated protein 1 |
| **RAD54L** | HepG2 | ENSG00000085999 | RAD54-like (*S. cerevisiae*) |
| **RAD9A** | ACHN | ENSG00000172613 | RAD9 homolog A (*S. pombe*) |
| **RAI14** | ACHN | ENSG00000039560 | retinoic acid induced 14 |
| **RAP1GAP2** | SH-SY5Y | ENSG00000132359 | RAP1 GTPase activating protein 2 |
| **RASAL2** | U-87 MG | ENSG00000075391 | RAS protein activator like 2 |
| **RASSF2** | ACHN | ENSG00000101265 | Ras association (RalGDS/AF-6) domain family member 2 |
| **RASSF5** | HepG2 | ENSG00000136653 | Ras association (RalGDS/AF-6) domain family member 5 |
| **RBL1** | SH-SY5Y | ENSG00000080839 | retinoblastoma-like 1 (p107) |
| **RBMS2** | HepG2 | ENSG00000076067 | RNA binding motif, single stranded interacting protein 2 |
| **RBMS3** | SH-SY5Y | ENSG00000144642 | RNA binding motif, single stranded interacting protein 3 |
| **RBP4** | HepG2 | ENSG00000138207 | retinol binding protein 4, plasma |
| **RCN1** | HepG2 | ENSG00000049449 | reticulocalbin 1, EF-hand calcium binding domain |
| **RD3** | SH-SY5Y | ENSG00000198570 | retinal degeneration 3 |
| **REL** | ACHN | ENSG00000162924 | v-rel reticuloendotheliosis viral oncogene homolog (avian) |
| **RERG** | SH-SY5Y | ENSG00000134533 | RAS-like, estrogen-regulated, growth inhibitor |
| **RET** | SH-SY5Y | ENSG00000165731 | ret proto-oncogene |
| **RFC3** | HepG2 | ENSG00000133119 | replication factor C (activator 1) 3, 38kDa |
| **RFX1** | ACHN | ENSG00000132005 | regulatory factor X, 1 (influences HLA class II expression) |
| **RGL1** | ACHN | ENSG00000143344 | ral guanine nucleotide dissociation stimulator-like 1 |
| **RHBDF1** | ACHN | ENSG00000007384 | rhomboid 5 homolog 1 (Drosophila) |
| **RHOBTB1** | HepG2 | ENSG00000072422 | Rho-related BTB domain containing 1 |
| **RHOBTB3** | HepG2 | ENSG00000164292 | Rho-related BTB domain containing 3 |
| **RIN2** | HepG2 | ENSG00000132669 | Ras and Rab interactor 2 |
| **RNASEH2A** | SH-SY5Y | ENSG00000104889 | ribonuclease H2, subunit A |
| **RND1** | hFOB1.19 | ENSG00000172602 | Rho family GTPase 1 |
| **RNF10** | SH-SY5Y | ENSG00000022840 | ring finger protein 10 |
| **RNF144B** | ACHN | ENSG00000137393 | ring finger protein 144B |
| **RNF19B** | SH-SY5Y | ENSG00000116514 | ring finger protein 19B |
| **RORB** | SH-SY5Y | ENSG00000198963 | RAR-related orphan receptor B |
| **RP11-121L10.3** | SH-SY5Y | ENSG00000214391 | NA |
| **RP11-12M5.3** | SH-SY5Y | ENSG00000229407 | NA |
| **RP11-145A3.1** | HepG2 | ENSG00000227496 | NA |
| **RP11-150O12.1** | SH-SY5Y | ENSG00000253161 | NA |
| **RP11-18C24.6** | ACHN | ENSG00000248008 | NA |
| **RP11-247C2.2** | IMR-32 | ENSG00000248540 | HCG2004779; Uncharacterized protein |
| **RP11-290F20.1** | SH-SY5Y | ENSG00000203999 | NA |
| **RP11-347C12.2** | SH-SY5Y | ENSG00000183604 | NA |
| **RP11-347H15.2** | SH-SY5Y | ENSG00000255500 | NA |
| **RP11-389O22.4** | HepG2 | ENSG00000233839 | NA |
| **RP11-424C20.2** | SH-SY5Y | ENSG00000256663 | NA |
| **RP11-467L13.5** | HeLa-S3 | ENSG00000223722 | NA |
| **RP11-4K3__A.5** | U-87 MG | ENSG00000241547 | NA |
| **RP11-512M8.3** | ACHN | ENSG00000256304 | NA |
| **RP11-564D11.3** | SH-SY5Y | ENSG00000255624 | NA |
| **RP11-706J10.1** | hFOB1.19 | ENSG00000254300 | NA |
| **RP11-83M16.3** | SH-SY5Y | ENSG00000250669 | NA |
| **RP11-875O11.2** | HepG2 | ENSG00000246130 | NA |
| **RP11-875O11.3** | ACHN | ENSG00000253616 | NA |
| **RP4-564F22.2** | SH-SY5Y | ENSG00000196756 | NA |
| **RPH3A** | SH-SY5Y | ENSG00000089169 | rabphilin 3A homolog (mouse) |
| **RPL23P2** | ACHN | ENSG00000176054 | ribosomal protein L23 pseudogene 2 |
| **RPLP0P2** | HepG2 | ENSG00000243742 | ribosomal protein, large, P0 pseudogene 2 |
| **RPS6KA1** | ACHN | ENSG00000117676 | ribosomal protein S6 kinase, 90kDa, polypeptide 1 |
| **RRM1** | SH-SY5Y | ENSG00000167325 | ribonucleotide reductase M1 |
| **RRM2P3** | SH-SY5Y | ENSG00000214018 | ribonucleotide reductase M2 polypeptide pseudogene 3 |
| **RSAD2** | ACHN | ENSG00000134321 | radical S-adenosyl methionine domain containing 2 |
| **RSF1** | U-87 MG | ENSG00000048649 | remodeling and spacing factor 1 |
| **RSL1D1** | SH-SY5Y | ENSG00000171490 | ribosomal L1 domain containing 1 |
| **RSPO3** | hFOB1.19 | ENSG00000146374 | R-spondin 3 |
| **RTN1** | SH-SY5Y | ENSG00000139970 | reticulon 1 |
| **RUNX1T1** | SH-SY5Y | ENSG00000079102 | runt-related transcription factor 1; translocated to, 1 (cyclin D-related) |
| **S100A11** | HepG2 | ENSG00000163191 | S100 calcium binding protein A11 |
| **S100A2** | HepG2 | ENSG00000196754 | S100 calcium binding protein A2 |
| **S100A3** | ACHN | ENSG00000188015 | S100 calcium binding protein A3 |
| **SAMD1** | U-87 MG | ENSG00000141858 | sterile alpha motif domain containing 1 |
| **SAMD11** | IMR-32 | ENSG00000187634 | sterile alpha motif domain containing 11 |
| **SAMD4A** | HepG2 | ENSG00000020577 | sterile alpha motif domain containing 4A |
| **SARS** | SH-SY5Y | ENSG00000031698 | seryl-tRNA synthetase |
| **SBNO2** | ACHN | ENSG00000064932 | strawberry notch homolog 2 (Drosophila) |
| **SCARA3** | SH-SY5Y | ENSG00000168077 | scavenger receptor class A, member 3 |
| **SCARF2** | SH-SY5Y | ENSG00000244486 | scavenger receptor class F, member 2 |
| **SCD** | SH-SY5Y | ENSG00000099194 | stearoyl-CoA desaturase (delta-9-desaturase) |
| **SCG3** | SH-SY5Y | ENSG00000104112 | secretogranin III |
| **SCN3A** | ACHN | ENSG00000153253 | sodium channel, voltage-gated, type III, alpha subunit |
| **SCNM1** | SH-SY5Y | ENSG00000163156 | sodium channel modifier 1 |
| **SCUBE1** | SH-SY5Y | ENSG00000159307 | signal peptide, CUB domain, EGF-like 1 |
| **SCYL2** | HepG2 | ENSG00000136021 | SCY1-like 2 (*S. cerevisiae*) |
| **SDK1** | SH-SY5Y | ENSG00000146555 | sidekick cell adhesion molecule 1 |
| **SDPR** | ACHN | ENSG00000168497 | serum deprivation response |
| **SEMA4A** | ACHN | ENSG00000196189 | sema domain, immunoglobulin domain (Ig), transmembrane domain (TM) and short cytoplasmic domain, (semaphorin) 4A |
| **SEMA4G** | HepG2 | ENSG00000095539 | sema domain, immunoglobulin domain (Ig), transmembrane domain (TM) and short cytoplasmic domain, (semaphorin) 4G |
| **SEMA5B** | SH-SY5Y | ENSG00000082684 | sema domain, seven thrombospondin repeats (type 1 and type 1-like), transmembrane domain (TM) and short cytoplasmic domain, (semaphorin) 5B |
| **SEMA6B** | U-2 Os | ENSG00000167680 | sema domain, transmembrane domain (TM), and cytoplasmic domain, (semaphorin) 6B |
| **SEPN1** | SH-SY5Y | ENSG00000162430 | selenoprotein N, 1 |
| **SEPP1** | HepG2 | ENSG00000250722 | selenoprotein P, plasma, 1 |
| **SERINC2** | HepG2 | ENSG00000168528 | serine incorporator 2 |
| **SERPINA1** | SH-SY5Y | ENSG00000197249 | serpin peptidase inhibitor, clade A (alpha-1 antiproteinase, antitrypsin), member 1 |
| **SERPINA3** | HepG2 | ENSG00000196136 | serpin peptidase inhibitor, clade A (alpha-1 antiproteinase, antitrypsin), member 3 |
| **SERPINA7** | HepG2 | ENSG00000123561 | serpin peptidase inhibitor, clade A (alpha-1 antiproteinase, antitrypsin), member 7 |
| **SERPINB2** | hFOB1.19 | ENSG00000197632 | serpin peptidase inhibitor, clade B (ovalbumin), member 2 |
| **SERPINB9** | ACHN | ENSG00000170542 | serpin peptidase inhibitor, clade B (ovalbumin), member 9 |
| **SERPINC1** | HepG2 | ENSG00000117601 | serpin peptidase inhibitor, clade C (antithrombin), member 1 |
| **SERPINE1** | SH-SY5Y | ENSG00000106366 | serpin peptidase inhibitor, clade E (nexin, plasminogen activator inhibitor type 1), member 1 |
| **SERPINF2** | HepG2 | ENSG00000167711 | serpin peptidase inhibitor, clade F (alpha-2 antiplasmin, pigment epithelium derived factor), member 2 |
| **SERTAD2** | U-87 MG | ENSG00000179833 | SERTA domain containing 2 |
| **SESTD1** | SH-SY5Y | ENSG00000187231 | SEC14 and spectrin domains 1 |
| **SETBP1** | HepG2 | ENSG00000152217 | SET binding protein 1 |
| **SEZ6L** | SH-SY5Y | ENSG00000100095 | seizure related 6 homolog (mouse)-like |
| **SFMBT2** | SH-SY5Y | ENSG00000198879 | Scm-like with four mbt domains 2 |
| **SFRP1** | SH-SY5Y | ENSG00000104332 | secreted frizzled-related protein 1 |
| **SGK1** | SH-SY5Y | ENSG00000118515 | serum/glucocorticoid regulated kinase 1 |
| **SGOL2** | SH-SY5Y | ENSG00000163535 | shugoshin-like 2 (*S. pombe*) |
| **SGSH** | HepG2 | ENSG00000181523 | N-sulfoglucosamine sulfohydrolase |
| **SH3BGRL3** | HepG2 | ENSG00000142669 | SH3 domain binding glutamic acid-rich protein like 3 |
| **SH3PXD2A** | SH-SY5Y | ENSG00000107957 | SH3 and PX domains 2A |
| **SH3RF2** | HepG2 | ENSG00000156463 | SH3 domain containing ring finger 2 |
| **SHCBP1** | SH-SY5Y | ENSG00000171241 | SHC SH2-domain binding protein 1 |
| **SHISA2** | ACHN | ENSG00000180730 | shisa homolog 2 (*Xenopus laevis*) |
| **SIGLEC15** | HepG2 | ENSG00000197046 | sialic acid binding Ig-like lectin 15 |
| **SIK3** | HepG2 | ENSG00000160584 | SIK family kinase 3 |
| **SIRT1** | SH-SY5Y | ENSG00000096717 | sirtuin 1 |
| **SKA3** | SH-SY5Y | ENSG00000165480 | spindle and kinetochore associated complex subunit 3 |
| **SKI** | HepG2 | ENSG00000157933 | v-ski sarcoma viral oncogene homolog (avian) |
| **SKIL** | SH-SY5Y | ENSG00000136603 | SKI-like oncogene |
| **SKP2** | HepG2 | ENSG00000145604 | S-phase kinase-associated protein 2, E3 ubiquitin protein ligase |
| **SLC12A4** | ACHN | ENSG00000124067 | solute carrier family 12 (potassium/chloride transporters), member 4 |
| **SLC12A6** | ACHN | ENSG00000140199 | solute carrier family 12 (potassium/chloride transporters), member 6 |
| **SLC12A7** | ACHN | ENSG00000113504 | solute carrier family 12 (potassium/chloride transporters), member 7 |
| **SLC13A3** | HepG2 | ENSG00000158296 | solute carrier family 13 (sodium-dependent dicarboxylate transporter), member 3 |
| **SLC13A5** | HepG2 | ENSG00000141485 | solute carrier family 13 (sodium-dependent citrate transporter), member 5 |
| **SLC16A13** | HepG2 | ENSG00000174327 | solute carrier family 16, member 13 (monocarboxylic acid transporter 13) |
| **SLC18A1** | SH-SY5Y | ENSG00000036565 | solute carrier family 18 (vesicular monoamine), member 1 |
| **SLC18A3** | SH-SY5Y | ENSG00000187714 | solute carrier family 18 (vesicular acetylcholine), member 3 |
| **SLC19A1** | HepG2 | ENSG00000173638 | solute carrier family 19 (folate transporter), member 1 |
| **SLC1A1** | U-87 MG | ENSG00000106688 | solute carrier family 1 (neuronal/epithelial high affinity glutamate transporter, system Xag), member 1 |
| **SLC1A3** | U-2 Os | ENSG00000079215 | solute carrier family 1 (glial high affinity glutamate transporter), member 3 |
| **SLC25A25** | ACHN | ENSG00000148339 | solute carrier family 25 (mitochondrial carrier; phosphate carrier), member 25 |
| **SLC2A1** | hFOB1.19 | ENSG00000117394 | solute carrier family 2 (facilitated glucose transporter), member 1 |
| **SLC2A3** | HepG2 | ENSG00000059804 | solute carrier family 2 (facilitated glucose transporter), member 3 |
| **SLC2A9** | HepG2 | ENSG00000109667 | solute carrier family 2 (facilitated glucose transporter), member 9 |
| **SLC35C1** | ACHN | ENSG00000181830 | solute carrier family 35, member C1 |
| **SLC35F3** | SK-N-SH | ENSG00000183780 | solute carrier family 35, member F3 |
| **SLC37A4** | HepG2 | ENSG00000137700 | solute carrier family 37 (glucose-6-phosphate transporter), member 4 |
| **SLC38A2** | ACHN | ENSG00000134294 | solute carrier family 38, member 2 |
| **SLC38A3** | HepG2 | ENSG00000188338 | solute carrier family 38, member 3 |
| **SLC39A6** | ACHN | ENSG00000141424 | solute carrier family 39 (zinc transporter), member 6 |
| **SLC44A5** | SH-SY5Y | ENSG00000137968 | solute carrier family 44, member 5 |
| **SLC4A7** | U-87 MG | ENSG00000033867 | solute carrier family 4, sodium bicarbonate cotransporter, member 7 |
| **SLC4A8** | SH-SY5Y | ENSG00000050438 | solute carrier family 4, sodium bicarbonate cotransporter, member 8 |
| **SLC7A2** | SH-SY5Y | ENSG00000003989 | solute carrier family 7 (cationic amino acid transporter, y+ system), member 2 |
| **SLC7A5** | SH-SY5Y | ENSG00000103257 | solute carrier family 7 (amino acid transporter light chain, L system), member 5 |
| **SLC7A8** | U-87 MG | ENSG00000092068 | solute carrier family 7 (amino acid transporter light chain, L system), member 8 |
| **SLC9A8** | ACHN | ENSG00000197818 | solute carrier family 9, subfamily A (NHE8, cation proton antiporter 8), member 8 |
| **SLIT3** | SH-SY5Y | ENSG00000184347 | slit homolog 3 (Drosophila) |
| **SMAD5** | U-87 MG | ENSG00000113658 | SMAD family member 5 |
| **SMAD9** | HepG2 | ENSG00000120693 | SMAD family member 9 |
| **SMAP2** | U-87 MG | ENSG00000084070 | small ArfGAP2 |
| **SMC1A** | SH-SY5Y | ENSG00000072501 | structural maintenance of chromosomes 1A |
| **SMC2** | SH-SY5Y | ENSG00000136824 | structural maintenance of chromosomes 2 |
| **SMG1** | U-87 MG | ENSG00000157106 | smg-1 homolog, phosphatidylinositol 3-kinase-related kinase (*C. elegans*) |
| **SMIM3** | hFOB1.19 | ENSG00000256235 | small integral membrane protein 3 |
| **SMOC1** | U-87 MG | ENSG00000198732 | SPARC related modular calcium binding 1 |
| **SMURF2** | HepG2 | ENSG00000108854 | SMAD specific E3 ubiquitin protein ligase 2 |
| **SMYD3** | SH-SY5Y | ENSG00000185420 | SET and MYND domain containing 3 |
| **SNAPC4** | ACHN | ENSG00000165684 | small nuclear RNA activating complex, polypeptide 4, 190kDa |
| **SNHG12** | ACHN | ENSG00000197989 | small nucleolar RNA host gene 12 (non-protein coding) |
| **SNHG15** | SH-SY5Y | ENSG00000232956 | small nucleolar RNA host gene 15 (non-protein coding) |
| **SNRNP48** | SH-SY5Y | ENSG00000168566 | small nuclear ribonucleoprotein 48kDa (U11/U12) |
| **SOD2** | HepG2 | ENSG00000112096 | superoxide dismutase 2, mitochondrial |
| **SOGA1** | SH-SY5Y | ENSG00000149639 | suppressor of glucose, autophagy associated 1 |
| **SORBS1** | HepG2 | ENSG00000095637 | sorbin and SH3 domain containing 1 |
| **SORBS2** | SH-SY5Y | ENSG00000154556 | sorbin and SH3 domain containing 2 |
| **SORCS1** | SH-SY5Y | ENSG00000108018 | sortilin-related VPS10 domain containing receptor 1 |
| **SOX6** | ACHN | ENSG00000110693 | SRY (sex determining region Y)-box 6 |
| **SP140** | HepG2 | ENSG00000079263 | SP140 nuclear body protein |
| **SP5** | HepG2 | ENSG00000204335 | Sp5 transcription factor |
| **SPC24** | SH-SY5Y | ENSG00000161888 | SPC24, NDC80 kinetochore complex component, homolog (*S. cerevisiae*) |
| **SPC25** | SH-SY5Y | ENSG00000152253 | SPC25, NDC80 kinetochore complex component, homolog (*S. cerevisiae*) |
| **SPDEF** | HepG2 | ENSG00000124664 | SAM pointed domain containing ets transcription factor |
| **SPOCK2** | HepG2 | ENSG00000107742 | sparc/osteonectin, cwcv and kazal-like domains proteoglycan (testican) 2 |
| **SPRY2** | IMR-32 | ENSG00000136158 | sprouty homolog 2 (Drosophila) |
| **SPTY2D1** | SH-SY5Y | ENSG00000179119 | SPT2, Suppressor of Ty, domain containing 1 (*S. cerevisiae*) |
| **SQSTM1** | SH-SY5Y | ENSG00000161011 | sequestosome 1 |
| **SRA1** | ACHN | ENSG00000213523 | steroid receptor RNA activator 1 |
| **SREBF1** | HeLa-S3 | ENSG00000072310 | sterol regulatory element binding transcription factor 1 |
| **SRGAP1** | U-87 MG | ENSG00000196935 | SLIT-ROBO Rho GTPase activating protein 1 |
| **SRRT** | ACHN | ENSG00000087087 | serrate RNA effector molecule homolog (Arabidopsis) |
| **SRSF4** | ACHN | ENSG00000116350 | serine/arginine-rich splicing factor 4 |
| **SSFA2** | SH-SY5Y | ENSG00000138434 | sperm specific antigen 2 |
| **SSH2** | SH-SY5Y | ENSG00000141298 | slingshot protein phosphatase 2 |
| **SSTR2** | SH-SY5Y | ENSG00000180616 | somatostatin receptor 2 |
| **ST6GAL1** | HepG2 | ENSG00000073849 | ST6 beta-galactosamide alpha-2,6-sialyltranferase 1 |
| **ST6GAL2** | hFOB1.19 | ENSG00000144057 | ST6 beta-galactosamide alpha-2,6-sialyltranferase 2 |
| **ST8SIA2** | SH-SY5Y | ENSG00000140557 | ST8 alpha-N-acetyl-neuraminide alpha-2,8-sialyltransferase 2 |
| **STAC** | SH-SY5Y | ENSG00000144681 | SH3 and cysteine rich domain |
| **STARD4** | SH-SY5Y | ENSG00000164211 | StAR-related lipid transfer (START) domain containing 4 |
| **STARD9** | SH-SY5Y | ENSG00000159433 | StAR-related lipid transfer (START) domain containing 9 |
| **STAT5A** | SH-SY5Y | ENSG00000126561 | signal transducer and activator of transcription 5A |
| **STC1** | U-87 MG | ENSG00000159167 | stanniocalcin 1 |
| **STIL** | SH-SY5Y | ENSG00000123473 | SCL/TAL1 interrupting locus |
| **STMN1** | SH-SY5Y | ENSG00000117632 | stathmin 1 |
| **STMN4** | SH-SY5Y | ENSG00000015592 | stathmin-like 4 |
| **STOML1** | ACHN | ENSG00000067221 | stomatin (EPB72)-like 1 |
| **STX11** | ACHN | ENSG00000135604 | syntaxin 11 |
| **STX3** | SH-SY5Y | ENSG00000166900 | syntaxin 3 |
| **STYK1** | U-87 MG | ENSG00000060140 | serine/threonine/tyrosine kinase 1 |
| **SULT1C4** | SH-SY5Y | ENSG00000198075 | sulfotransferase family, cytosolic, 1C, member 4 |
| **SULT2A1** | HepG2 | ENSG00000105398 | sulfotransferase family, cytosolic, 2A, dehydroepiandrosterone (DHEA)-preferring, member 1 |
| **SUSD2** | HepG2 | ENSG00000099994 | sushi domain containing 2 |
| **SV2C** | SH-SY5Y | ENSG00000122012 | synaptic vesicle glycoprotein 2C |
| **SYNPO** | HepG2 | ENSG00000171992 | synaptopodin |
| **SYNPO2L** | HepG2 | ENSG00000166317 | synaptopodin 2-like |
| **SYT5** | SH-SY5Y | ENSG00000129990 | synaptotagmin V |
| **SYTL2** | SH-SY5Y | ENSG00000137501 | synaptotagmin-like 2 |
| **SYTL4** | IMR-32 | ENSG00000102362 | synaptotagmin-like 4 |
| **TACC1** | SH-SY5Y | ENSG00000147526 | transforming, acidic coiled-coil containing protein 1 |
| **TANC2** | HepG2 | ENSG00000170921 | tetratricopeptide repeat, ankyrin repeat and coiled-coil containing 2 |
| **TANK** | ACHN | ENSG00000136560 | TRAF family member-associated NFKB activator |
| **TAPBPL** | ACHN | ENSG00000139192 | TAP binding protein-like |
| **TARS** | SH-SY5Y | ENSG00000113407 | threonyl-tRNA synthetase |
| **TAX1BP1** | SH-SY5Y | ENSG00000106052 | Tax1 (human T-cell leukemia virus type I) binding protein 1 |
| **TBC1D16** | SH-SY5Y | ENSG00000167291 | TBC1 domain family, member 16 |
| **TBC1D22B** | ACHN | ENSG00000065491 | TBC1 domain family, member 22B |
| **TBC1D2B** | HepG2 | ENSG00000167202 | TBC1 domain family, member 2B |
| **TBCC** | SH-SY5Y | ENSG00000124659 | tubulin folding cofactor C |
| **TBX3** | U-87 MG | ENSG00000135111 | T-box 3 |
| **TCERG1L** | SH-SY5Y | ENSG00000176769 | transcription elongation regulator 1-like |
| **TCF19** | SH-SY5Y | ENSG00000137310 | transcription factor 19 |
| **TEF** | ACHN | ENSG00000167074 | thyrotrophic embryonic factor |
| **TENM3** | SH-SY5Y | ENSG00000218336 | teneurin transmembrane protein 3 |
| **TEP1** | ACHN | ENSG00000129566 | telomerase-associated protein 1 |
| **TERF2** | SH-SY5Y | ENSG00000132604 | telomeric repeat binding factor 2 |
| **TERF2IP** | HepG2 | ENSG00000166848 | telomeric repeat binding factor 2, interacting protein |
| **TEX14** | ACHN | ENSG00000121101 | testis expressed 14 |
| **TEX15** | SH-SY5Y | ENSG00000133863 | testis expressed 15 |
| **TF** | HepG2 | ENSG00000091513 | transferrin |
| **TFB2M** | SH-SY5Y | ENSG00000162851 | transcription factor B2, mitochondrial |
| **TFDP1** | SH-SY5Y | ENSG00000198176 | transcription factor Dp-1 |
| **TFEB** | SH-SY5Y | ENSG00000112561 | transcription factor EB |
| **TGFA** | U-87 MG | ENSG00000163235 | transforming growth factor, alpha |
| **TGFBR1** | U-87 MG | ENSG00000106799 | transforming growth factor, beta receptor 1 |
| **TGIF1** | U-87 MG | ENSG00000177426 | TGFB-induced factor homeobox 1 |
| **TGIF2** | ACHN | ENSG00000118707 | TGFB-induced factor homeobox 2 |
| **TGM2** | HepG2 | ENSG00000198959 | transglutaminase 2 (C polypeptide, protein-glutamine-gamma-glutamyltransferase) |
| **TGM4** | HepG2 | ENSG00000163810 | transglutaminase 4 (prostate) |
| **TGM5** | HepG2 | ENSG00000104055 | transglutaminase 5 |
| **THAP1** | ACHN | ENSG00000131931 | THAP domain containing, apoptosis associated protein 1 |
| **THBD** | ACHN | ENSG00000178726 | thrombomodulin |
| **THSD4** | SH-SY5Y | ENSG00000187720 | thrombospondin, type I, domain containing 4 |
| **THSD7A** | SH-SY5Y | ENSG00000005108 | thrombospondin, type I, domain containing 7A |
| **THSD7B** | IMR-32 | ENSG00000144229 | thrombospondin, type I, domain containing 7B |
| **THUMPD2** | ACHN | ENSG00000138050 | THUMP domain containing 2 |
| **TIAM1** | SH-SY5Y | ENSG00000156299 | T-cell lymphoma invasion and metastasis 1 |
| **TIMP2** | HepG2 | ENSG00000035862 | TIMP metallopeptidase inhibitor 2 |
| **TLE3** | U-87 MG | ENSG00000140332 | transducin-like enhancer of split 3 (E(sp1) homolog, Drosophila) |
| **TLR4** | HepG2 | ENSG00000136869 | toll-like receptor 4 |
| **TMC7** | HepG2 | ENSG00000170537 | transmembrane channel-like 7 |
| **TMC8** | HepG2 | ENSG00000167895 | transmembrane channel-like 8 |
| **TMEM108** | SH-SY5Y | ENSG00000144868 | transmembrane protein 108 |
| **TMEM132B** | Saos-2 | ENSG00000139364 | transmembrane protein 132B |
| **TMEM158** | SH-SY5Y | ENSG00000249992 | transmembrane protein 158 (gene/pseudogene) |
| **TMEM201** | HepG2 | ENSG00000188807 | transmembrane protein 201 |
| **TMEM215** | SH-SY5Y | ENSG00000188133 | transmembrane protein 215 |
| **TMEM45A** | U-2 Os | ENSG00000181458 | transmembrane protein 45A |
| **TMEM86B** | HepG2 | ENSG00000180089 | transmembrane protein 86B |
| **TMEM92** | HepG2 | ENSG00000167105 | transmembrane protein 92 |
| **TMEM97** | SH-SY5Y | ENSG00000109084 | transmembrane protein 97 |
| **TMOD2** | SH-SY5Y | ENSG00000128872 | tropomodulin 2 (neuronal) |
| **TMPO** | SH-SY5Y | ENSG00000120802 | thymopoietin |
| **TMTC2** | SH-SY5Y | ENSG00000179104 | transmembrane and tetratricopeptide repeat containing 2 |
| **TMX3** | ACHN | ENSG00000166479 | thioredoxin-related transmembrane protein 3 |
| **TMX4** | SH-SY5Y | ENSG00000125827 | thioredoxin-related transmembrane protein 4 |
| **TNC** | SH-SY5Y | ENSG00000041982 | tenascin C |
| **TNFRSF12A** | SH-SY5Y | ENSG00000006327 | tumor necrosis factor receptor superfamily, member 12A |
| **TNFRSF1B** | HepG2 | ENSG00000028137 | tumor necrosis factor receptor superfamily, member 1B |
| **TNFRSF9** | ACHN | ENSG00000049249 | tumor necrosis factor receptor superfamily, member 9 |
| **TNFSF10** | hFOB1.19 | ENSG00000121858 | tumor necrosis factor (ligand) superfamily, member 10 |
| **TNIP2** | ACHN | ENSG00000168884 | TNFAIP3 interacting protein 2 |
| **TNKS1BP1** | ACHN | ENSG00000149115 | tankyrase 1 binding protein 1, 182kDa |
| **TOB1** | SH-SY5Y | ENSG00000141232 | transducer of ERBB2, 1 |
| **TONSL** | HepG2 | ENSG00000160949 | tonsoku-like, DNA repair protein |
| **TOP2A** | SH-SY5Y | ENSG00000131747 | topoisomerase (DNA) II alpha 170kDa |
| **TP53I3** | IMR-32 | ENSG00000115129 | tumor protein p53 inducible protein 3 |
| **TP73** | SH-SY5Y | ENSG00000078900 | tumor protein p73 |
| **TPSAB1** | SH-SY5Y | ENSG00000172236 | tryptase alpha/beta 1 |
| **TPSB2** | SH-SY5Y | ENSG00000197253 | tryptase beta 2 (gene/pseudogene) |
| **TPSD1** | SH-SY5Y | ENSG00000095917 | tryptase delta 1 |
| **TRA2A** | SH-SY5Y | ENSG00000164548 | transformer 2 alpha homolog (Drosophila) |
| **TRA2B** | ACHN | ENSG00000136527 | transformer 2 beta homolog (Drosophila) |
| **TRAF2** | ACHN | ENSG00000127191 | TNF receptor-associated factor 2 |
| **TRAF3** | ACHN | ENSG00000131323 | TNF receptor-associated factor 3 |
| **TRAF4** | ACHN | ENSG00000076604 | TNF receptor-associated factor 4 |
| **TRAFD1** | ACHN | ENSG00000135148 | TRAF-type zinc finger domain containing 1 |
| **TRAPPC9** | SH-SY5Y | ENSG00000167632 | trafficking protein particle complex 9 |
| **TRIM26** | ACHN | ENSG00000234127 | tripartite motif containing 26 |
| **TRIM38** | ACHN | ENSG00000112343 | tripartite motif containing 38 |
| **TRIM52** | U-87 MG | ENSG00000183718 | tripartite motif containing 52 |
| **TRIM8** | SH-SY5Y | ENSG00000171206 | tripartite motif containing 8 |
| **TROAP** | SH-SY5Y | ENSG00000135451 | trophinin associated protein |
| **TRPC4** | ACHN | ENSG00000133107 | transient receptor potential cation channel, subfamily C, member 4 |
| **TSC22D3** | HepG2 | ENSG00000157514 | TSC22 domain family, member 3 |
| **TSPAN1** | HepG2 | ENSG00000117472 | tetraspanin 1 |
| **TSPAN8** | SH-SY5Y | ENSG00000127324 | tetraspanin 8 |
| **TSPEAR** | SH-SY5Y | ENSG00000175894 | thrombospondin-type laminin G domain and EAR repeats |
| **TTC28** | SH-SY5Y | ENSG00000100154 | tetratricopeptide repeat domain 28 |
| **TTC32** | U-87 MG | ENSG00000183891 | tetratricopeptide repeat domain 32 |
| **TTC40** | ACHN | ENSG00000171811 | tetratricopeptide repeat domain 40 |
| **TTR** | HepG2 | ENSG00000118271 | transthyretin |
| **TTYH1** | HepG2 | ENSG00000167614 | tweety homolog 1 (Drosophila) |
| **TUBA1A** | SH-SY5Y | ENSG00000167552 | tubulin, alpha 1a |
| **TUBA1B** | SH-SY5Y | ENSG00000123416 | tubulin, alpha 1b |
| **TUBB** | SH-SY5Y | ENSG00000196230 | tubulin, beta class I |
| **TUBB3** | U-87 MG | ENSG00000198211 | tubulin, beta 3 class III |
| **TUBB6** | U-87 MG | ENSG00000176014 | tubulin, beta 6 class V |
| **TUBBP1** | SH-SY5Y | ENSG00000127589 | tubulin, beta pseudogene 1 |
| **TUBG1** | SH-SY5Y | ENSG00000131462 | tubulin, gamma 1 |
| **TUBGCP6** | ACHN | ENSG00000128159 | tubulin, gamma complex associated protein 6 |
| **TUFT1** | ACHN | ENSG00000143367 | tuftelin 1 |
| **TXNRD2** | ACHN | ENSG00000184470 | thioredoxin reductase 2 |
| **TYK2** | ACHN | ENSG00000105397 | tyrosine kinase 2 |
| **TYMS** | SH-SY5Y | ENSG00000176890 | thymidylate synthetase |
| **UAP1** | ACHN | ENSG00000117143 | UDP-N-acteylglucosamine pyrophosphorylase 1 |
| **UBE2C** | SH-SY5Y | ENSG00000175063 | ubiquitin-conjugating enzyme E2C |
| **UBE2L6** | ACHN | ENSG00000156587 | ubiquitin-conjugating enzyme E2L 6 |
| **UBE2T** | SH-SY5Y | ENSG00000077152 | ubiquitin-conjugating enzyme E2T (putative) |
| **UBE2W** | SH-SY5Y | ENSG00000104343 | ubiquitin-conjugating enzyme E2W (putative) |
| **UBN2** | U-87 MG | ENSG00000157741 | ubinuclein 2 |
| **UBR5** | U-87 MG | ENSG00000104517 | ubiquitin protein ligase E3 component n-recognin 5 |
| **UBXN11** | ACHN | ENSG00000158062 | UBX domain protein 11 |
| **UGCG** | U-87 MG | ENSG00000148154 | UDP-glucose ceramide glucosyltransferase |
| **UGT2B10** | HepG2 | ENSG00000109181 | UDP glucuronosyltransferase 2 family, polypeptide B10 |
| **UHRF1** | SH-SY5Y | ENSG00000034063 | ubiquitin-like with PHD and ring finger domains 1 |
| **UNC5C** | SH-SY5Y | ENSG00000182168 | unc-5 homolog C (*C. elegans*) |
| **UNK** | ACHN | ENSG00000132478 | unkempt homolog (Drosophila) |
| **UPP1** | SH-SY5Y | ENSG00000183696 | uridine phosphorylase 1 |
| **UQCRFS1P1** | SH-SY5Y | ENSG00000226085 | ubiquinol-cytochrome c reductase, Rieske iron-sulfur polypeptide 1 pseudogene 1 |
| **USP11** | ACHN | ENSG00000102226 | ubiquitin specific peptidase 11 |
| **USP54** | HepG2 | ENSG00000166348 | ubiquitin specific peptidase 54 |
| **UTP14A** | SH-SY5Y | ENSG00000156697 | UTP14, U3 small nucleolar ribonucleoprotein, homolog A (yeast) |
| **UVRAG** | ACHN | ENSG00000198382 | UV radiation resistance associated |
| **UVSSA** | ACHN | ENSG00000163945 | UV-stimulated scaffold protein A |
| **VAMP2** | ACHN | ENSG00000220205 | vesicle-associated membrane protein 2 (synaptobrevin 2) |
| **VASH2** | SH-SY5Y | ENSG00000143494 | vasohibin 2 |
| **VASN** | ACHN | ENSG00000168140 | vasorin |
| **VAV3** | HepG2 | ENSG00000134215 | vav 3 guanine nucleotide exchange factor |
| **VCAN** | SH-SY5Y | ENSG00000038427 | versican |
| **VCPIP1** | U-87 MG | ENSG00000175073 | valosin containing protein (p97)/p47 complex interacting protein 1 |
| **VGF** | IMR-32 | ENSG00000128564 | VGF nerve growth factor inducible |
| **VIM-AS1** | U-87 MG | ENSG00000229124 | VIM antisense RNA 1 |
| **VIPR2** | SH-SY5Y | ENSG00000106018 | vasoactive intestinal peptide receptor 2 |
| **VPS13B** | SH-SY5Y | ENSG00000132549 | vacuolar protein sorting 13 homolog B (yeast) |
| **VPS13C** | SH-SY5Y | ENSG00000129003 | vacuolar protein sorting 13 homolog C (*S. cerevisiae*) |
| **VSIG10** | ACHN | ENSG00000176834 | V-set and immunoglobulin domain containing 10 |
| **WDR24** | ACHN | ENSG00000127580 | WD repeat domain 24 |
| **WDR34** | SH-SY5Y | ENSG00000119333 | WD repeat domain 34 |
| **WDR62** | ACHN | ENSG00000075702 | WD repeat domain 62 |
| **WDR74** | SH-SY5Y | ENSG00000133316 | WD repeat domain 74 |
| **WDR76** | SH-SY5Y | ENSG00000092470 | WD repeat domain 76 |
| **WDR90** | ACHN | ENSG00000161996 | WD repeat domain 90 |
| **WFDC3** | HepG2 | ENSG00000124116 | WAP four-disulfide core domain 3 |
| **WFS1** | HepG2 | ENSG00000109501 | Wolfram syndrome 1 (wolframin) |
| **WHSC1** | SH-SY5Y | ENSG00000109685 | Wolf-Hirschhorn syndrome candidate 1 |
| **WIPI1** | ACHN | ENSG00000070540 | WD repeat domain, phosphoinositide interacting 1 |
| **WNT10B** | HepG2 | ENSG00000169884 | wingless-type MMTV integration site family, member 10B |
| **WNT11** | HepG2 | ENSG00000085741 | wingless-type MMTV integration site family, member 11 |
| **WNT5B** | SK-N-SH | ENSG00000111186 | wingless-type MMTV integration site family, member 5B |
| **WNT9A** | HepG2 | ENSG00000143816 | wingless-type MMTV integration site family, member 9A |
| **WRAP53** | ACHN | ENSG00000141499 | WD repeat containing, antisense to TP53 |
| **WWOX** | SH-SY5Y | ENSG00000186153 | WW domain containing oxidoreductase |
| **XIRP1** | U-87 MG | ENSG00000168334 | xin actin-binding repeat containing 1 |
| **XIST** | SH-SY5Y | ENSG00000229807 | X inactive specific transcript (non-protein coding) |
| **XKR7** | ACHN | ENSG00000101321 | XK, Kell blood group complex subunit-related family, member 7 |
| **XPC** | ACHN | ENSG00000154767 | xeroderma pigmentosum, complementation group C |
| **XPR1** | SH-SY5Y | ENSG00000143324 | xenotropic and polytropic retrovirus receptor 1 |
| **XRCC1** | ACHN | ENSG00000073050 | X-ray repair complementing defective repair in Chinese hamster cells 1 |
| **XRCC2** | SH-SY5Y | ENSG00000196584 | X-ray repair complementing defective repair in Chinese hamster cells 2 |
| **YARS** | SH-SY5Y | ENSG00000134684 | tyrosyl-tRNA synthetase |
| **YTHDC1** | U-87 MG | ENSG00000083896 | YTH domain containing 1 |
| **YY1AP1** | ACHN | ENSG00000163374 | YY1 associated protein 1 |
| **ZBTB1** | SH-SY5Y | ENSG00000126804 | zinc finger and BTB domain containing 1 |
| **ZBTB11** | SH-SY5Y | ENSG00000066422 | zinc finger and BTB domain containing 11 |
| **ZBTB17** | ACHN | ENSG00000116809 | zinc finger and BTB domain containing 17 |
| **ZBTB21** | U-87 MG | ENSG00000173276 | zinc finger and BTB domain containing 21 |
| **ZBTB38** | ACHN | ENSG00000177311 | zinc finger and BTB domain containing 38 |
| **ZBTB40** | U-87 MG | ENSG00000184677 | zinc finger and BTB domain containing 40 |
| **ZBTB43** | U-87 MG | ENSG00000169155 | zinc finger and BTB domain containing 43 |
| **ZC3HAV1** | ACHN | ENSG00000105939 | zinc finger CCCH-type, antiviral 1 |
| **ZCCHC2** | HepG2 | ENSG00000141664 | zinc finger, CCHC domain containing 2 |
| **ZCCHC6** | ACHN | ENSG00000083223 | zinc finger, CCHC domain containing 6 |
| **ZEB2** | U-87 MG | ENSG00000169554 | zinc finger E-box binding homeobox 2 |
| **ZFAND2A** | SH-SY5Y | ENSG00000178381 | zinc finger, AN1-type domain 2A |
| **ZFYVE27** | ACHN | ENSG00000155256 | zinc finger, FYVE domain containing 27 |
| **ZG16B** | HepG2 | ENSG00000162078 | zymogen granule protein 16B |
| **ZKSCAN1** | U-87 MG | ENSG00000106261 | zinc finger with KRAB and SCAN domains 1 |
| **ZKSCAN8** | U-87 MG | ENSG00000198315 | zinc finger with KRAB and SCAN domains 8 |
| **ZNF132** | ACHN | ENSG00000131849 | zinc finger protein 132 |
| **ZNF146** | ACHN | ENSG00000167635 | zinc finger protein 146 |
| **ZNF165** | ACHN | ENSG00000197279 | zinc finger protein 165 |
| **ZNF202** | ACHN | ENSG00000166261 | zinc finger protein 202 |
| **ZNF211** | ACHN | ENSG00000121417 | zinc finger protein 211 |
| **ZNF222** | SH-SY5Y | ENSG00000159885 | zinc finger protein 222 |
| **ZNF267** | SH-SY5Y | ENSG00000185947 | zinc finger protein 267 |
| **ZNF3** | ACHN | ENSG00000166526 | zinc finger protein 3 |
| **ZNF316** | SH-SY5Y | ENSG00000205903 | zinc finger protein 316 |
| **ZNF317** | SH-SY5Y | ENSG00000130803 | zinc finger protein 317 |
| **ZNF335** | SH-SY5Y | ENSG00000198026 | zinc finger protein 335 |
| **ZNF34** | SK-N-SH | ENSG00000196378 | zinc finger protein 34 |
| **ZNF367** | SH-SY5Y | ENSG00000165244 | zinc finger protein 367 |
| **ZNF394** | SH-SY5Y | ENSG00000160908 | zinc finger protein 394 |
| **ZNF408** | SH-SY5Y | ENSG00000175213 | zinc finger protein 408 |
| **ZNF423** | Saos-2 | ENSG00000102935 | zinc finger protein 423 |
| **ZNF461** | SH-SY5Y | ENSG00000197808 | zinc finger protein 461 |
| **ZNF483** | SH-SY5Y | ENSG00000173258 | zinc finger protein 483 |
| **ZNF503** | U-87 MG | ENSG00000165655 | zinc finger protein 503 |
| **ZNF517** | HepG2 | ENSG00000197363 | zinc finger protein 517 |
| **ZNF555** | ACHN | ENSG00000186300 | zinc finger protein 555 |
| **ZNF608** | ACHN | ENSG00000168916 | zinc finger protein 608 |
| **ZNF622** | SH-SY5Y | ENSG00000173545 | zinc finger protein 622 |
| **ZNF646** | ACHN | ENSG00000167395 | zinc finger protein 646 |
| **ZNF667** | SH-SY5Y | ENSG00000198046 | zinc finger protein 667 |
| **ZNF687** | ACHN | ENSG00000143373 | zinc finger protein 687 |
| **ZNF76** | ACHN | ENSG00000065029 | zinc finger protein 76 |
| **ZNF764** | ACHN | ENSG00000169951 | zinc finger protein 764 |
| **ZNF770** | U-87 MG | ENSG00000198146 | zinc finger protein 770 |
| **ZNF776** | SH-SY5Y | ENSG00000152443 | zinc finger protein 776 |
| **ZNF79** | ACHN | ENSG00000196152 | zinc finger protein 79 |
| **ZNF821** | ACHN | ENSG00000102984 | zinc finger protein 821 |
| **ZNF860** | HepG2 | ENSG00000197385 | zinc finger protein 860 |
| **ZRSR2** | SH-SY5Y | ENSG00000169249 | zinc finger (CCCH type), RNA-binding motif and serine/arginine rich 2 |
| **ZSWIM4** | ACHN | ENSG00000132003 | zinc finger, SWIM-type containing 4 |
| **ZWINT** | SH-SY5Y | ENSG00000122952 | ZW10 interactor, kinetochore protein |
| **NA** | U-2 Os | ENSG00000186031 | NA |
| **NA** | HepG2 | ENSG00000203347 | NA |
| **NA** | HepG2 | ENSG00000245411 | NA |
| **NA** | SH-SY5Y | ENSG00000246181 | NA |
| **NA** | SH-SY5Y | ENSG00000246493 | NA |
| **NA** | HepG2 | ENSG00000256017 | NA |
| **NA** | SK-N-SH | ENSG00000257054 | NA |
| **NA** | ACHN | ENSG00000257066 | NA |
