# Supplementary material for: Identification of Putative Target Genes of the Transcription Factor RUNX2
Source: PLoS One. 2013 Dec 12;8(12):e83218. doi: 10.1371/journal.pone.0083218 (PMC3861491; doi:10.1371/journal.pone.0083218)
Supplement: Table S3 — Average directions of expression differences between RUNX2 transfected and mock transfected cells. The values 1 and 0 indicate higher and lower expression of a gene after RUNX2 transfection, respectively. Average numbers across all genes and differentially expressed genes that overlap between cell lines. (DOCX) [file pone.0083218.s006.docx]

**Table S3**. Average directions of expression differences between *RUNX2* transfected and mock transfected cells. The values 1 and 0 indicate higher and lower expression of a gene after *RUNX2* transfection, respectively. Average numbers across all genes and differentially expressed genes that overlap between cell lines.

| **Cell line** | **All genes** | **1 cell line** | **2 cell lines** | **3 cell lines** | **4 cell lines** | **5 cell lines** |
| --- | --- | --- | --- | --- | --- | --- |
| **Saos-2** | 0.50 | 0.55 | 0.63 | 0.60 | 0.63 | 0.63 |
| **U-2 Os** | 0.50 | 0.58 | 0.59 | 0.63 | 0.67 | 0.63 |
| **ACHN** | 0.52 | 0.43 | 0.43 | 0.48 | 0.52 | 0.38 |
| **U-87 MG** | 0.50 | 0.55 | 0.64 | 0.59 | 0.58 | 0.63 |
| **HeLa-S3** | 0.50 | 0.55 | 0.69 | 0.72 | 0.91 | 1.00 |
| **SH-SY5Y** | 0.52 | 0.47 | 0.60 | 0.69 | 0.79 | 0.75 |
| **IMR-32** | 0.49 | 0.45 | 0.35 | 0.19 | 0.09 | 0.00 |
| **HepG2** | 0.50 | 0.58 | 0.72 | 0.84 | 0.91 | 1.00 |
| **SK-N-SH** | 0.49 | 0.54 | 0.61 | 0.66 | 0.71 | 0.63 |
| **hFOB1.19** | 0.51 | 0.51 | 0.60 | 0.81 | 0.79 | 0.75 |
| **All lines** | 0.50 | 0.52 | 0.59 | 0.64 | 0.67 | 0.65 |
