# Supplementary material for: Identification of Putative Target Genes of the Transcription Factor RUNX2
Source: PLoS One. 2013 Dec 12;8(12):e83218. doi: 10.1371/journal.pone.0083218 (PMC3861491; doi:10.1371/journal.pone.0083218)
Supplement: Table S4 — List of 27 genes to which RUNX2 was found to bind by chromatin immunoprecipitation and genes found to be differentially expressed in the present study in two or more cell lines after RUNX2 overexpression. (DOCX) [file pone.0083218.s007.docx]

**Table S4.** List of 27 genes to which RUNX2 was found to bind by chromatin immunoprecipitation and genes found to be differentially expressed in the present study in two or more cell lines after *RUNX2* overexpression.

| **Gene name** | **Description** |
| --- | --- |
| ***ABL2*** | v-abl Abelson murine leukemia viral oncogene homolog 2 |
| ***ADAMTS5*** | ADAM metallopeptidase with thrombospondin type 1 motif, 5 |
| ***ANKRD28*** | ankyrin repeat domain 28 |
| ***CCNL1*** | cyclin L1 |
| ***CCRN4L*** | CCR4 carbon catabolite repression 4-like (*S. cerevisiae*) |
| ***CDC6*** | cell division cycle 6 |
| ***CTH*** | cystathionase (cystathionine gamma-lyase) |
| ***DPYSL3*** | dihydropyrimidinase-like 3 |
| ***FAT1*** | FAT tumor suppressor homolog 1 (Drosophila) |
| ***GADD45A*** | growth arrest and DNA-damage-inducible, alpha |
| ***GLRX*** | glutaredoxin (thioltransferase) |
| ***KLF5*** | Kruppel-like factor 5 (intestinal) |
| ***KLF9*** | Kruppel-like factor 9 |
| ***LACC1*** | laccase (multicopper oxidoreductase) domain containing 1 |
| ***MCM2*** | minichromosome maintenance complex component 2 |
| ***MCM3*** | minichromosome maintenance complex component 3 |
| ***MLL2*** | myeloid/lymphoid or mixed-lineage leukemia 2 |
| ***MMP14*** | matrix metallopeptidase 14 (membrane-inserted) |
| ***NCAPD2*** | non-SMC condensin I complex, subunit D2 |
| ***PPM1D*** | protein phosphatase, Mg2+/Mn2+ dependent, 1D |
| ***RSRC2*** | arginine/serine-rich coiled-coil 2 |
| ***SERPINA6*** | serpin peptidase inhibitor, clade A (alpha-1 antiproteinase, antitrypsin), member 6 |
| ***SLC3A2*** | solute carrier family 3 (activators of dibasic and neutral amino acid transport), member 2 |
| ***SNAPC1*** | small nuclear RNA activating complex, polypeptide 1, 43kDa |
| ***SVIL*** | supervillin |
| ***TBC1D5*** | TBC1 domain family, member 5 |
| ***TSC22D1*** | TSC22 domain family, member 1 |
