# Supplementary material for: Identification of Putative Target Genes of the Transcription Factor RUNX2
Source: PLoS One. 2013 Dec 12;8(12):e83218. doi: 10.1371/journal.pone.0083218 (PMC3861491; doi:10.1371/journal.pone.0083218)
Supplement: Table S5 — Categories in the “Biological Process” gene ontology, which are enriched among genes differentially expressed in two or more cell lines after overexpression of RUNX2, using a hypergeometric test implemented in FUNC. (DOCX) [file pone.0083218.s008.docx]

**Table S5.** Categories in the “Biological Process” gene ontology, which are enriched among genes differentially expressed in two or more cell lines after overexpression of *RUNX2*, using a hypergeometric test implemented in *FUNC*.

| **GO term** | **p-value** | **Genes in category** |
| --- | --- | --- |
| electron transport chain | 0.00000000001 | *GLRX; MT-ATP6; MT-ATP8; MT-CO1; MT-CO2; MT-CO3; MT-CYB; MT-ND1; MT-ND2; MT-ND4; MT-ND4L; MT-ND5; MT-ND6; TXNRD1* |
| cell cycle | 0.00000000003 | *ARHGEF2; CCNA2; CCNL1; CDC6; CDCA5; CDKN2C; CDT1; CTGF; DDIT3; DST; DYNC1H1; E2F1; ESPL1; FANCI; FOSL1; FOXM1; GADD45A; GADD45B; GTSE1; ID3; IGF2; IL1A; IL8; INCENP; IRF1; JMY; JUNB; KIAA0101; KIF18B; KIF20A; LIG1; MACF1; MCM2; MCM3; MCM5; MKI67; MYBL2; NCAPD2; NCAPH; NGFR; PKMYT1; PLK1; PLK2; PLK3; POLE; PPM1D; PPP1R15A; PRKAB1; RRM2; SESN2; SPAG5; TACC3; TGFB2; THBS1; TICRR; TIMELESS; TNFAIP3; TSPYL2; WNT5A* |
| cell-substrate junction assembly | 0.00000000006 | *DST; LAMA5; LAMB3; MACF1; MMP14; PTPRJ; THBS1; VEGFA* |
| regulation of protein phosphorylation | 0.0000000002 | *ATF3; ATF4; BDKRB2; CCNA2; CCNL1; CD44; CDC6; CDKN2C; CTGF; DDIT3; DKK1; DUSP10; DUSP4; DUSP5; ENPP1; ERBB3; GADD45A; GADD45B; IGF2; IGFBP3; IL11; IL23A; IL6; IL8; NRG1; PDGFA; PKMYT1; PLK1; PTPRJ; RELN; SPRY4; TGFB2; THBS1; TNFAIP3; TRIB3; TSPYL2; VEGFA; WNT5A; ZFYVE28* |
| apoptotic process | 0.0000000002 | *AEN; AMIGO2; ANGPTL4; ARHGEF2; ATF4; AXL; BDKRB2; BIRC3; CD44; CDKN2C; CEBPB; CSRNP1; CTGF; CTH; DDIT3; DSP; E2F1; EGR1; ERBB3; ESPL1; F3; FOSL1; GADD45A; GADD45B; HMOX1; ID3; IGFBP3; IL1A; IL6; IRF1; JMY; KALRN; KLF10; LMNB1; MMP9; NGFR; NQO1; NRG1; NUAK2; PHLDA1; PHLDA3; PLK1; PLK2; PLK3; PMAIP1; PPP1R15A; SNAI2; STK17B; TGFB2; THBS1; TNFAIP3; TNFRSF10D; TNS4; TRAF1; TRIB3; TSC22D1; UNC5B; VDR; VEGFA; WNT5A* |
| cellular component movement | 0.0000000002 | *ABL2; ARC; AXL; COL1A1; CTGF; DNAH17; DPYSL3; DSP; DST; DYNC1H1; EFNB2; F3; FAT1; FMNL1; FSCN1; HBEGF; HMOX1; ICAM1; IGFBP3; IL23A; IL6; IL8; ITGAM; JMY; KIF18B; KIF20A; KIF26B; LAMA5; LRP1; MACF1; MMP14; MMP9; NAV1; NRG1; PDGFA; PLAU; PLAUR; PLXNA4; PTPRJ; RELN; ROBO4; SHROOM2; SLC3A2; SNAI2; SPOCK1; SYNE2; TGFB2; THBS1; VEGFA; WNT5A* |
| blood vessel development | 0.0000000002 | *ANGPTL4; COL1A1; CTGF; DDIT3; EFNB2; EGR1; F3; FOSL1; FOXM1; HMOX1; IL1A; IL6; IL8; JUNB; KLF5; LAMA5; LRP1; MMP14; PDGFA; PTPRJ; ROBO4; STRA6; TGFB2; THBS1; TIPARP; TNFAIP3; VEGFA; WNT5A* |
| regulation of leukocyte migration | 0.0000000006 | *HMOX1; ICAM1; IL23A; IL6; IL8; PTPRJ; THBS1; VEGFA; WNT5A* |
| response to stress | 0.0000000009 | *AEN; ANGPTL4; ARHGEF2; ATF3; ATF4; AXL; BDKRB2; BIRC3; CCNA2; CD44; CDCA5; CEBPB; COL1A1; CTGF; CTH; DCBLD2; DDIT3; DMBT1; DOCK9; DPYSL3; DST; DUSP10; DUSP4; E2F1; EGR1; ERBB3; F3; FANCI; FOSL1; FOXM1; GABARAPL1; GADD45A; GADD45B; GTSE1; HBEGF; HMOX1; ICAM1; ID3; IGF2; IL11; IL1A; IL1R1; IL23A; IL6; IL8; IRF1; ITGA2; ITGAM; JMY; KIAA0101; LIG1; MACF1; MAFF; MKI67; MMP14; MT2A; NFKBIB; NQO1; NR1D1; NRG1; NT5E; NUAK2; PDGFA; PHLDA3; PLA2G4C; PLAU; PLAUR; PLK1; PLK3; PMAIP1; POLE; PPP1R15A; PTPRJ; RELN; SEMA7A; SLC3A2; SNAI2; STC2; TACC3; TGFB2; THBS1; TICRR; TIMELESS; TNFAIP3; TRIB3; TRPV2; TSPYL2; TXNRD1; VEGFA; WNT5A* |
| protein phosphorylation | 0.000000003 | *ABL2; ALPK2; ATF3; ATF4; AXL; BDKRB2; CCNA2; CCNL1; CD44; CDC6; CDKN2C; CTGF; DDIT3; DKK1; DUSP10; DUSP4; DUSP5; ENPP1; ERBB3; GADD45A; GADD45B; IGF2; IGFBP3; IL11; IL23A; IL6; IL8; IRF1; KALRN; NRG1; NUAK2; PDGFA; PKMYT1; PLK1; PLK2; PLK3; PRKAB1; PTPRJ; RELN; RIOK3; SPRY4; STK17B; TGFB2; THBS1; TNFAIP3; TRIB3; TSPYL2; VEGFA; WNK2; WNT5A; ZFYVE28* |
| mitochondrial electron transport, NADH to ubiquinone | 0.000000007 | *MT-ND1; MT-ND2; MT-ND4; MT-ND4L; MT-ND5; MT-ND6* |
| cell adhesion | 0.000000008 | *ABL2; AMIGO2; AXL; CD44; CLDN1; COL12A1; COL1A1; COL6A3; COL7A1; CTGF; DCBLD2; DSP; DST; EFNB2; ERBB3; FAT1; FAT3; ICAM1; IL8; ITGA2; ITGAM; KIF26B; LAMA5; LAMB3; MACF1; MEGF10; MMP14; NRG1; PLAU; PTPRJ; RELN; RND3; SDK2; SNAI2; SPOCK1; TGFB2; THBS1; VEGFA; WNT5A* |
| circadian rhythm | 0.00000001 | *BHLHE40; BHLHE41; DDC; EGR1; IL6; NR1D1; PER1; TIMELESS* |
| regulation of cell growth | 0.0000001 | *CDKN2C; CTGF; DCBLD2; ENPP1; FOXM1; HBEGF; IGFBP3; MMP14; NRG1; PLXNA4; PTPRJ; SEMA7A; SPOCK1; TGFB2; TRPV2; TSPYL2; WNT5A* |
| DNA replication | 0.0000002 | *C10orf2; CDC6; CDT1; CTGF; ID3; IL6; KIAA0101; LIG1; MCM2; MCM3; MCM5; PDGFA; POLE; RRM2; TICRR; TK1; TSPYL2* |
| cell communication | 0.0000003 | *ABL2; AEN; AGPAT9; ANGPTL4; ARC; ARHGEF2; ASB2; ATF3; ATF4; ATP6V0D2; ATP6V0E2; AXL; BDKRB2; BIRC3; CCNA2; CD44; CDC42EP3; COL1A1; CRABP2; CSRNP1; CTGF; CTH; DCBLD2; DDC; DDIT3; DKK1; DLK1; DMBT1; DNAH17; DOCK9; DRAXIN; DSP; DST; DUSP10; DUSP4; DUSP5; EFNB2; EGR1; ENPP1; ERBB3; F3; FAT1; FBN1; FOSL1; FOXM1; GABARAPL1; GADD45A; GADD45B; GDF15; GJB2; GPR84; GPRC5C; GTSE1; HBEGF; HMOX1; ICAM1; IGF2; IGFBP3; IL11; IL1A; IL1R1; IL23A; IL6; IL8; IRF1; ITGA2; ITGAM; JUNB; KALRN; KLF10; KLF6; KLF9; LAMA5; LPAR6; LRP1; MACF1; MLL2; MT2A; NFAT5; NFKBIB; NGFR; NQO1; NR1D1; NR1D2; NRG1; NUAK2; PDGFA; PER1; PHLDA3; PLA2G4C; PLAU; PLAUR; PLK2; PLK3; PLXNA4; PMAIP1; PPP1R15A; PRKAB1; PTPRJ; RELN; RND3; RRAD; SEMA7A; SNAI2; SPAG5; SPOCK1; SPRY4; STC2; STK17B; TBC1D5; TENM4; TGFB2; THBS1; TIPARP; TNFAIP3; TNFRSF10D; TRAF1; TRIB3; TSPYL2; TXNRD1; UNC5B; VDR; VEGFA; WNK2; WNT5A; ZFYVE28* |
| chemotaxis | 0.0000004 | *ABL2; COL1A1; COL6A3; DPYSL3; DRAXIN; EFNB2; F3; FOSL1; IL23A; IL6; IL8; ITGA2; ITGAM; NGFR; PDGFA; PLAU; PLAUR; PLXNA4; PTPRJ; RELN; SEMA7A; TGFB2; THBS1; UNC5B; VEGFA; WNT5A* |
| regulation of cyclin-dependent protein kinase activity | 0.0000006 | *CCNA2; CCNL1; CDC6; CDKN2C; GADD45A; PKMYT1; PLK1; TNFAIP3* |
| skeletal system development | 0.0000006 | *CD44; COL11A2; COL12A1; COL1A1; CSRNP1; CTGF; DLK1; FBN1; IGF2; KIAA1217; KLF10; LUM; MMP13; MMP9; SNAI2; TGFB2; THBS1; TIPARP; VDR; WNT5A* |
| G1/S transition of mitotic cell cycle | 0.0000007 | *CDC6; CDCA5; CDKN2C; CDT1; E2F1; GTSE1; MCM2; MCM3; MCM5; PKMYT1; PLK2; PLK3; POLE; RRM2* |
