# Supplementary material for: Identification of Putative Target Genes of the Transcription Factor RUNX2
Source: PLoS One. 2013 Dec 12;8(12):e83218. doi: 10.1371/journal.pone.0083218 (PMC3861491; doi:10.1371/journal.pone.0083218)
Supplement: Table S6 — Alleles ancestral in Neandertals and Denisovans, and derived in present-day humans. Derived alleles are defined as being observed at high frequency (>=90%) in the 1000 Genomes project. Positions on chromosome 6 in the human genome (hg19), the ancestral, and the derived allele are shown. A region between the first transcription start site of RUNX2 +10,000 bases upstream, and the last transcription end site +10,000 bases has been considered. P2 = Promoter 2 of RUNX2, regulatory = regulatory element as defined in the ENSEMBL database. (DOCX) [file pone.0083218.s009.docx]

**Table S6.** Alleles ancestral in Neandertals and Denisovans, and derived in present-day humans. Derived alleles are defined as being observed at high frequency (>=90%) in the 1000 Genomes project. Positions on chromosome 6 in the human genome (hg19), the ancestral, and the derived allele are shown. A region between the first transcription start site of *RUNX2* +10,000 bases upstream, and the last transcription end site +10,000 bases has been considered. P2 = Promoter 2 of *RUNX2*, regulatory = regulatory element as defined in the ENSEMBL database.

| **Position** | **dbSNP137** | **ancestral allele** | **derived allele** | **derived allele frequency** | **element** |
| --- | --- | --- | --- | --- | --- |
| 45358413 | rs11966902 | G | A | 0.93 |  |
| 45359876 | rs11968177 | C | A | 0.93 |  |
| 45363796 | rs10498760 | A | C | 0.94 |  |
| 45364117 | rs75142593 | C | T | 0.94 |  |
| 45365687 | rs56789892 | C | T | 0.93 |  |
| 45366207 | rs11966462 | C | T | 0.94 | regulatory |
| 45366946 | rs17288355 | G | A | 0.94 |  |
| 45370897 | rs58571027 | C | A | 0.93 |  |
| 45377777 | rs75864224 | G | A | 0.93 |  |
| 45383421 | rs72557349 | AC | A | 1.00 |  |
| **45389234** | **rs7751427** | **G** | **A** | **0.93** | **P2, regulatory** |
| **45389289** | **rs7771980** | **C** | **T** | **0.93** | **P2, regulatory** |
| 45395920 | rs3805818 | G | A | 0.93 |  |
| 45405134 | rs17288411 | A | C | 0.94 | regulatory |
| 45419646 | rs142148065 | A | G | 1.00 |  |
| 45423919 | rs116147910 | G | A | 0.99 |  |
| 45427417 | rs115855930 | C | T | 0.99 |  |
| 45434465 | rs7769140 | C | T | 0.99 | regulatory |
| 45437504 | rs115751427 | A | G | 0.99 |  |
| 45441643 | rs74583790 | T | G | 0.99 |  |
| 45442383 | rs115682936 | T | G | 0.99 | regulatory |
| 45446051 | rs115525602 | T | G | 0.99 | regulatory |
| 45452298 | rs6925038 | C | T | 0.98 |  |
| 45461935 | rs73737440 | G | A | 0.93 |  |
| 45468056 | rs184940965 | A | G | 1.00 |  |
| 45478025 | rs142196141 | A | G | 1.00 |  |
| 45478116 | rs151208475 | A | C | 1.00 |  |
| 45478456 | rs146966735 | A | T | 1.00 |  |
| 45527132 | rs1200425 | A | G | 1.00 |  |
