# Supplementary material for: Identification of Putative Target Genes of the Transcription Factor RUNX2
Source: PLoS One. 2013 Dec 12;8(12):e83218. doi: 10.1371/journal.pone.0083218 (PMC3861491; doi:10.1371/journal.pone.0083218)
Supplement: Table S7 — Culture conditions, transfection kits, transfection programs applied for each cell line. Cell Line Nucleofector™ Kits: V (VVCA-1003), L (VCA-1005), T (VCA-1002), R (VCA-1001), Mammalian Fibroblast (VPI-1002). Invitrogen media and supplements: A-MEM (12492-013), F12 (21765-029), D-MEM (31966-047), McCoy’s 5A medium (22330-070), D-MEM/F12 (21041-033). Invitrogen supplements: Fetal bovine serum (FBS, 10270-106), 100x Glutamine (25030-123), Geneticin® (G418, 10131-027), 100x Penicillin/Streptomycin (P/S, 15070-063). (DOCX) [file pone.0083218.s010.docx]

# Table S7. Culture conditions, transfection kits, transfection programs applied for each cell line. Cell Line Nucleofector™ Kits: V (VVCA-1003), L (VCA-1005), T (VCA-1002), R (VCA-1001), Mammalian Fibroblast (VPI-1002). Invitrogen media and supplements: A-MEM (12492-013), F12 (21765-029), D-MEM (31966-047), McCoy’s 5A medium (22330-070), D-MEM/F12 (21041-033). Invitrogen supplements: Fetal bovine serum (FBS, 10270-106), 100x Glutamine (25030-123), Geneticin® (G418, 10131-027), 100x Penicillin/Streptomycin (P/S, 15070-063)

| **Cell line** | **No. of cells** | **Amaxa kit** | **Program** | **Culture media** |
| --- | --- | --- | --- | --- |
| **ACHN** | 1x10^6^ | V | T-020 | D-MEM, 10% FBS, P/S |
| **HeLa-S3** | 5x10^5^ | R | I-013 | D-MEM, 10% FBS, P/S |
| **HepG2** | 1x10^6^ | V | T-028 | D-MEM, 10% FBS, P/S |
| **hFOB1.19** | 8x10^5^ | Mammalian Fibroblast | A-024 | D-MEM/F-12, 10% FBS, G418 (300µg/ml), P/S |
| **IMR-32** | 1x10^6^ | L | C-005 | A-MEM/F12, 10% FBS, Glutamine, P/S |
| **Saos-2** | 1x10^6^ | V | D-024 | McCoy’s 5a medium, 10% FBS, P/S |
| **SH-SY5Y** | 1x10^6^ | V | X-005 | A-MEM/F12, 10% FBS, Glutamine, P/S |
| **SK-N-SH** | 1x10^6^ | V | X-005 | A-MEM/F12, 10% FBS, Glutamine, P/S |
| **U-2 Os** | 1x10^6^ | V | X-001 | McCoy’s 5a medium, 10% FBS, P/S |
| **U-87 MG** | 5x10^5^ | T | U-029 | D-MEM, 10% FBS, P/S |
